# Can you trust clinical practice guidelines for laparoscopic surgery? A systematic review of clinical practice guidelines for laparoscopic surgery

**DOI:** 10.1007/s13304-021-01168-3

**Published:** 2021-09-14

**Authors:** Jeffrey Leung, Jonathan Leong, Kenneth Au Yeung, Bo Zhen Hao, Aled McCluskey, Yusuf Kayani, Brian R. Davidson, Kurinchi S. Gurusamy

**Affiliations:** 1 Division of Surgery and Interventional Science, Hampstead Campus, University College London, 9th Floor, Royal Free Hospital, Rowland Hill Street, London, NW3 2PF UK; 2 Medical School, University of Birmingham, Birmingham, B296QU UK; 3 Department of Therapy, I.M. Sechenov First Moscow State Medical University, Moscow, Russian Federation

**Keywords:** Practice guideline [Publication Type], Systematic review [Publication Type], Laparoscopy

## Abstract

**Background:**

Clinical practice guidelines aim to support clinicians in providing clinical care and should be supported by evidence. There is currently no information on whether clinical practice guidelines in laparoscopic surgery are supported by evidence.

**Methods:**

We performed a systematic review and identified clinical practice guidelines of laparoscopic surgery published in PubMed and Embase between March 2016 and February 2019. We performed an independent assessment of the strength of recommendation based on the evidence provided by the guideline authors. We used the ‘Appraisal of Guidelines for Research & Evaluation II’ (AGREE-II) Tool’s ‘rigour of development’, ‘clarity of presentation’, and ‘editorial independence’ domains to assess the quality of the guidelines. We performed a mixed-effects generalised linear regression modelling.

**Results:**

We retrieved 63 guidelines containing 1905 guideline statements. The median proportion of ‘difference in rating’ of strength of recommendation between the guideline authors and independent assessment was 33.3% (quartiles: 18.3%, 55.8%). The ‘rigour of development’ domain score (odds ratio 0.06; 95% confidence intervals 0.01–0.48 per unit increase in rigour score; *P* value = 0.0071) and whether the strength of recommendation was ‘strong’ by independent evaluation (odds ratio 0.09 (95% confidence intervals 0.06–0.13; *P* value < 0.001) were the only determinants of difference in rating between the guideline authors and independent evaluation.

**Conclusion:**

A considerable proportion of guideline statements in clinical practice guidelines in laparoscopic surgery are not supported by evidence. Guideline authors systematically overrated the strength of the recommendation (i.e., even when the evidence points to weak recommendation, guideline authors made strong recommendations).

**Supplementary Information:**

The online version contains supplementary material available at 10.1007/s13304-021-01168-3.

## Introduction

### Laparoscopic surgery

Laparoscopic surgery has become the preferred technique in many fields of surgery. According to the National Cancer Data Repository (NCDR), there was an increase in the use of laparoscopy from 10 to 28.4% between 2006 and 2008 for colorectal interventions in the United Kingdom.[1]. According to the American College of Surgeons National Surgical Quality Improvement Program database, the number of laparoscopic colorectal procedures increased from 3114 to 51,611, representing a 165.7% increase over the 10-year period of 2005–2014 in USA [2]. There was an increase in laparoscopic colorectal procedures in all categories including age, BMI, and American Society of Anaesthesiologists (ASA) category [2]. This increase is driven by the belief that laparoscopic surgery is superior to open surgery, especially for short-term outcomes and patient quality of life in many fields of surgery [3–5]. However, laparoscopic surgery may not be suitable for some procedures: laparoscopic pancreaticoduodenectomy is associated with more complication-related deaths [6]. Regardless of the evidence, laparoscopic surgery has become the commonest approach for some procedures like cholecystectomy [7].

### Clinical practice guidelines

There are varied definitions of clinical practice guidelines. The current Institute of Medicine defines clinical Practice Guidelines as “statements that include recommendations intended to optimise patient care that are informed by a systematic review of evidence and an assessment of the benefits and harms of alternative care options” [8]. Guidelines are usually developed by expert panels and conferences [9] to improve the process of care, quality, and outcome of treatment while reducing treatment costs [10].

### Recommendation instruments in guidelines

Guideline developers usually use a formal recommendation scheme to provide a grade of recommendation for each guideline statement. This is done to provide the guideline users a sense with information on the reliability and quality of the guidelines [11]. The most widely known formal instruments are Grading of Recommendations, Assessment, Development and Evaluation (GRADE), Oxford Centre for Evidence-Based Medicine Methodology, and the Scottish Intercollegiate Guidelines Network (SIGN). Some guideline developers also opt to use their own systems. GRADE was developed in 2004 and has been widely used since [12]. It is endorsed by medical organisations such as the World Health Organisation (WHO) and the National Institute of Health and Care Excellence (NICE) [11, 13]. The purpose of the GRADE system is to provide a systematic method of grading clinical evidence and to develop guidelines based on clinical evidence [14]. Oxford methodology was developed with the purpose of ensuring that people considering the information are aware of the flaws in the evidence [15]. SIGN was developed to link evidence to recommendations [16]. SIGN is also endorsed by NICE and it contributes to the UK national policies [17]. Although these methods for guideline production have assisted authors in producing and updating clinical practice guidelines, the decision for publication and dissemination of these clinical practice guidelines is based on traditional peer review without necessitating a formal assessment of guideline quality.

### Assessing guideline quality with the AGREE-II Tool

The Appraisal of Guidelines, Research and Evaluation (AGREE) research team developed a tool to assess the methodological quality of clinical practice guidelines in 2003 [18]. This was updated in 2009 and published as the AGREE-II in 2010 [19]. AGREE-II has become an internationally accepted standard for evaluation of the methodological quality of clinical practice guidelines [20]. In the AGREE-II tool, there are 23 items classified under six domains: scope and purpose, stakeholder involvement, rigour of development, clarity of presentation, applicability, and editorial independence. There are, in addition, two overall assessments (overall quality of guideline and recommendation of guideline for use) [19].

### Justification for this research

The plethora of recommendation schemes available to develop guidelines means that there is variation in the grades of recommendations in the different guidelines. Variation in the grades of recommendations may be because of the lack of standardisation in developing the guidelines [11]. This variation can potentially confuse guideline users [21].

In a pilot study, we reviewed four guidelines related to laparoscopic surgery containing 191 guideline statements: in 38–74% of guideline statements, the grades of recommendation of the guideline authors were different from those made independently using the supporting information provided by the guideline authors [22]. There have been previous systematic reviews assessing the quality of clinical practice guidelines in other areas of healthcare [23, 24]. These systematic reviews highlighted that a considerable proportion of guidelines were not evidence-based [23, 24]. However, there has been no previous systematic research about the quality of laparoscopic guidelines or whether the poor methodological quality of guidelines impacts on the strength of recommendation. Therefore, it is necessary to establish the true extent of the problem in laparoscopic guidelines and how this relates to the quality of the clinical practice guidelines.

We hypothesised that the differences in grades of recommendation of the guideline authors from those made independently using the supporting information could be related to the rigour of development, clarity of presentation, and/or editorial independence domains of AGREE-II.

### Justification for the choice of the three domains within AGREE-II

Of the six domains of AGREE-II, the three domains: rigour of development, clarity of presentation, and/or editorial independence are the domains most likely to be related to disagreement in the grade of recommendation between the guideline authors and an independent assessment based on the supporting information provided. One of the key aspects of the domain ‘stakeholder involvement’ (‘patient’s views and preferences’) is a part of developing the grade of the recommendation, and, therefore, was incorporated into developing the strength of recommendation. The remaining two domains scope and purpose and applicability are not related to the strength of the recommendation, as these domains cover the generalisability of the guidelines, likely barriers and facilitators to implementation, strategies to improve uptake, and resource implications of applying the guideline.

### Research objectives

The objectives of this research were to find the extent of differences between the grades of recommendation made by the guideline authors and an independent assessment of the same guideline statements based on the supporting evidence provided by the guideline authors and explore the reasons for these differences (‘differences in grading’).

## Methods

We followed the PRISMA guidance in the reporting [25]. A list of abbreviations used in this report are available in Supplementary file S1 (Supplementary file S1 Appendix 1).

### Study protocol and deviations from protocol

A study protocol is available at https://zenodo.org/record/3660007. The major deviations from the protocol include hierarchical logistic regression to account for the correlation between guideline statements within the guideline and including the strength of recommendation as a variable to explore reasons for the differences in grading.

### Search methods for identification of studies

#### Databases searched

We searched PubMed and Embase using free text and controlled vocabulary terms (MeSH). We modified the search strategy for laparoscopy from a Cochrane systematic review [26] and that for clinical practice guidelines from published search filters for guidelines [27] (Supplementary file S1 Appendix 2). We then combined both search strategies using the Boolean operator ‘AND’ to identify clinical practice guidelines in laparoscopy from these electronic databases.

### Inclusion and exclusion criteria

We included the guidelines for the systematic review if they met the following inclusion criteria:

- Related to any laparoscopic surgery.
- An evidence summary or citation of studies for each statement was presented to allow reclassification of statements using GRADE.
- A clear recommendation instrument was implemented with well-explained criteria for each grade.
- Each statement in the guidelines received a specific recommendation rather than an overall recommendation for the entire guideline.
- Published between 1^st^ March 2016 and 28^th^ February 2019.

We included all guidelines that met the above inclusion criteria and did not have any specific exclusion criteria. We did not apply any language restrictions. We translated the guidelines which were not published in English using translation software (Google translate).

### Guideline identification

Two reviewers among the research team independently screened the references and selected the guidelines for full-text evaluation and inclusion. We resolved any differences through discussion.

### Data collection and management

#### Data collection

Two reviewers among the research team (the first author and one of the next four authors) independently collected the following data from each included guideline.

- Citation (each guideline was provided a unique id).
- Scheme used for making the levels of recommendations (for example, Oxford Centre for Evidence-Based Medicine: Levels of Evidence, GRADE method of recommendations).
- Guideline statements.
- Level of recommendation for each guideline statement as stated by the authors.
- Supporting evidence for each guideline statement.

#### Conversion of different schemes to GRADE method of recommendations

We converted each scheme of recommendation used in the guidelines to the current GRADE method of recommendation, similar to the conversion table that we used for the pilot study [22]. The conversion table is shown in Supplementary file S1 Appendix 3.

#### Development of independent level of recommendation for each guideline statement

Two trained reviewers independently assessed the supporting evidence and calculated the level of recommendation for each guideline statement. We used the supporting evidence as stated by the authors to arrive at the recommendation level and did not search for any additional citations or information. In other words, we did not check whether the guideline authors had misunderstood or misquoted the information from the citations, or whether they excluded some key citations on the topic to support their own views. We then graded the results as ‘Strong’ or ‘Weak’ for each guideline statement by considering the following four factors used to arrive at a recommendation as per GRADE guidance [12, 28–31].

- Balance between desirable and undesirable effects.
- Quality of the evidence.
- Costs or resources utilised.
- Values and preferences.

#### Calculation of scores

Two review authors (the first author and one of the next four authors) gave scores for each item in the domains for each guideline statement independently according to the instruction manual of the AGREE-II Tool. We gave all guideline statements in a guideline the same score for some domains such as ‘editorial independence’, while we gave different scores for different guideline statements within the same guideline for the rigour and clarity domains. The scores of each item in the domain were then scaled using the following equation according to the instructions in the AGREE-II Tool Manual [19]:

$$\frac{\mathrm{Obtained score}-\mathrm{Minimum possible score}}{\mathrm{Maximum possible score}-\mathrm{Minimum possible score}}.$$

### Analysis

We summarised the characteristics as median and interquartiles. We did not log-transform the data as the log transformation did not improve the normality of distribution. We used a mixed-effects generalised linear regression modelling using binary distribution and logit link in the GLIMMIX procedure of Statistical Analysis System (SAS) software (version 9.4). Initially, we ran the following two models.

- *Model A* Fixed-effects for the three AGREE-II domains (rigour of development, clarity of presentation, and editorial independence), the system used for grading the recommendation (for example, GRADE system, Oxford methodology, SIGN methodology, etc.), and the grade of recommendation by independent assessment.
- *Model B* Same as model A, but in addition, random-effects for guideline ID (i.e., reference to the guideline publication to which the guideline statement belongs) to account for potential correlation of domain scores within guideline statements for a set of guideline statements developed by a group of guideline authors.

Based on the results of these analyses, we ran two further models.

- *Model C* Fixed-effects for the ‘rigour’ AGREE-II domain and the grade of recommendation by independent assessment.
- *Model D* Same as model C, but in addition, random-effects for guideline ID

We chose the best model based on model fit (corrected Akaike Information Criterion (AICC)) and the area under the receiver-operating characteristics curve (C-statistic). When the results of two models were similar, we chose the simpler model (i.e., the model with fewer variables). We considered a *P* value of < 0.05 as statistically significant. The data and codes used for analysis are available in Supplementary file S2.

## Results

### Results of the search

**Fig. 1:**
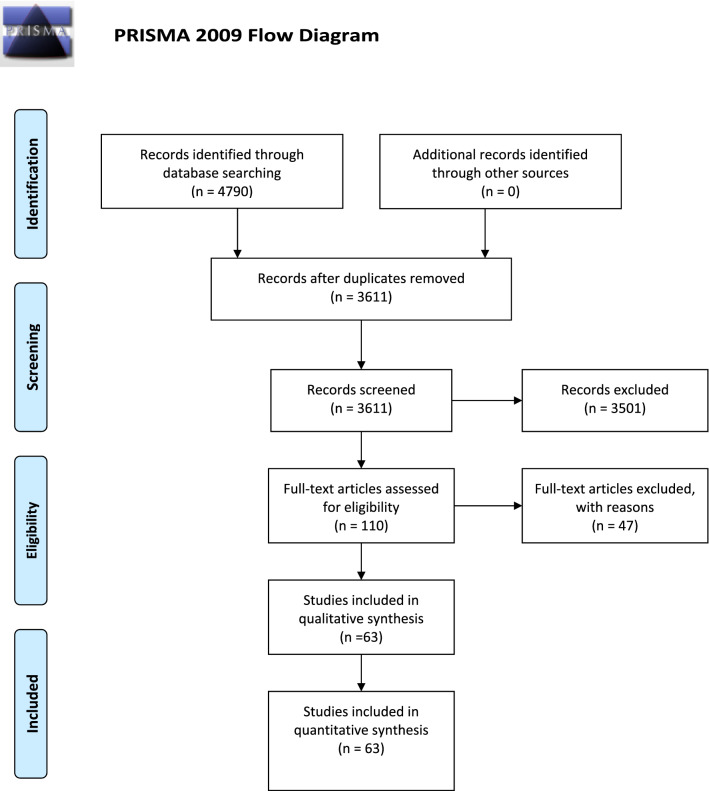
PRISMA flow diagram for the results of the search. Flow of information through four phases of a systematic review: Identification, screening, eligibility, and included studies

We identified a total of 4790 references through the electronic searches in PubMed (*n* = 1940) and Embase (*n* = 2850). We excluded 1179 duplicates and 3501 clearly irrelevant references through review of titles and/or abstracts. Of the 110 retrieved references, all were identified as clinical practice guidelines. We further excluded 47 references due to a variety of reasons: the majority either lacked recommendation systems or instruments or had unclear grading criteria for the recommendation instrument. We listed the detailed reasons under ‘Characteristics of excluded studies’ (Supplementary file S1 Appendix 4). In total, 63 clinical practice guidelines fulfilled the inclusion criteria. The Preferred Reporting Items for Systematic Reviews and Meta-Analyses (PRISMA) flow diagram is shown in Fig. 1, created from a template developed by the PRISMA group [32].

Details of the clinical practice guidelines used in the included studies are available in Supplementary file S1 Appendix 3.

### Characteristics of included guidelines

In total, we included 63 guidelines consisting of 1905 statements. The number of statements from each guideline varied from 2 to 152 statements (median and interquartile: 22 [11]). Eleven recognised systems of grading the classifications (GRADE, Oxford Methodology, SIGN, US Preventative Task Force, Haute Autorité de santé (French national health authority), AWMF guidance manual and rules for guideline development, The Canadian Task Force on Preventive Health Care, Methodological Manual of the National Guidelines System, Classification system CDC, Infectious Diseases Society of America-United States Public Health Service Grading System, and ASCO recommendation), and other bespoke systems (i.e., the guideline developers used a classification system which was not used by previous guideline developers) were used in these 63 guidelines. The most frequent classification system used was GRADE system: this system was used in 26 clinical practice guidelines. All the remaining classification systems were used in fewer than ten clinical practice guidelines. The number of times each guideline classification system was used is available in Supplementary file 1 Appendix 5) and in a graphical format in Supplementary file S1 Appendix 6.

### Difference in rating of strength of recommendation between the guideline authors and independent evaluation

The ratings of the strength of recommendation by the guideline authors and the independent assessment (by some authors of this manuscript as detailed in the methods) are available in the Supplementary file S3 (Sheet 'Guidelines'). A summary of the disagreements between the guideline authors and independent assessment is shown in Supplementary file S3 (Sheet 'Disagreements') and in graphical format in Supplementary file S1 Appendix 7. The median of the proportion of disagreement of recommendations between guideline authors and independent evaluation was 33.3% (18.3%, 55.8%).

### Scores of the AGREE-II tool domains

The scaled scores for the AGREE-II tool domains for each guideline statement are available in the Supplementary file S3 (Sheet 'Guidelines'). The median and interquartile scaled score for the ‘rigour of development’, ‘clarity of presentation’, and 'editorial independence' domains were 0.43 (0.38, 0.47), 0.72 (0.58, 0.83), and 0.63 (0.42, 0.79), respectively.

### Results of the analysis

The output from the analysis is available in Supplementary file S4.

#### Correlation between the AGREE-II domains

The Spearman correlation coefficients between the assessed AGREE-II domains are listed in Table 1. All correlations were statistically significant from 0 at *P* value < 0.001. The correlation coefficients suggest that there was very weak correlation between the three domains assessed.

**Table 1:** Correlation between the assessed AGREE-II domains

	Rigour of development	Clarity of presentation	Editorial independence
Rigour of development	1	0.19654	0.16762
Clarity of presentation	0.19654	1	0.14919
Editorial independence	0.16762	0.14919	1

*P* value < 0.0001

### Linear regression modelling

In the initial analysis, the model including guideline ID as random-effects (in addition to including the AGREE-II domains as fixed-effects, system used for grading the recommendation, and grade of recommendation by independent assessment, i.e., Model B) had better model fit (AICC = 2093.7; *C*-statistic = 0.8167) compared to the model without the guideline ID as random-effects (Model A: AICC = 2215.60; *C*-statistic = 0.7315).

In the model B, the only statistically significant variables were rigour of development and grade of recommendation by independent assessment. In the fixed-effect analysis including only rigour of development and grade of recommendation by independent assessment (model C), the AICC and C-statistic were 2226.60 and 0.7190, respectively. In the hierarchical logistic regression analysis including only rigour of development and grade of recommendation by independent assessment as fixed-effects and guideline ID as random-effects (model D), the AICC and C-statistic were 2091.05 and 0.8151, respectively. The fit statistics of model D were similar to that of model B, indicating that the remaining variables (other than the variables included in model D) contributed very little to explaining the difference in rating by the guideline authors and independent assessment. Therefore, we have presented the results of model D. The results based on model B were similar to those of model D.

Based on the results of model D, the odds ratios of 'difference in rating' (of the strength of recommendation between the guideline author and independent assessment) were 0.06 (95% CI 0.01–0.48) per unit increase in the rigour of development (i.e., as 'rigour in development' score increased the odds of 'difference in rating' decreased implying that when the 'rigour in development' scores were higher, the classification of the strength of recommendation was more reliable) and 0.09 (95% CI 0.06–0.13) of strong versus weak recommendation by independent assessment (i.e., the odds of 'difference in rating' were lower when recommendation by independent assessment was 'strong' recommendation compared to 'weak' recommendation, implying that 'weak' recommendations were much more likely to be misclassified than 'strong' recommendations). The results of model B were similar: the odds ratios of 'difference in rating' were 0.11 (95% CI 0.01–0.90) per unit increase in the rigour of development and 0.08 (95% CI 0.06–0.12) of strong versus weak recommendation by independent assessment.

**Fig. 2:**
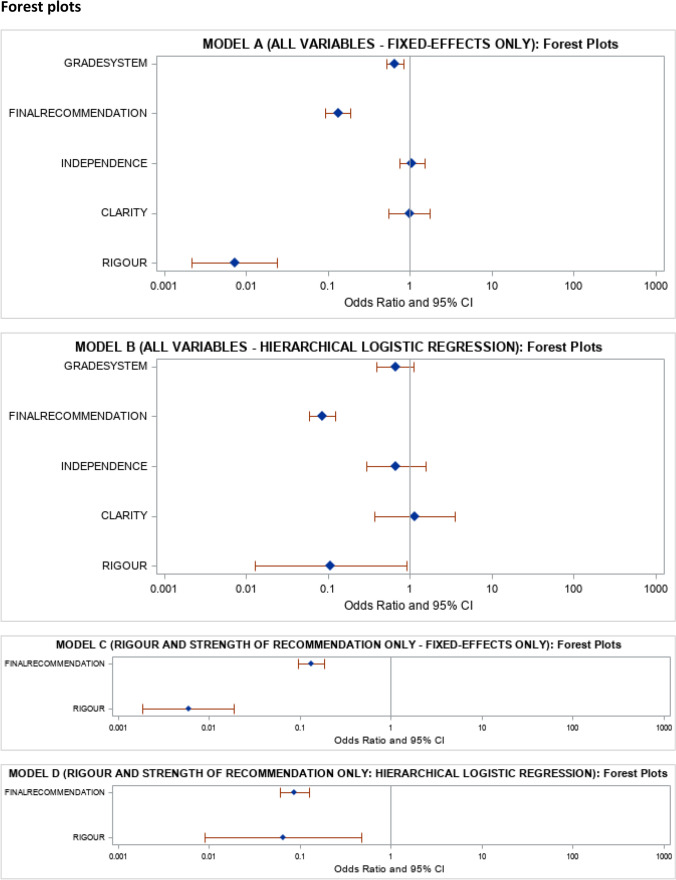
Forest plots of the odds ratios of the different independent variables from the four different models

The odds ratios of the different independent variables from different models are shown as forest plots in Fig. 2.

## Discussion

Our research identified 63 guidelines related to laparoscopy consisting of 1905 guideline statements. A number of classification systems for grading guidelines were used; the most common classification system used was the GRADE system.

The strength of recommendation of approximately a third of the guideline statements was different between the guideline developers and an independent assessment based on the evidence provided by the guideline developers. Regression analysis showed that this was related to the rigour of development (i.e., the differences in rating were fewer when the scores of rigour of development were higher) and the strength of recommendation (i.e., the differences in rating were more when the independent assessment indicated 'weak' recommendation than when the independent assessment indicated 'strong' recommendation) of the AGREE-II system of assessing clinical practice guidelines. This indicates that the guideline authors were systematically overrating the strength of the recommendation (i.e., even when the evidence points to weak recommendation, guideline authors made strong recommendations).

The systematic overrating of the strength of recommendation has major implications in terms of uptake of the treatment (by healthcare professionals and patients), use of resources (to implement the recommendation), medical negligence lawsuits, and in further research performed to address the uncertainty. When the recommendation is strong, the GRADE working group has suggested that the clinical practice guideline developers use terms such as 'clinicians should…' or 'should not…' (depending on whether the intervention should or should not be recommended to patients); when the recommendation is weak, the GRADE working group has suggested clinical practice guideline developers use terms such as 'clinicians might…' [28]. This obviously has implications on how strongly clinicians recommend the treatment to patient and the treatment choice of patients. Communicating the strength of recommendation accurately is a fundamental aspect of the shared-decision making process: if the strength of recommendation is flawed by systematic overrating of the strength of evidence or poor rigour of development (of the guideline), this makes the whole process of shared-decision making a flawed and futile exercise with an outcome based on false guidance. The strength of recommendation also can have major implications on resource allocation, when there are limitations in funds allocated for improving the health of the population (which is the situation in most countries): strong recommendations may have to be implemented by diverting funds from treatments where there is less evidence of clinical benefit. Therefore, flawed systematic overrating of the strength of evidence and poor rigour of development (of the guideline) may lead to poor clinical decision-making and decrease the overall health of the population.

In medical negligence lawsuits, when a clinician has failed to recommend a treatment option which has a strong guideline recommendation, this would be considered a breach of duty of care and could lead to prosecution and suspension from the GMC. A flawed systematic overrating of the strength of evidence and poor rigour of development (of the guideline) may result in a major injustice for a clinician who interprets the evidence better than the guideline developers.

Flawed systematic overrating of the strength of evidence can also impair research performed to address the uncertainties: this may result in perpetuating the wrong beliefs and further impairment of shared-decision making and appropriate resource-use.

While the impact of the systematic overrating of the strength of evidence can be understood, the reason for this systematic overrating of the strength of evidence has not been investigated in this study. Some potential reasons for guideline authors getting the guidelines wrong include an unbalanced guideline developers panel with healthcare professionals favouring new treatments, vested interests of the guideline developers, lack of formal consensus methods to develop guidelines, opaque and inconsistent methods for rating evidence, failure to capture the impact of differing patients’ values and perspectives, publication bias, and absence of adequate peer-review procedures [33]. Based on the anecdotal experience of the senior author of this research (KG), guideline developers also appear to align themselves closely to guidelines on the same topic by a different guideline developer group. While it is appropriate to consider the evidence used by the different guideline developer group, aligning the guidelines to other guidelines on the same topic to avoid confusion for health professionals is clearly inappropriate and misleading: this simply perpetuates the mistakes in guideline development, hides the uncertainty in the decision, and prevents researchers from seeking the truth. Further research is necessary into how the rigour of guideline development can be improved and systematic overrating of evidence avoided by guideline authors, without resulting in unsustainable increase in resource use.

### Strengths and weaknesses

The major strengths of this research include a thorough and systematic search of the literature to understand the prevalence of the misclassification of the strength of recommendations in laparoscopic surgery guidelines, duplicate and independent study selection and data extraction to minimise errors, and use of the appropriate analysis to identify the potential reasons for misclassification of the strength of recommendation in clinical practice guidelines.

The most important limitation is that we used the supporting evidence as stated by the guideline authors to arrive at the recommendation level and did not search for any additional citations or information. In other words, we did not check whether the guideline authors had misunderstood or misquoted the information from the citations, or whether they excluded some key citations on the topic to support their own views. If the supporting evidence used by the guideline authors does not take into account all the available evidence or if the interpretation of the supporting evidence by the guideline authors is found to be wrong, the 'rigour of development' scores will be lower than that estimated by our research team and the difference in rating (of the grade of recommendation between that of the guideline authors and the independent evaluation) will be even more than what we found in this research. This is likely to strengthen the association between poor rigour of development and misclassification of the strength of recommendation. Therefore, the findings of this research should be interpreted as minimum effect (rather than the true effect) of poor rigour of development on the misclassification of the strength of recommendation. In other words, what we found in this research is likely to be only the 'tip of the iceberg'.

The other major limitation of this research is that we converted the strength of recommendation from other classification systems of grading to the GRADE system of classification of recommendations. This conversion was done by the author team and not by formal consensus methods. However, it should be noted that our research did not demonstrate any evidence that the disagreement between the strength of recommendation between the guideline authors and independent evaluation was related to the guideline classification system used. Besides, we have provided reasons for this conversion and the data used for analysis in the supplementary material, which will allow other researchers to develop their own conversion methods to analyse the impact of different conversion methods on the results of our research.

### Agreements and disagreements with other similar research

There have been previous systematic reviews assessing the quality of clinical practice guidelines in other areas of healthcare [23, 24]. These systematic reviews highlighted that a considerable proportion of guidelines were not evidence-based [23, 24]. However, there has been no previous research into how this poor methodological quality of guidelines impacts on the strength of recommendation. Our research clearly shows that the rigour of development impacts on the strength of recommendation. Furthermore, our research showed that there was a systematic overestimation of the strength of recommendation by guideline developers (i.e., even when the evidence points to weak recommendation, guideline authors made strong recommendations). Since these are new findings, it is necessary to find out if similar findings are obtained in a different set of laparoscopy guidelines before firm conclusions can be reached.

### Applicability of findings

These findings are applicable only for clinical practice guidelines related to laparoscopy. We are unable to comment on whether these findings are applicable in clinical practice guidelines in other fields of healthcare.

## Conclusion

There were considerable differences between the grades of recommendation made by the guideline authors and an independent assessment of the same guideline statements based on the supporting evidence provided by the guideline authors. These differences are associated with poor rigour of development of the clinical practice guideline. Guideline authors systematically overrated the strength of the clinical recommendations (i.e., even when the evidence points to weak recommendation, guideline authors made strong recommendations).

## Supplementary Information

Below is the link to the electronic supplementary material.

Supplementary file1 (DOCX 150 kb)

Supplementary file2 (SAS 89 kb)

Supplementary file3 (XLSX 409 kb)

Supplementary file4 (PDF 2192 kb)

Supplementary file5 (DOC 64 kb)

## Data Availability

All data and materials used are available for review.

## Code availability

All codes used for the generation of statistics are available.
