# Supplementary material for: Can you trust clinical practice guidelines for laparoscopic surgery? A systematic review of clinical practice guidelines for laparoscopic surgery
Source: Updates Surg. 2021 Sep 14;74(2):391–401. doi: 10.1007/s13304-021-01168-3 (PMC8995291; doi:10.1007/s13304-021-01168-3)

# Appendix 1 List of abbreviations used

AGREE-II: Appraisal of Guidelines for Research & Evaluation II

AIC: Akaike Information Criterion

AICC: Corrected Akaike Information Criterion

ASA: American Society of Anaesthesiologists

GRADE: Grading of Recommendations, Assessment, Development and Evaluation

NICE: National Institute of Health and Care Excellence

NCDR: National Cancer Data Repository

PRISMA: Preferred Reporting Items for Systematic Reviews and Meta- Analyses

SAS: Statistical Analysis System

SIGN: Scottish Intercollegiate Guidelines Network

# Appendix 2 Search strategy

| **Database** | **Search strategy** |
| --- | --- |
| PubMed | ((laparoscop* OR celioscop* OR coelioscop* OR abdominoscop* OR peritoneoscop*) AND (Clinical pathway[mh] OR Clinical protocol[mh] OR Consensus[mh] OR Consensus development conferences as topic[mh] OR Critical pathways[mh] OR Guidelines as topic [Mesh:NoExp] OR Practice guidelines as topic[mh] OR Health planning guidelines[mh] OR guideline[pt] OR practice guideline[pt] OR consensus development conference[pt] OR consensus development conference, NIH[pt] OR position statement*[tiab] OR policy statement*[tiab] OR practice parameter*[tiab] OR best practice*[tiab] OR standards[ti] OR guideline[ti] OR guidelines[ti] OR ((practice[tiab] OR treatment*[tiab]) AND guideline*[tiab]) OR CPG[tiab] OR CPGs[tiab] OR consensus*[tiab] OR ((critical[tiab] OR clinical[tiab] OR practice[tiab]) AND (path[tiab] OR paths[tiab] OR pathway[tiab] OR pathways[tiab] OR protocol*[tiab])) OR recommendat*[ti] OR (care[tiab] AND (standard[tiab] OR path[tiab] OR paths[tiab] OR pathway[tiab] OR pathways[tiab] OR map[tiab] OR maps[tiab] OR plan[tiab] OR plans[tiab])) OR (algorithm*[tiab] AND (screening[tiab] OR examination[tiab] OR test[tiab] OR tested[tiab] OR testing[tiab] OR assessment*[tiab] OR diagnosis[tiab] OR diagnoses[tiab] OR diagnosed[tiab] OR diagnosing[tiab])) OR (algorithm*[tiab] AND (pharmacotherap*[tiab] OR chemotherap*[tiab] OR chemotreatment*[tiab] OR therap*[tiab] OR treatment*[tiab] OR intervention*[tiab]))) |
| Embase | (laparoscop* or celioscop* or coelioscop* or abdominoscop* or peritoneoscop*).af  exp laparoscopic surgery/  1or2  exp clinical pathway/  exp clinical protocol/  exp consensus/  exp consensus development conference/  exp consensus development conferences as topic/  critical pathways/  exp practice guideline/  guidelines as topic/  practice guidelines as topic/  health planning guidelines/  (position statement* or policy statement* or practice parameter* or best practice*).ti,ab,kw.  (standards or guideline or guidelines).ti,kw.  ((practice or treatment* or clinical) adj guideline*).ab.  (CPG or CPGs).ti.  consensus*.ti,kw.  consensus*.ab. /freq=2  ((critical or clinical or practice) adj2 (path or paths or pathway or pathways or protocol*)).ti,ab,kw.  recommendat*.ti,kw.  (care adj2 (standard or path or paths or pathway or pathways or map or maps or plan or plans)).ti,ab,kw.  (algorithm* adj2 (screening or examination or test or tested or testing or assessment* or diagnosis or diagnoses or diagnosed or diagnosing)).ti,ab,kw.  (algorithm* adj2 (pharmacotherap* or chemotherap* or chemotreatment* or therap* or treatment* or intervention*)).ti,ab,kw.  Or/4-24  3 and 25  |

# Appendix 3 Conversion of different schemes to GRADE method of recommendations

| **Guideline system** | **Guideline** | **Recommendation scheme used by guideline author** | **Equivalent GRADE system + reason** |
| --- | --- | --- | --- |
| Grade | European Hernia Society Guidelines On Prevention and Treatment of Parastomal Hernias (Antoniou et al., 2018) | Strong | Strong  Reason: Based on high quality evidence, and if clinicians are certain that benefits do or not do outweigh risks and burdens |
|  |  | Weak | Weak  Reason: Based on weak evidence, or if clinicians admits there are uncertainties |
|  |  | No recommendation | Weak  Reason: Based on expert opinion entirely, with no evidence |
|  | Clinical Practise Guidelines for Laparoscopic Hysterectomy for Benign Indications (Sandberg et al., 2017) | Strong | Strong  Reason: The explanation of the criteria of each grade is the same as the one used in GRADE |
|  |  | Weak | Weak  Reason: The explanation of the criteria of each grade is the same as the one used in GRADE |
|  | Tokyo Guidelines 2018: surgical management of acute cholecystitis: safe steps in laparoscopic cholecystectomy for acute cholecystitis (with videos) (Wakabayashi et al., 2018) | 1 | Strong  Reason: The guideline made it clear that it was an update from the TG 2013 guideline, which stated it used GRADE as the recommendation system and where recommendation 1= strong and recommendation 2= weak vice versa. The criteria of each grade is identical to the criteria of GRADE |
|  |  | 2 | Weak  Reason: stated above |
|  |  | No recommendation | Weak  Reason: stated above |
|  | International guidelines for groin hernia management (HerniaSurge, 2018) | Strong | Strong  Reason: Did not give exact criteria but it stated that it followed the GRADE system, with consensus and cross-validation with Agree-II and oxford methodology |
|  |  | Weak | Weak  Reason: Same as above, did not give exact criteria but it stated it used the GRADE system and the recommendation grade of ‘Strong’ and ‘Weak’ was identical |
|  | Systematic review of the limited evidence for different surgical techniques at benign hysterectomy: A clinical guideline initiated by the Danish Health Authority (Sloth et al., 2017) | ↑↑  A strong recommendation for | Strong  Reason: As stated in the guideline that it used a GRADE system, it did not give further explanation and we would assume a strong recommendation equals to a strong recommendation as equivalent as well. |
|  |  | ↓↓  A strong recommendation against | Strong  Reason: Same as above |
|  |  | ↑  A weak recommendation for | Weak  Reason: same as above |
|  |  | ↓  A weak recommendation against | Weak  Reason: same as above |
|  |  | √  Good practice | Weak  Reason: In the explanation of this symbol, it indicated that it represented weak evidence but were recommended by experts. This would constitute a weak recommendation. |
|  | Japanese Society for Cancer of the Colon and Rectum (JSCCR) guidelines 2016 for the treatment of colorectal cancer (Watanabe et al., 2018) | 1 | Strong  Reason: In the guideline it indicated 1= strong, also the appraisal of the evidence and explanation of the grade of recommendation is identical to the GRADE criteria. |
|  |  | 2 | Weak  Reason: Same as above, with 2= weak recommendation |
|  | Management of intra-abdominal infections: recommendations by the WSES 2016 consensus conference (Sartelli et al., 2017) | 1A | Strong  Reason: In this guideline, the authors divided the ‘strong’ and ‘weak’ recommendation of GRADE into six grades, where 1 indicated strong and 2 indicates weak recommendation. A, B and C were the grades for the quality of evidence. 1A was given an equivalent of ‘strong’ as it indicated high quality evidence + benefits outweigh risk and burden |
|  |  | 1B | Strong  Reason: 1B indicated strong and moderate quality evidence. |
|  |  | 1C | Strong  Reason: A strong recommendation was still given as strong recommendation despite weak evidence, the benefits still clearly outweighed the risks and burden. |
|  |  | 2A | Weak  Reason: Despite logically a high quality evidence would constitute strong recommendation, the face that the authors made it clear it would be a weak recommendation justified this equivalent grade |
|  |  | 2B | Weak  Reason: The evidence was of moderate quality and there was uncertainty in the recommendation |
|  |  | 2C | Weak  Reason: Weak or lack of evidence with no uncertainty in the recommendation |
|  | Bologna guidelines for diagnosis and management of adhesive small bowel obstruction (ASBO): 2017 update of the evidence-based guidelines from the world society of emergency surgery ASBO working group (Ten Broek et al., 2018) | I | Strong  Reason: The guideline used GRADE as the system and simply changed the wording of strong and weak to I and II |
|  |  | II | Weak  Reason: Same as above |
|  | The use of 3D laparoscopic imaging systems in surgery: EAES consensus development conference 2018 (Arezzo et al., 2018) | High | Strong  Reason: Stated clearly they used GRADE as the system and ‘high’ ‘low’ were just different wording to ‘strong’ and ‘weak |
|  |  | Low | Weak  Reason: Same as above |
|  | SAGES guidelines for the use of laparoscopy during pregnancy (Pearl et al., 2017) | Strong | Strong  Reason: Same wording and clearly stated GRADE was used |
|  |  | Weak | Weak  Reason: Same as above |
|  | Evidence-based guidelines for vaginal hysterectomy of the International Society for Gynecologic Endoscopy (ISGE) (Chrysostomou et al., 2018) | 1A | Strong  Reason: In this guideline, the authors divided the ‘strong’ and ‘weak’ recommendation of GRADE into six grades, where 1 indicated strong and 2 indicates weak recommendation. A, B and C were the grades for the quality of evidence. 1A was given an equivalent of ‘strong’ as it indicated high quality evidence + benefits outweigh risk and burden |
|  |  | 1B | Strong  Reason: 1B indicated strong and moderate quality evidence |
|  |  | 1C | Strong  Reason: A strong recommendation was still given as strong recommendation despite weak evidence, the benefits still clearly outweighed the risks and burden |
|  |  | 2A | Weak  Reason: Despite logically a high quality evidence would constitute strong recommendation, the face that the authors made it clear it would be a weak recommendation justified this equivalent grade |
|  |  | 2B | Weak  Reason: The evidence was of moderate quality and there was uncertainty in the recommendation |
|  |  | 2C | Weak  Reason: Weak or lack of evidence with no uncertainty in the recommendation |
|  | Evaluation and management of traumatic diaphragmatic injuries: A Practice Management Guideline from the Eastern Association for the Surgery of Trauma (McDonald et al., 2018) | Strong | Strong  Reason: Same wording as GRADE and it stated in the guideline it followed the GRADE system |
|  |  | Conditional | Weak  Reason: Different wording but it meant ‘weak’ recommendation |
|  | The Association of Coloproctology of Great Britain and Ireland consensus guidelines in surgery for inflammatory bowel disease (Brown et al., 2018) | A | Strong  Reason: High quality evidence with consistent findings supporting the recommendation |
|  |  | B | Strong  Reason: Moderate quality evidence (without RCTs) together with consistent findings |
|  |  | C | Weak  Reason: Moderate quality evidence without consistent findings |
|  |  | D | Weak  Reason: Little to no evidence |
|  |  | GP | Weak  Reason: Expert opinion, clinical evidence only |
|  | Clinical practice guideline for the prevention of venous thromboembolic events during pregnancy (Bogotana Association of Obstetrics and Gynecology (ASBOG), 2017) | Fuerte a favour  (Strong for) | Strong  Reason: Reason: High quality evidence with consistent findings supporting the recommendation |
|  |  | Fuerte en contra  (Weak against) | Strong  Reason: Reason: Moderate quality evidence (without RCTs) together with consistent findings |
|  |  | Débil a favor  (Weak for) | Weak  Reason: Reason: Moderate quality evidence without consistent findings |
|  |  | Debil en contra  (Weak against) | Weak  Reason : Reason: Little to no evidence |
|  |  | Punto de buena práctica clínica  (Expert opinion) | Weak  Reason: Reason: Expert opinion, clinical evidence only |
|  | Clinical practice guideline for enhanced recovery after colon and rectal surgery from the American Society of Colon and Rectal Surgeons (ASCRS) and Society of American Gastrointestinal and Endoscopic Surgeons (SAGES) (Carmichael et al., 2017) | 1A | Strong  Reason: High quality evidence with clear benefits. All evidence from RCTs and the recommendation would apply to most circumstances |
|  |  | 1B | Strong  Reason: Moderate evidence with clear benefits |
|  |  | 1C | Strong  Reason: Despite low quality evidence (observational studies or case series), there were multiple sources of evidence and the benefits were clear |
|  |  | 2A | Weak  Reason: Despite high quality evidence, the benefits were unclear |
|  |  | 2B | Weak  Reason: Moderate quality evidence with unclear benefits |
|  |  | 2C | Weak  Reason: Low quality evidence with a lot of uncertainty |
|  | The American Society of Colon and Rectal Surgeons Clinical Practice Guidelines for the Treatment of Colon Cancer (Vogel et al., 2017) | 1A | Strong  Reason: High quality evidence with clear benefits. All evidence from RCTs and the recommendation would apply to most circumstances |
|  |  | 1B | Strong  Reason: Moderate evidence with clear benefits |
|  |  | 1C | Strong  Reason: Despite low quality evidence (observational studies or case series), there were multiple sources of evidence and the benefits were clear |
|  |  | 2A | Weak  Reason: Despite high quality evidence, the benefits were unclear |
|  |  | 2B | Weak  Reason: Moderate quality evidence with unclear benefits |
|  |  | 2C | Weak  Reason: Low quality evidence with a lot of uncertainty |
|  | Asian-Pacific Association for the Study of the Liver (APASL) consensus guidelines on invasive and non-invasive assessment of hepatic fibrosis: a 2016 update (Shiha et al., 2017) | 1 | Strong  Reason: Stated GRADE was used as the guideline system, and in the explanation of criteria they gave the same criteria as GRADE. From this we could infer 1= ‘strong’ and 2= ‘weak’ |
|  |  | 2 | Weak  Reason: Stated above |
|  | EASL Clinical Practice Guidelines on the prevention, diagnosis and treatment of gallstones (European Association for the Study of the Liver . Electronic address, 2016) | Strong | Strong  Reason: Same wording and explanation as GRADE |
|  |  | Weak | Weak  Reason: Stated above |
|  | Updated guideline on the management of common bile duct stones (CBDS) (Williams et al., 2017) | Strong | Strong  Reason: Exact same wording and explanation as GRADE |
|  |  | Weak | Weak  Reason: stated above |
|  | The Management of Primary Aldosteronism: Case Detection, Diagnosis, and Treatment: An Endocrine Society Clinical Practice Guideline (Funder et al., 2016) | 1 | Strong  Reason: Stated GRADE was used as the guideline system, and in the explanation of criteria they gave the same criteria as GRADE. From this we could infer 1= ‘strong’ and 2= ‘weak’ |
|  |  | 2 | Weak  Reason: Stated above |
|  | SAGES guidelines for laparoscopic ventral hernia repair (Earle et al., 2016) | Strong | Strong  Reason: Exact same wording and explanation as GRADE |
|  |  | Weak | Weak  Reason: Stated above |
|  | 2017 WSES guidelines for the management of iatrogenic colonoscopy perforation (de'Angelis et al., 2018) | 1A | Strong  Reason: ‘1’ and ‘2’ Indicated strong or weak recommendation whereas ‘A’, ‘B’,’C’ indicated quality of evidence. The criteria of each grade was quoted directly from the GRADE handbook |
|  |  | 1B | Strong  Reason: Same as above |
|  |  | 1C | Strong  Reason: Same as above |
|  |  | 2A | Weak  Reason: Same as above |
|  |  | 2B | Weak  Reason: Same as above |
|  |  | 2C | Weak  Reason: Same as above |
|  | 2017 update of the WSES guidelines for emergency repair of complicated abdominal wall hernia (Birindelli et al., 2017) | 1A | Strong  Reason: ‘1’ and ‘2’ Indicated strong or weak recommendation whereas ‘A’, ‘B’,’C’ indicated quality of evidence. The criteria of each grade was quoted directly from the GRADE handbook |
|  |  | 1B | Strong  Reason: Same as above |
|  |  | 1C | Strong  Reason: Same as above |
|  |  | 2A | Weak  Reason: Same as above |
|  |  | 2B | Weak  Reason: Same as above |
|  |  | 2C | Weak  Reason: Same as above |
|  | The International Liver Transplant Society Guidelines on Living Liver Donation [34] | Class 1 | Strong  Reason: In the guideline it stated ‘Class 1’ was equivalent to ‘strong’, and it was related to effectiveness and benefits of the procedure or treatment |
|  |  | Class 2 | Weak  Reason: There was conflicting evidence for the recommendation |
|  |  | Class 3 | Weak  Reason: Recommendation might not be useful or harmful with lack of evidence and therefore would not recommend the recommendation |
|  | Evidence-based clinical practice guidelines for cholelithiasis 2016 (Tazuma et al., 2017) | 1 | Strong  Reason: Stated GRADE was used as the guideline system, and in the explanation of criteria they gave the same criteria as GRADE. From this we could infer 1= ‘strong’ and 2= ‘weak’ |
|  |  | 2 | Weak  Reason: As stated above |
|  | Closure of the perineal defect after abdominoperineal excision for rectal adenocarcinoma – ACPGBI Position Statement (Foster et al., 2018) | ↑↑ | Strong  Reason: The guideline stated this symbol meant strong recommendation for using an intervention |
|  |  | ↑? | Weak  Reason: The guideline stated this symbol meant weak recommendation for using an intervention |
|  |  | ↓? | Weak  Reason: The guideline stated this symbol meant weak recommendation for not using an intervention |
|  |  | ↓↓ | Strong  Reason: The guideline stated this symbol meant strong recommendation for not using an intervention |
| Oxford Methodology | EAU Guidelines on Interventional Treatment for Urolithiasis (Turk et al., 2016) | A | Strong  Reason: The guideline stated the grading followed the evidence grading of oxford methodology. It would constitute a strong recommendation as it suggested high quality evidence |
|  |  | B | Strong  Reason: Moderate evidence with clear benefits |
|  |  | C | Weak  Reason: Weak evidence with multiple case series with extrapolations from evidence |
|  |  | D | Weak  Reason: It represented expert opinion or lack of consistency in evidence which was equivalent to a weak recommendation |
|  | WSES Jerusalem guidelines for diagnosis and treatment of acute appendicitis (Di Saverio et al., 2016) | A | Strong  Reason: The guideline stated the grading followed the evidence grading of oxford methodology. It would constitute a strong recommendation as it suggested high quality evidence |
|  |  | B | Strong  Reason: Moderate evidence with clear benefits |
|  |  | C | Weak  Reason: Weak evidence with multiple case series with extrapolations from evidence |
|  |  | D | Weak  Reason: It represented expert opinion or lack of consistency in evidence which was equivalent to a weak recommendation |
|  | 2016 WSES guidelines on acute calculous cholecystitis (Ansaloni et al., 2016) | A | Strong  Reason: The guideline stated the grading followed the evidence grading of oxford methodology. It would constitute a strong recommendation as it suggested high quality evidence |
|  |  | B | Strong  Reason: Moderate evidence with clear benefits |
|  |  | C | Weak  Reason: Weak evidence with multiple case series with extrapolations from evidence |
|  |  | D | Weak  Reason: It represented expert opinion or lack of consistency in evidence which was equivalent to a weak recommendation |
|  | Hysterectomy for Benign Uterine Disease (Neis et al., 2016) | A | Strong  Reason: The guideline stated the grading followed the evidence grading of oxford methodology. It would constitute a strong recommendation as it suggested high quality evidence |
|  |  | B | Strong  Reason: Moderate evidence with clear benefits |
|  |  | C | Weak  Reason: Weak evidence with multiple case series with extrapolations from evidence |
|  |  | D | Weak  Reason: It represented expert opinion or lack of consistency in evidence which was equivalent to a weak recommendation |
|  | Canadian Urological Association-Pediatric Urologists of Canada (CUA-PUC) guideline for the diagnosis, management, and follow up of cryptorchidism (Braga et al., 2017) | A | Strong  Reason: The guideline stated the grading followed the evidence grading of oxford methodology. It would constitute a strong recommendation as it suggested high quality evidence |
|  |  | B | Strong  Reason: Moderate evidence with clear benefits |
|  |  | C | Weak  Reason: Weak evidence with multiple case series with extrapolations from evidence |
|  |  | D | Weak  Reason: It represented expert opinion or lack of consistency in evidence which was equivalent to a weak recommendation |
| SIGN | The Southampton Consensus Guidelines for Laparoscopic Liver Surgery (Abu Hilal et al., 2018) | Strong | Strong  Reason: Same wording, also represented high quality evidence and clear benefits |
|  |  | Recommended best practice based on the clinical experience of the guideline development group | Strong  Reason: Clear benefits and effects for the intervention but moderate to low quality consistent evidence |
|  |  | Conditional | Weak  Reason: Unclear benefits/effects with low quality evidence |
|  | Targeted treatment of primary aldosteronism – The consensus of Taiwan Society of Aldosteronism (Huang et al., 2019) | A | Strong  Reason: High quality consistent evidence |
|  |  | B | Strong  Reason: Moderate quality consistent evidence, applicable to the majority of the population |
|  |  | C | Weak  Reason: Moderate to low quality evidence, consistent with the statement |
|  |  | D | Weak  Reason: Low quality evidence, there were inconsistencies or lack of evidence in some statements |
| US Preventative Task Force | Assessing the risk of laparoscopic morcellation of occult uterine sarcomas during hysterectomy and myomectomy: Literature review and the ISGE recommendations (Sizzi et al., 2018) | A | Strong  Reason: This recommendation grade suggested high quality evidence and substantial benefits |
|  |  | B | Weak  Reason: This recommendation grade consisted of moderate to low quality of evidence. The benefits in general outweighed the harms |
|  |  | C | Weak  Reason: Moderate to low quality of evidence and unclear benefits |
| Haute Autorité de santé (French national health authority) | Clinical practice guidelines: Synthesis of the guidelines for the surgical treatment of primary pelvic organ prolapse in women by the AFU, CNGOF, SIFUD-PP, SNFCP, and SCGP (Le Normand et al., 2017) | A | Strong  Reason: High quality evidence with strong indication of benefits outweighing the harms |
|  |  | B | Strong  Reason: Moderate quality evidence with strong indication of benefits outweighing the harm |
|  |  | C | Weak  Reason: Low quality evidence, inconsistent and unclear benefits |
|  |  | PC | Weak  Reason: Expert opinion/lack of evidence |
|  | Traitement du prolapsus génital par promontofixation laparoscopique : recommandations pour la pratique Clinique Laparoscopic sacrocolpopexy for pelvic organ prolapse: guidelines for clinical practice (Wagner et al., 2016) | A | Strong  Reason: High quality evidence with strong indication of benefits outweighing the harms |
|  |  | B | Weak  Reason: Moderate quality evidence with strong indication of benefits outweighing the harm |
|  |  | C | Weak  Reason: Low quality evidence, inconsistent and unclear benefits |
|  |  | PC | Weak  Reason: Reason: Expert opinion/lack of evidence |
| AWMF guidance manual and rules for guideline development | Short version of the S3 guideline on screening, diagnosis, therapy and follow-up of abdominal aortic aneurysms (Debus et al., 2018) | A | Strong  Reason: Highest quality of evidence, with consistent results and clear benefit outweighing harm |
|  |  | B | Weak  Reason: Moderate to low quality of evidence, uncertain effects |
|  |  | O | Weak  Reason: Very low quality of evidence or lack of evidence, no consistency in results |
| The Canadian Task Force on Preventive Health Care | No. 371-Morcellation During Gynaecologic Surgery: Its Uses, Complications, and Risks of Unsuspected Malignancy (Murji et al., 2019) | A | Strong  Reason: High quality evidence. Consistently supported the statement |
|  |  | B | Strong  Reason: Moderate quality evidence. Consistently supported the statement |
|  |  | C | Weak  Reason: Conflicting evidence |
|  |  | D | Weak  Reason: There was a fair amount of evidence against the recommendation |
|  |  | E | Weak  Reason: There was a good amount of evidence against the recommendation |
|  |  | I | Weak  Reason: Insufficient evidence |
| Methodological Manual of the National Guidelines System | Guidelines for diagnosis and treatment of fibromyomatosis (Giancarlo Conoscenti et al., 2017) | A | Strong  Reason: The description of ‘A’ grade suggested the intervention should be strongly recommended, with high quality evidence |
|  |  | B | Strong  Reason: The intervention should always be considered |
|  |  | C | Weak  Reason: A lot of uncertainty when considering the recommendation. |
|  |  | D | Weak  Reason: The statement was not recommended due to lack of balance between benefits and harms |
|  |  | E | Weak  Reason: The intervention/procedure was strongly discouraged |
|  | No. 193-Laparoscopic Entry: A Review of Techniques, Technologies, and Complications (Vilos et al., 2017) | A | Strong  Reason: There was good evidence to recommend the procedure/statement |
|  |  | B | Strong  Reason: There was moderate to low quality evidence to recommend the procedure |
|  |  | C | Weak  Reason: There was conflicting or insufficient evidence to support or discourage the procedure/statement |
|  |  | D | Weak  Reason: There was moderate to low quality evidence to discourage the implementation of the statement |
|  |  | E | Weak  Reason: There was good evidence to discourage the implementation of the statement |
|  |  | I | Weak  Reason: Insufficient evidence |
|  | No. 238-Supracervical Hysterectomy (Kives and Lefebvre, 2018) | A | Strong  Reason: There was good evidence to recommend the procedure/statement |
|  |  | B | Strong  Reason: There was moderate to low quality evidence to recommend the procedure |
|  |  | C | Weak  Reason: There was conflicting or insufficient evidence to support or discourage the procedure/statement |
|  |  | D | Weak  Reason: There was moderate to low quality evidence to discourage the implementation of the statement |
|  |  | E | Weak  Reason: There was good evidence to discourage the implementation of the statement |
|  |  | I | Weak  Reason: Insufficient evidence |
|  | No. 341-Diagnosis and Management of Adnexal Torsion in Children, Adolescents, and Adults (Kives et al., 2017) | A | Strong  Reason: There was good evidence to recommend the procedure/statement |
|  |  | B | Strong  Reason: There was moderate to low quality evidence to recommend the procedure |
|  |  | C | Weak  Reason: There was conflicting or insufficient evidence to support or discourage the procedure/statement |
|  |  | D | Weak  Reason: There was moderate to low quality evidence to discourage the implementation of the statement |
|  |  | E | Weak  Reason: There was good evidence to discourage the implementation of the statement |
|  |  | I | Weak  Reason: Insufficient evidence |
|  | No. 345-Primary Dysmenorrhea Consensus Guideline (Burnett and Lemyre, 2017) | A | Strong  Reason: There was good evidence to recommend the procedure/statement |
|  |  | B | Strong  Reason: There was moderate to low quality evidence to recommend the procedure |
|  |  | C | Weak  Reason: There was conflicting or insufficient evidence to support or discourage the procedure/statement |
|  |  | D | Weak  Reason: There was moderate to low quality evidence to discourage the implementation of the statement |
|  |  | E | Weak  Reason: There was good evidence to discourage the implementation of the statement |
|  |  | I | Weak  Reason: Insufficient evidence |
|  | No. 362-Ovulation Induction in Polycystic Ovary Syndrome (Smithson et al., 2018) | A | Strong  Reason: There was good evidence to recommend the procedure/statement |
|  |  | B | Strong  Reason: There was moderate to low quality evidence to recommend the procedure |
|  |  | C | Weak  Reason: There was conflicting or insufficient evidence to support or discourage the procedure/statement |
|  |  | D | Weak  Reason: There was moderate to low quality evidence to discourage the implementation of the statement |
|  |  | E | Weak  Reason: There was good evidence to discourage the implementation of the statement |
|  |  | I | Weak  Reason: Insufficient evidence |
|  | No. 230-Initial Evaluation and Referral Guidelines for Management of Pelvic/Ovarian Masses (Le and Giede, 2018) | A | Strong  Reason: There was good evidence to recommend the procedure/statement |
|  |  | B | Strong  Reason: There was moderate to low quality evidence to recommend the procedure |
|  |  | C | Weak  Reason: There was conflicting or insufficient evidence to support or discourage the procedure/statement |
|  |  | D | Weak  Reason: There was moderate to low quality evidence to discourage the implementation of the statement |
|  |  | E | Weak  Reason: There was good evidence to discourage the implementation of the statement |
|  |  | I | Weak  Reason: Insufficient evidence |
|  | No. 164-Consensus Guidelines for the Management of Chronic Pelvic Pain [35] | A | Strong  Reason: There was good evidence to recommend the procedure/statement |
|  |  | B | Strong  Reason: There was moderate to low quality evidence to recommend the procedure |
|  |  | C | Weak  Reason: There was conflicting or insufficient evidence to support or discourage the procedure/statement |
|  |  | D | Weak  Reason: There was moderate to low quality evidence to discourage the implementation of the statement |
|  |  | E | Weak  Reason: There was good evidence to discourage the implementation of the statement |
|  |  | I | Weak  Reason: Insufficient evidence |
| Classification system CDC | Consensus guidelines for diagnosis, treatment and follow-up of patients with pancreatic cancer in Spain (Hidalgo et al., 2017) | A | Strong  Reason: High quality evidence with clear benefits outweighing risks |
|  |  | B | Strong  Reason: Moderate quality evidence in terms of efficacy, limited clinical benefit |
|  |  | C | Weak  Reason: Insufficient evidence |
|  |  | D | Strong  Reason: Moderate quality evidence indicating harms outweighing benefits |
|  |  | E | Strong  Reason: High quality evidence indicating harms outweighing the benefits |
|  | Guideline for the management of bile duct cancers by the Brazilian gastrointestinal tumour group (Riechelmann et al., 2016) | A | Strong  Reason: High quality evidence with clear benefits outweighing risks |
|  |  | B | Strong  Reason: Moderate quality evidence in terms of efficacy, limited clinical benefit |
|  |  | C | Weak  Reason: Insufficient evidence |
|  |  | D | Strong  Reason: Moderate quality evidence indicating harms outweighing benefits which contributed to an adverse outcome |
|  |  | E | Strong  Reason: High quality evidence indicating harms outweighing the benefits which contributed to an adverse outcome |
| Infectious Diseases Society of America-United States Public Health Service Grading System | Cervical cancer: ESMO Clinical Practice Guidelines for diagnosis, treatment and follow-up (Marth et al., 2017) | A | Strong  Reason: There was good evidence to recommend the procedure/statement |
|  |  | B | Strong  Reason: There was moderate evidence to recommend the procedure/statement |
|  |  | C | Weak  Reason: Poor or insufficient evidence |
|  |  | D | Weak  Reason: There was some evidence to discourage the implementation of the statement |
|  |  | E | Weak  Reason: There was a fair amount of evidence to discourage the implementation of the statement |
|  | Rectal Cancer: ESMO Clinical Practice Guidelines for diagnosis, treatment and follow-up (Glynne-Jones et al., 2017) | A | Strong  Reason: There was good evidence to recommend the procedure/statement |
|  |  | B | Strong  Reason: There was moderate evidence to recommend the procedure/statement |
|  |  | C | Weak  Reason: Poor or insufficient evidence |
|  |  | D | Weak  Reason: There was some evidence to discourage the implementation of the statement |
|  |  | E | Weak  Reason: There was a fair amount of evidence to discourage the implementation of the statement |
|  | SEOM Clinical Guideline of localized rectal cancer (2016) (Gonzalez-Flores et al., 2016) | A | Strong  Reason: There was good evidence to recommend the procedure/statement |
|  |  | B | Strong  Reason: There was moderate evidence to recommend the procedure/statement |
|  |  | C | Weak  Reason: Poor or insufficient evidence |
|  |  | D | Weak  Reason: There was some evidence to discourage the implementation of the statement |
|  |  | E | Weak  Reason: There was a fair amount of evidence to discourage the implementation of the statement |
| ASCO recommendation | SEOM Clinical Guideline for gastrointestinal sarcomas (GIST) (2016) (Poveda et al., 2016) | A | Strong  Reason: High quality evidence with consistent findings |
|  |  | B | Strong  Reason: Moderate quality evidence with consistent findings |
|  |  | C | Weak  Reason: Moderate quality evidence with inconsistent findings |
|  |  | D | Weak  Reason: Low quality and insufficient evidence |
|  | SEOM Clinical Guideline for the treatment of pancreatic cancer (2016) (Vera et al., 2016) | A | Strong  Reason: High quality evidence with consistent findings |
|  |  | B | Strong  Reason: Moderate quality evidence with consistent findings |
|  |  | C | Weak  Reason: Moderate quality evidence with inconsistent findings |
|  |  | D | Weak  Reason: Low quality and insufficient evidence |
| Bespoke | Management of endometriosis- CNGOF/HAS clinical practice guidelines short version (Collinet et al., 2018) | A | Strong  Reason: There was no criteria given in the guideline, but from the evidence from each statement it was inferred that this grade constituted high quality evidence and clear benefits for the procedure |
|  |  | B | Strong  Reason: Moderate to low quality evidence, but consistent findings with benefits outweighing harms |
|  |  | C | Weak  Reason: Low quality, inconsistent evidence |
|  |  | AE | Weak  Reason: Lack of evidence with expert opinion only |
|  | Recommandations pour la pratique clinique : synthèse des recommandations pour le traitement chirurgical du prolapsus génital non récidivé de la femme par l’AFU, le CNGOF, la SIFUD (Le Normand et al., 2017) | Grade A | Strong  Reason: There was high quality evidence to recommend the procedure/statement |
|  |  | Grade B | Strong  Reason: There was moderate to low quality evidence to recommend the statement |
|  |  | Grade C | Weak  Reason: There was conflicting evidence to support or discourage the procedure/statement |
|  |  | Grade D | Weak  Reason: There was insufficient evidence |
|  |  | AP | Weak  Reason: Expert opinion only |
|  | Hysterectomy for benign disease: clinical practice guidelines from the French College of Obstetrics and Gynecology (Deffieux et al., 2016) | Grade A | Strong  Reason: This grade represented high quality evidence |
|  |  | Grade B | Strong  Reason: This grade represented moderate quality evidence with consistent findings |
|  |  | Grade C | Weak  Reason: This grade represented low quality evidence |
|  |  | Expert opinion | Weak  Reason: Lack of evidence |
|  | Japan Society of Gynecologic Oncology guidelines 2017 for the treatment of uterine cervical cancer (Ebina et al., 2019) | A | Strong  Reason: The statement was strongly recommended |
|  |  | B | Strong  Reason: The statement was recommended |
|  |  | C1 | Weak  Reason: Insufficient evidence but might recommend statement |
|  |  | C2 | Weak  Reason: Insufficient evidence and might not recommend statement |
|  |  | D | Weak  Reason: Lack of evidence |
|  | Obesity Management for the Treatment of Type 2 Diabetes: Standards of Medical Care in Diabetes-2019 (American Diabetes, 2019) | A | Strong  Reason: High quality evidence mainly from RCTs, clear consistent findings |
|  |  | B | Strong  Reason: Moderate quality evidence, clear consistent findings |
|  |  | C | Weak  Reason: Low quality evidence or conflicting evidence |
|  |  | E | Weak  Reason: Expert opinion |
|  | 2017 European guideline for the management of pelvic inflammatory disease (Ross et al., 2018) | A | Strong  Reason: High quality evidence |
|  |  | B | Strong  Reason: Moderate quality evidence |
|  |  | C | Weak  Reason: Insufficient or lack of evidence |
|  | Management of rectal cancer: the 2016 French guidelines (Lakkis et al., 2017) | Grade A | Strong  Reason: High quality evidence with RCTs and meta-analyses of RCTs |
|  |  | Grade B | Strong  Reason: Moderate quality evidence with cohort studies or RCTs |
|  |  | Grade C | Weak  Reason: Only case control or retrospective studies were available |
|  |  | Grade D | Weak  Reason: Only expert opinion |
|  | Role of metformin for ovulation induction in infertile patients with polycystic ovary syndrome (PCOS): a guideline (Practice Committee of the American Society for Reproductive Medicine. Electronic address and Practice Committee of the American Society for Reproductive, 2017) | A | Strong  Reason: There was good evidence to support the recommendations |
|  |  | B | Strong  Reason: There was moderate quality evidence to support the recommendations |
|  |  | C | Weak  Reason: There was insufficient evidence |
|  | Surgical Management of Stones: American Urological association/ Endourological Society Guideline (Assimos et al., 2016a) | Strong | Strong  Reason: High quality evidence, Clear benefits |
|  |  | Moderate | Strong  Reason: Moderate quality, consistent evidence demonstrating benefits outweighing harms |
|  |  | Conditional | Weak  Reason: Low quality evidence, uncertain effects |
|  |  | Clinical principle | Weak  Reason: Low quality or lack of evidence |
|  |  | Expert opinion | Weak  Reason: Only expert opinion |
|  | Surgical Management of Stones: American Urological Association/Endourological Society Guideline, PART II (Assimos et al., 2016b) | Strong | Strong  Reason: High quality evidence, Clear benefits |
|  |  | Moderate | Strong  Reason: Moderate quality, consistent evidence demonstrating benefits outweighing harms |
|  |  | Conditional | Weak  Reason: Low quality evidence, uncertain effects |
|  |  | Clinical principle | Weak  Reason: Low quality or lack of evidence |
|  |  | Expert opinion | Weak  Reason: Only expert opinion |

# Appendix 4 Characteristics of excluded studies

| **Study** | **Reason of exclusion** |
| --- | --- |
| Laparoscopic Myomectomy and Morcellation: A Review of Techniques, Outcomes, and Practice Guidelines [36] | It was a study that included review of guidelines, but it was not a guideline itself without any statements or recommendations |
| Practical guidelines for performing laparoscopic liver resection based on the second international laparoscopic liver consensus conference [37] | A recommendation instrument was not used in this guideline and there was no recommendation given |
| LAP-VEGaS Practice Guidelines for Reporting of Educational Videos in Laparoscopic Surgery: A Joint Trainers and Trainees Consensus Statement [38] | Only the Delphi consensus method was used in this guideline. There was no recommendation given |
| Diagnostic strategies for endometriosis: CNGOF-HAS Endometriosis Guidelines [39] | This guideline had an unclear recommendation system. Despite stated that they implemented Oxford Methodology as the recommendation system, there were no information presented to explain the grading. There were also unexplained grades (E.g. NP1) and unclear statements |
| Staging and surgical approaches in gastric cancer: a clinical practice guideline[40] | This guideline did not use a recommendation instrument used |
| Surgical management of endometrioma: Different alternatives in term of pain, fertility and recurrence. CNGOF-HAS Endometriosis Guidelines [41] | This guideline mentioned the use of recommendation instrument from Haute Autorité de santé but there was no reference given and no explanation for the grading criteria. There were also unexplained grades (E.g. NP1) |
| Surgical management of deep endometriosis with colorectal involvement: CNGOF-HAS Endometriosis Guidelines  [42] | This guideline had an unclear recommendation system. Despite stated that they implemented Oxford Methodology as the recommendation system, there were no information presented to explain the grading. There were also unexplained grades (E.g. NP1) and unclear statements |
| Evaluation and management of traumatic diaphragmatic injuries: A Practice Management Guideline from the Eastern Association for the Surgery of Trauma  [43] | This guideline claimed the use of GRADE as the recommendation instrument, but there were no grades given to statements |
| First line management without IVF of infertility related to endometriosis: Result of medical therapy? Results of ovarian superovulation? Results of intrauterine insemination? CNGOF-HAS Endometriosis Guidelines[44] | There was no explanation given to how the grades were given. There was also no specific mentioning of a recommendation instrument |
| Nerve sparing techniques in deep endometriosis surgery to prevent urinary or digestive functional disorders: Techniques and results: CNGOF-HAS Endometriosis Guidelines [45] | There was no explanation given to how the grades were given. There was also no specific mentioning of a recommendation instrument |
| China Guideline for Diagnosis and Treatment of Incisional Hernia (2018 edition) [46] | This guideline did not use a recommendation instrument. No recommendations were given to statements |
| Comprehensive guidelines for the diagnosis and treatment of pancreatic cancer (2018 version) [47] | This guideline did not use a recommendation instrument. No recommendations were given to statements |
| American association of clinical endocrinologists and American College of Endocrinology comprehensive clinical practice guidelines for medical care of patients with obesity [48] | There were no evidence summaries and references provided to support the statements |
| Management of rectal cancer: the 2016 French guidelines[49] | There was no recommendations given in this guideline, only a grade of evidence was issued |
| The 2018 ISDE achalasia guidelines[50] | This guideline was based only on consensus. The consensus system/process was not explained |
| Guidelines for diagnosis and treatment on the adult groin hernia (2018 edition) [51] | This guideline did not use a recommendation instrument. No recommendations were given to statements |
| French ccAFU guidelines - Update 2018-2020: Management of kidney cancer [52] | There was no recommendations given in this guideline, only a grade of evidence was issued |
| Urinary tract involvement by endometriosis. Techniques and outcomes of surgical management: CNGOF-HAS Endometriosis Guidelines [53] | There was no explanation given to how the grades were given. There was also no specific mentioning of a recommendation instrument. There were also unexplained grades (E.g. NP1) and unclear statements |
| The International Pediatric Endosurgery Group Evidence-Based Guideline on Minimal Access Approaches to the Operative Management of Inguinal Hernia in Children [54] | There was no recommendations given in this guideline, only a grade of evidence was issued |
| American Society for Metabolic and Bariatric Surgery Integrated Health Nutritional Guidelines for the Surgical Weight Loss Patient 2016 Update: Micronutrients [55] | There were no evidence given to the statements in this guideline |
| Intrauterine contraception: CNGOF Contraception Guidelines [56] | This guideline mentioned the use of the recommendation instrument from Haute Autorité de santé but there was no reference given and no explanation for the grading criteria. There were also unexplained grades (E.g. NP1) |
| Diagnostic and treatment guidelines for gastrointestinal and genitourinary endometriosis [57] | This guideline did not implement a recommendation instrument |
| Surgery in ovarian cancer - Brazilian Society of Surgical Oncology consensus [58] | This guideline did not implement a recommendation instrument |
| Chinese experts consensus on diagnosis and treatment of non-perianal fistulating Crohn disease [59] | This guideline did not implement a recommendation instrument and no grading were given to statements |
| European Crohn's and Colitis Organisation Topical Review on Prediction, Diagnosis and Management of Fibrostenosing Crohn's Disease [60] | This guideline did not specify the use of a recommendation instrument. No explanation was given to the grades given |
| European consensus on the standardization of robotic total mesorectal excision for rectal cancer [61] | This guideline only used Delphi consensus. No recommendation instrument was implemented |
| Indications for Surgery for Obesity and Weight-Related Diseases: Position Statements from the International Federation for the Surgery of Obesity and Metabolic Disorders (IFSO) [62] | There was no grading given to the final statements |
| European Association of Urology guidelines on Renal Transplantation: Update 2018 [63] | No evidence was presented to support the statements |
| Staging and surgical approaches in gastric cancer: a clinical practice guideline [40] | No recommendations were given to the statements. No specified recommendation instruments as well |
| Strategies and surgical management of endometriosis: CNGOF-HAS Endometriosis Guidelines [64] | There was no description of use of a recommendation instrument despite gradings were given. There were also unexplained grades (E.g. NP1) |
| Minimal and mild endometriosis: Impact of the laparoscopic surgery on pelvic pain and fertility. CNGOF-HAS Endometriosis Guidelines [65] | There was no description of use of a recommendation instrument despite gradings were given. There were also unexplained grades (E.g. NP1) |
| Extragenital endometriosis: Parietal, thoracic, diaphragmatic and nervous lesions. CNGOF-HAS Endometriosis Guidelines [66] | There was no description of use of a recommendation instrument despite gradings were given. There were also unexplained grades (E.g. NP1) |
| Diagnosis accuracy of endoscopy (laparoscopy, hysteroscopy, fertiloscopy, cystoscopy, colonoscopy) in case of endometriosis: CNGOF-HAS Endometriosis Guidelines [67] | There was no description of use of a recommendation instrument despite gradings were given. There were also unexplained grades (E.g. NP1) |
| Epidemiology and diagnosis strategy: CNGOF-HAS Endometriosis Guidelines [68] | There was no description of use of a recommendation instrument despite gradings were given. There were also unexplained grades (E.g. NP1) |
| ASMBS Position Statement on medium- and long-term durability of weight loss and diabetic outcomes after conventional stapled bariatric procedures [69] | There was no recommendation systems implemented. There were no statements |
| Recommendations from the international evidence-based guideline for the assessment and management of polycystic ovary syndrome [70] | There was no evidence given to support the statements in this guideline |
| Diagnosis and management of primary aldosteronism: The endocrine society guideline 2016 revisited [71] | This study, despite the misleading name, was not a guideline, instead it was a review based on the guideline |
| IRCAD recommendation on safe laparoscopic cholecystectomy [72] | There was no implementation of any recommendation instruments |
| Recommendations for the surgical treatment of endometriosis-part 1: ovarian endometrioma [73] | There was no implementation of any recommendation instruments |
| Association of Coloproctology of Great Britain & Ireland (ACPGBI): Guidelines for the Management of Cancer of the Colon, Rectum and Anus (2017) – Introduction [74] | There was no implementation of any recommendation instruments |
| Clinical practice guidelines for pancreatic cancer 2016 from the Japan pancreas society a synopsis [75] | There was no evidence presented to support the statement |
| Management of synchronous liver metastases and the recommendations of the second St. Gallen European Organisation for Research and Treatment of Cancer consensus conference on the management of rectal cancer [75] | There was no implementation of any recommendation instruments |
| 3rd St. Gallen EORTC Gastrointestinal Cancer Conference: Consensus recommendations on controversial issues in the primary treatment of pancreatic cancer [76] | There was no implementation of any recommendation instruments |
| Management of patients with diverticulosis and diverticular disease: consensus statements from the 2nd international symposium on diverticular disease [77] | There was no evidence presented to support the statement |
| Chinese expert consensus on cytoreductive surgery and hyperthermic intraperitoneal chemotherapy for peritoneal malignancies [78] | There was no implementation of any recommendation instruments. There were also no statements |
| Clinical practice guidelines for the surgical treatment of rectal cancer: A consensus statement of the hellenic society of medical oncologists (hesmo) [79] | There was no implementation of any recommendation instruments |
| CAMIC Recommendations for Surgical Laparoscopy in Non-Obstetric Indications during Pregnancy [80] | There was no implementation of any recommendation instruments |

# Appendix 5 Number of times that each guideline classification system was used

| **Guideline system** | **Number of clinical practice guidelines using the system** |
| --- | --- |
| ASCO recommendation | 2 |
| AWMF guidance manual and rules for guideline development | 1 |
| Bespoke | 10 |
| Classification system CDC | 2 |
| Grade | 26 |
| Haute Autorité de santé (French national health authority) | 2 |
| Infectious Diseases Society of America-United States Public Health Service Grading System | 3 |
| Methodological Manual of the National Guidelines System | 8 |
| Oxford Methodology | 5 |
| SIGN | 2 |
| The Canadian Task Force on Preventive Health Care | 1 |
| US Preventative Task Force | 1 |

# Appendix 6 Number of times that each guideline classification system was used (box plot)


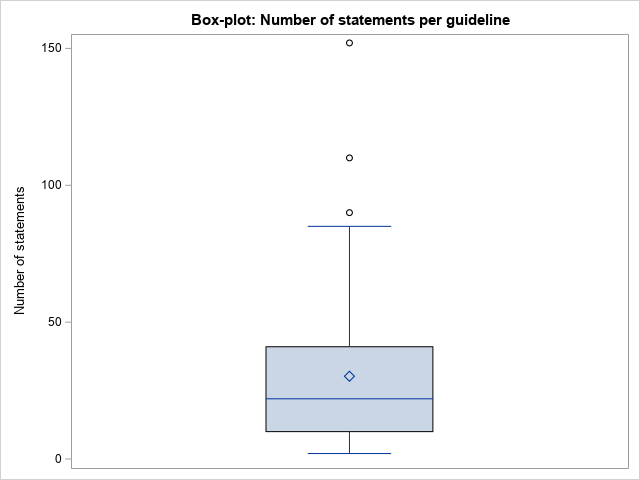


# Appendix 7 Percentage disagreement between guideline authors and independent evaluation (box plot)


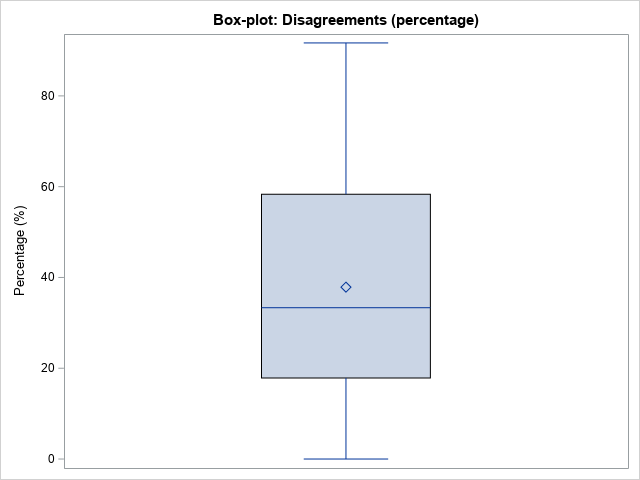

Supplement: Supplementary file 1 — Supplementary file1 (DOCX 150 kb) [file 13304_2021_1168_MOESM1_ESM.docx]
