# Supplementary material for: Can you trust clinical practice guidelines for laparoscopic surgery? A systematic review of clinical practice guidelines for laparoscopic surgery
Source: Updates Surg. 2021 Sep 14;74(2):391–401. doi: 10.1007/s13304-021-01168-3 (PMC8995291; doi:10.1007/s13304-021-01168-3)
Supplement: Supplementary file 4 — Supplementary file4 (PDF 2192 kb) [file 13304_2021_1168_MOESM4_ESM.pdf]

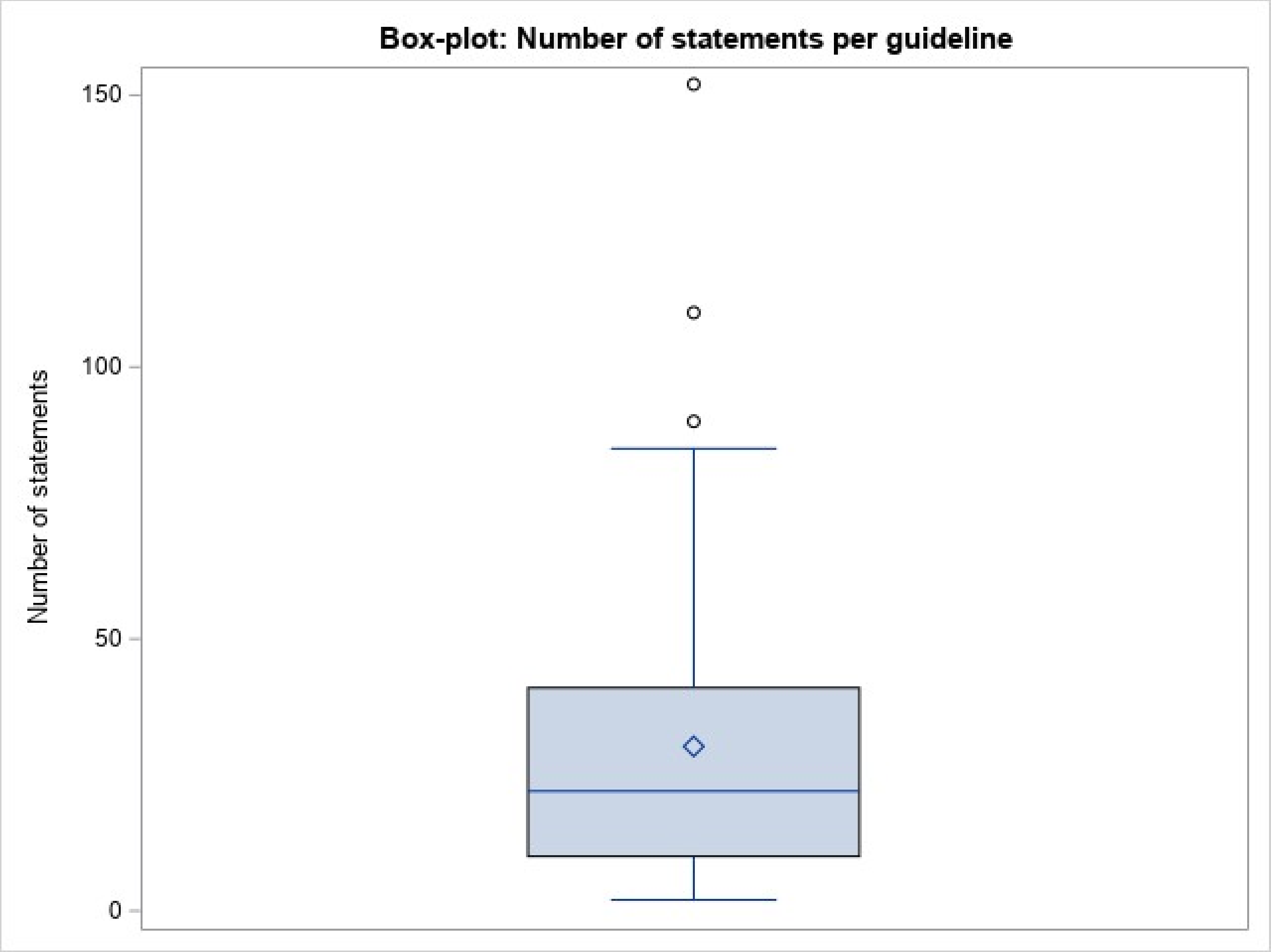
Box-plot: Number of statements per guideline
Number of statements
150
100
50
0

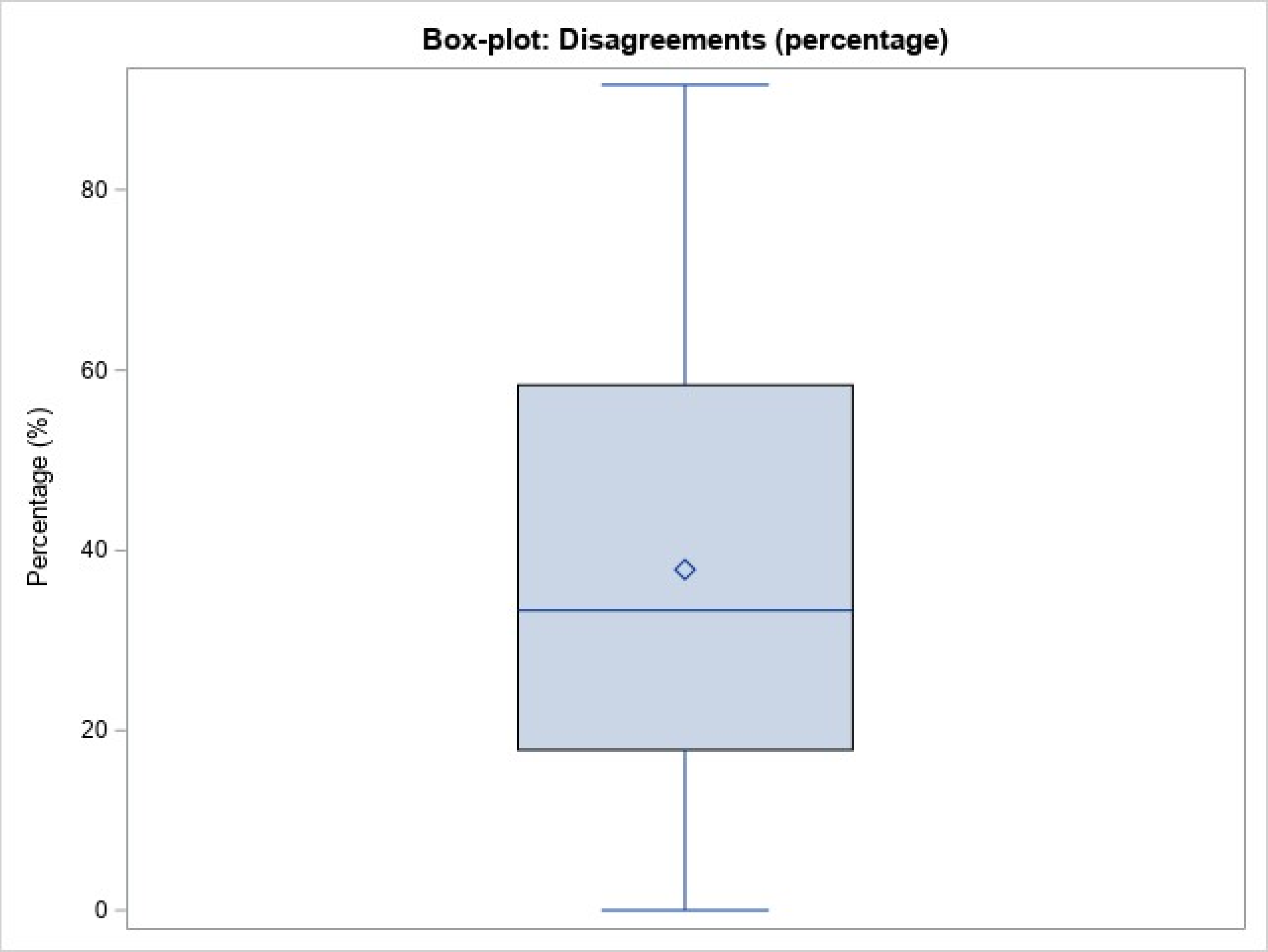
Box-plot: Disagreements (percentage)
Percentage (%)
80
60
40
20
0

Summary measures

The UNIVARIATE Procedure
Variable: rigour

Moments			
N	1905	Sum Weights	1905
Mean	0.42207458	Sum Observations	804.052083
Std Deviation	0.09464706	Variance	0.00895807
Skewness	0.11348279	Kurtosis	2.60979332
Uncorrected SS	356.426107	Corrected SS	17.0561578
Coeff Variation	22.4242502	Std Error Mean	0.0021685

Basic Statistical Measures			
Location		Variability	
Mean	0.422075	Std Deviation	0.09465
Median	0.427083	Variance	0.00896
Mode	0.406250	Range	0.79167
		Interquartile Range	0.09375

Tests for Location: Mu0=0				
Test	Statistic		p Value	
Student's t	t	194.6389	Pr > |t|	<.0001
Sign	M	952.5	Pr >= |M|	<.0001
Signed Rank	S	907732.5	Pr >= |S|	<.0001

Quantiles (Definition 5)						
				Order Statistics		
Level	Quantile	95% Confidence Limits Distribution Free		LCL Rank	UCL Rank	Coverage
100% Max	0.8750000					
99%	0.6562500	0.656250	0.770833	1878	1895	95.11
95%	0.5625000	0.552083	0.583333	1792	1830	95.35
90%	0.5208333	0.510417	0.531250	1689	1741	95.31
75% Q3	0.4687500	0.468750	0.479167	1392	1467	95.28
50% Median	0.4270833	0.416667	0.427083	910	996	95.12
25% Q1	0.3750000	0.364583	0.375000	439	514	95.28
10%	0.3229167	0.312500	0.333333	165	217	95.31

**5%**	0.2395833	0.218750	0.270833	76	114	95.35
**1%**	0.1770833	0.166667	0.187500	11	28	95.11
**0% Min**	0.0833333					

Extreme Observations			
**Lowest**		**Highest**	
**Value**	**Obs**	**Value**	**Obs**
0.0833333	1824	0.822917	13
0.0833333	1823	0.843750	9
0.0833333	1822	0.843750	12
0.0833333	1821	0.875000	7
0.0833333	1820	0.875000	8

Summary measures

The UNIVARIATE Procedure
Variable: clarity

Moments			
N	1905	Sum Weights	1905
Mean	0.69577136	Sum Observations	1325.44444
Std Deviation	0.20705789	Variance	0.04287297
Skewness	1.23064472	Kurtosis	25.9872206
Uncorrected SS	1003.83642	Corrected SS	81.6301335
Coeff Variation	29.7594727	Std Error Mean	0.004744

Basic Statistical Measures			
Location		Variability	
Mean	0.695771	Std Deviation	0.20706
Median	0.722222	Variance	0.04287
Mode	0.805556	Range	3.63889
		Interquartile Range	0.25000

Tests for Location: Mu0=0				
Test	Statistic		p Value	
Student's t	t	146.6636	Pr > |t|	<.0001
Sign	M	952.5	Pr >= |M|	<.0001
Signed Rank	S	907732.5	Pr >= |S|	<.0001

Quantiles (Definition 5)						
				Order Statistics		
Level	Quantile	95% Confidence Limits Distribution Free		LCL Rank	UCL Rank	Coverage
100% Max	3.805556					
99%	1.000000	1.000000	1.000000	1878	1895	95.11
95%	0.972222	0.972222	0.972222	1792	1830	95.35
90%	0.916667	0.916667	0.944444	1689	1741	95.31
75% Q3	0.833333	0.833333	0.833333	1392	1467	95.28
50% Median	0.722222	0.722222	0.750000	910	996	95.12
25% Q1	0.583333	0.555556	0.583333	439	514	95.28

**10%**	0.388889	0.361111	0.416667	165	217	95.31
**5%**	0.277778	0.277778	0.305556	76	114	95.35
**1%**	0.222222	0.222222	0.222222	11	28	95.11
**0% Min**	0.166667					

Extreme Observations			
**Lowest**		**Highest**	
**Value**	**Obs**	**Value**	**Obs**
0.166667	782	1.00000	1699
0.222222	1423	1.00000	1717
0.222222	1422	1.00000	1728
0.222222	1421	1.00000	1731
0.222222	1420	3.80556	1095

Summary measures

The UNIVARIATE Procedure
Variable: independence

Moments			
N	1905	Sum Weights	1905
Mean	0.5973972	Sum Observations	1138.04167
Std Deviation	0.29565039	Variance	0.08740915
Skewness	-0.6098618	Kurtosis	-0.4784655
Uncorrected SS	846.289931	Corrected SS	166.427025
Coeff Variation	49.4897511	Std Error Mean	0.00677378

Basic Statistical Measures			
Location		Variability	
Mean	0.597397	Std Deviation	0.29565
Median	0.625000	Variance	0.08741
Mode	0.750000	Range	1.00000
		Interquartile Range	0.37500

Tests for Location: Mu0=0				
Test	Statistic		p Value	
Student's t	t	88.19261	Pr > |t|	<.0001
Sign	M	872	Pr >= |M|	<.0001
Signed Rank	S	760820	Pr >= |S|	<.0001

Quantiles (Definition 5)						
				Order Statistics		
Level	Quantile	95% Confidence Limits Distribution Free		LCL Rank	UCL Rank	Coverage
100% Max	1.0000000					
99%	1.0000000	1.0000000	1.0000000	1878	1895	95.11
95%	1.0000000	1.0000000	1.0000000	1792	1830	95.35
90%	1.0000000	1.0000000	1.0000000	1689	1741	95.31
75% Q3	0.7916667	0.7500000	0.8333333	1392	1467	95.28
50% Median	0.6250000	0.6250000	0.6250000	910	996	95.12
25% Q1	0.4166667	0.3750000	0.5000000	439	514	95.28

**10%**	0.0833333	0.0416667	0.0833333	165	217	95.31
**5%**	0.0000000	0.0000000	0.0000000	76	114	95.35
**1%**	0.0000000	0.0000000	0.0000000	11	28	95.11
**0% Min**	0.0000000					

Extreme Observations			
**Lowest**		**Highest**	
**Value**	**Obs**	**Value**	**Obs**
0	1843	1	835
0	1842	1	863
0	1841	1	864
0	1840	1	865
0	1839	1	866

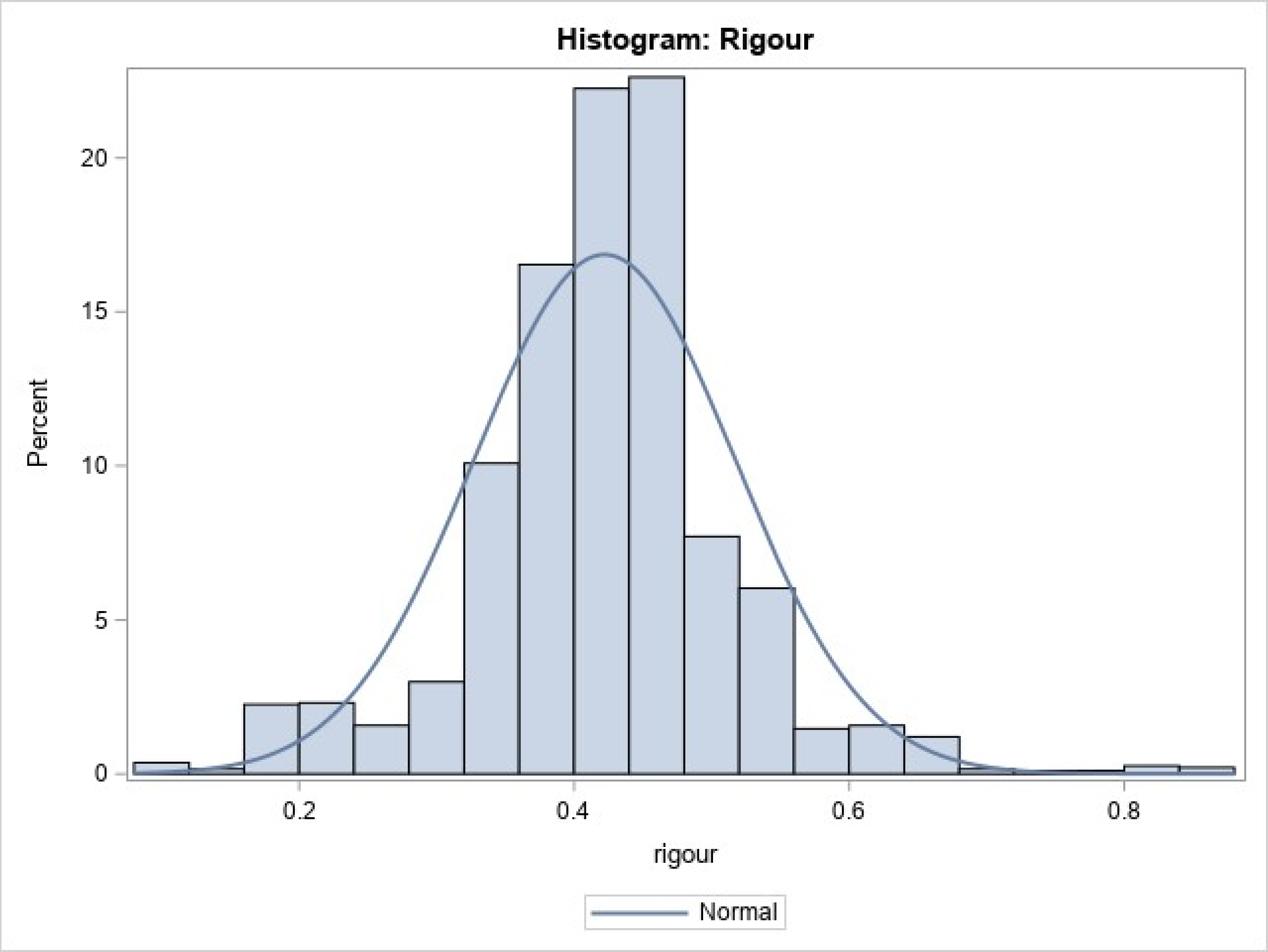
Histogram: Rigour
Percent
0
5
10
15
20
0.2
0.4
0.6
0.8
rigour
Normal

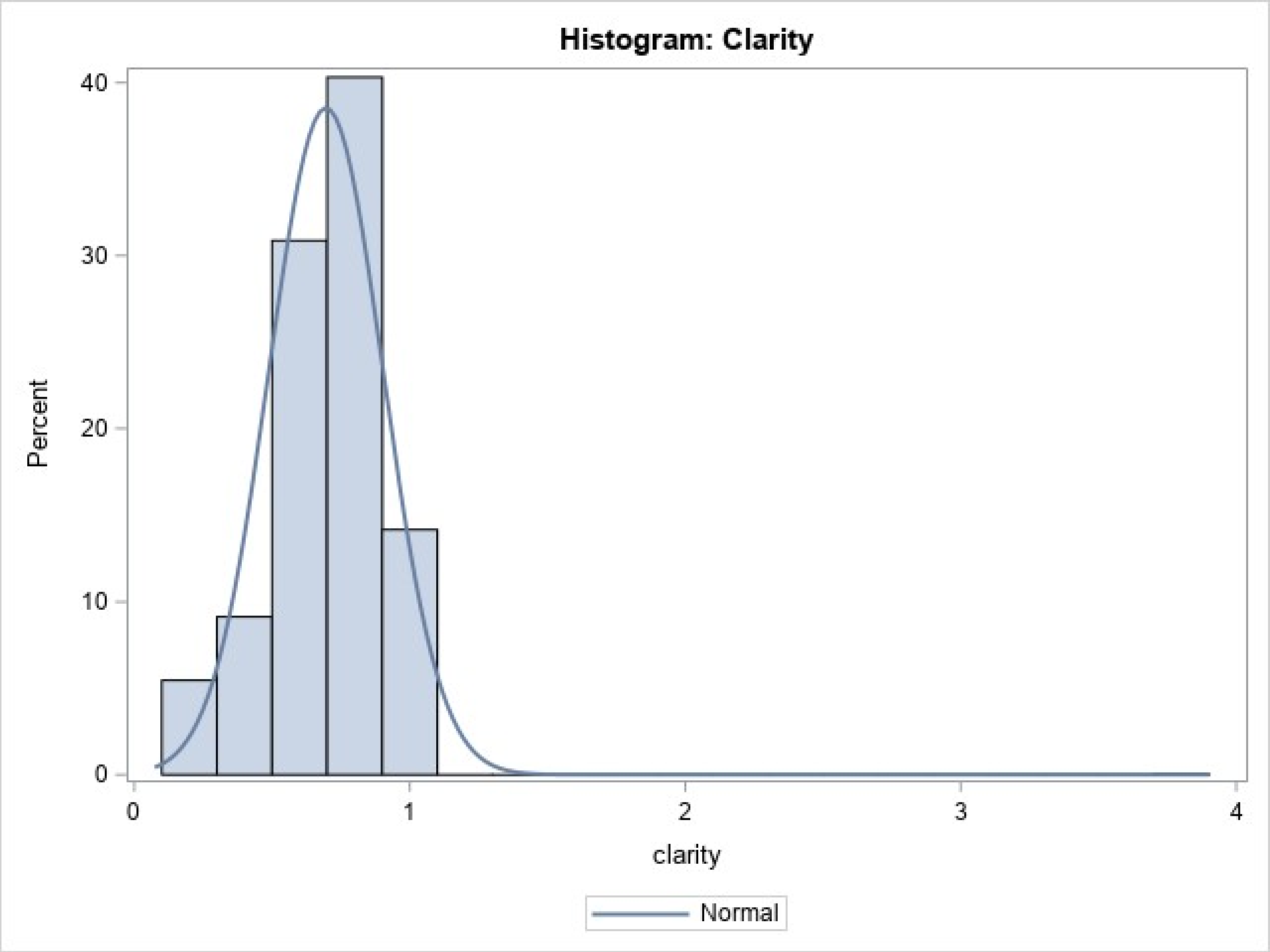
Histogram: Clarity
Percent
40
30
20
10
0
0
1
2
3
4
clarity
Normal

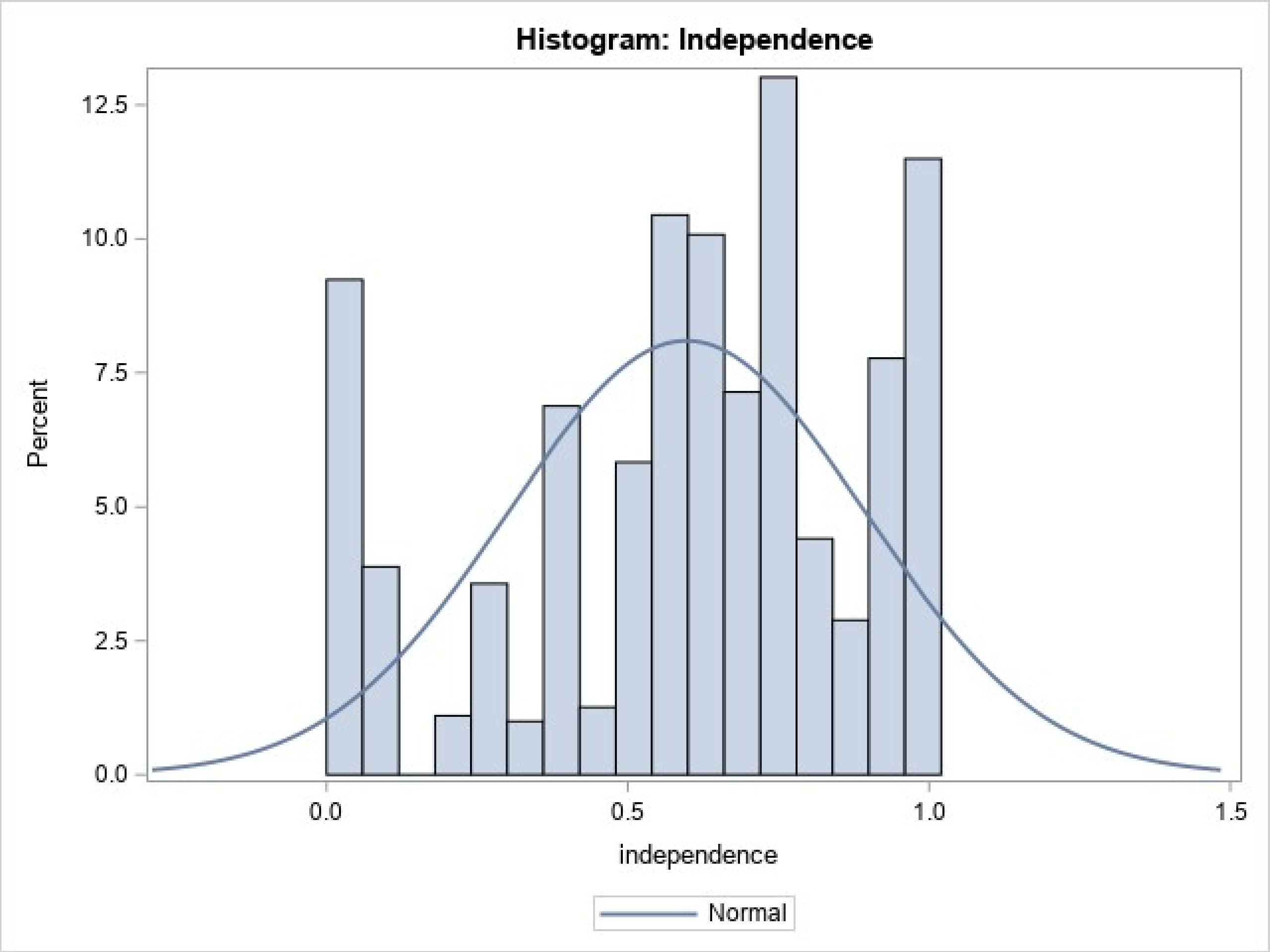
Histogram: Independence
Percent
12.5
10.0
7.5
5.0
2.5
0.0
0.0
0.5
1.0
1.5
independence
Normal

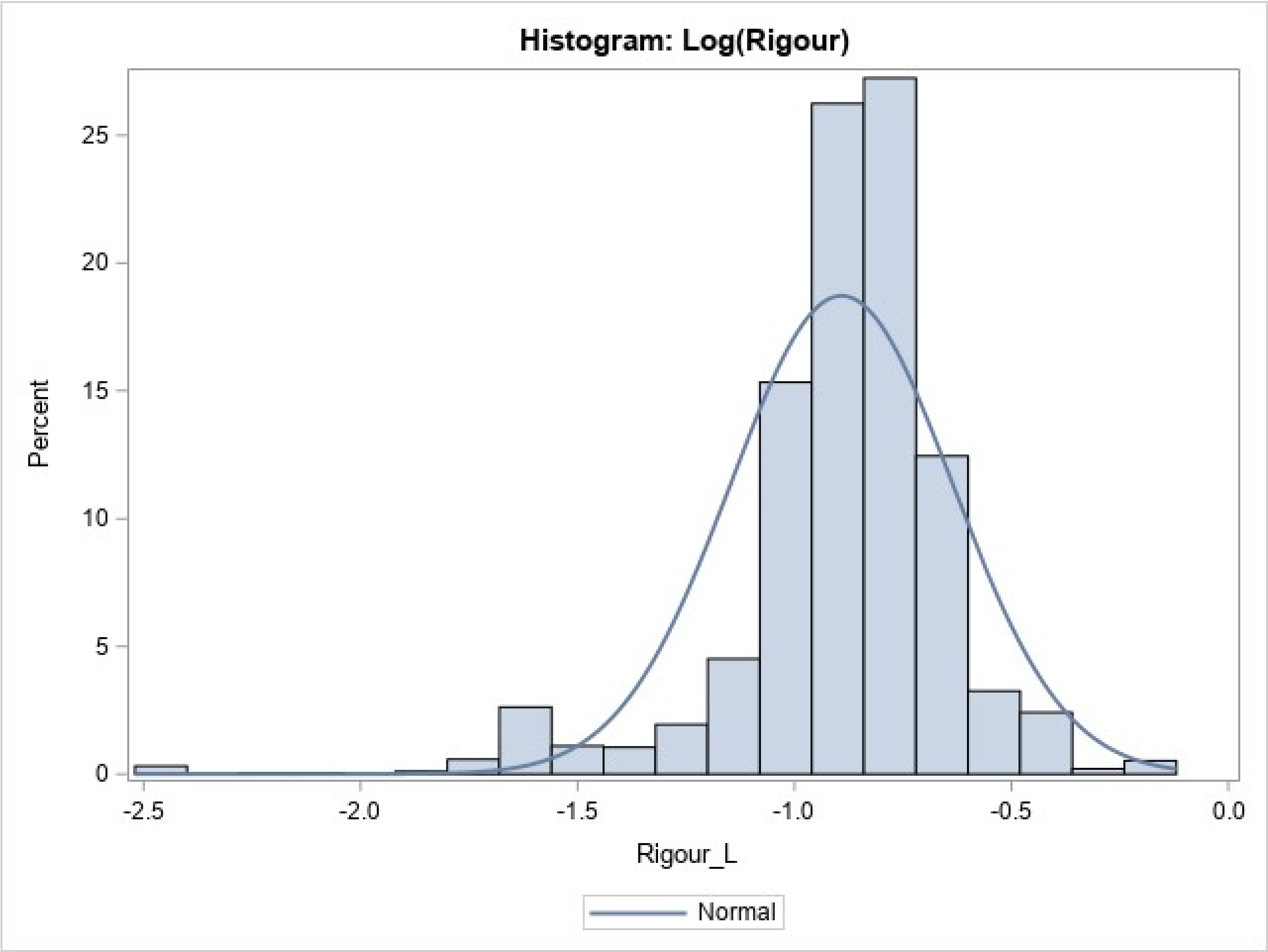
Histogram: Log(Rigour)
Percent
25
20
15
10
5
0
-2.5
-2.0
-1.5
-1.0
-0.5
0.0
Rigour_L
Normal

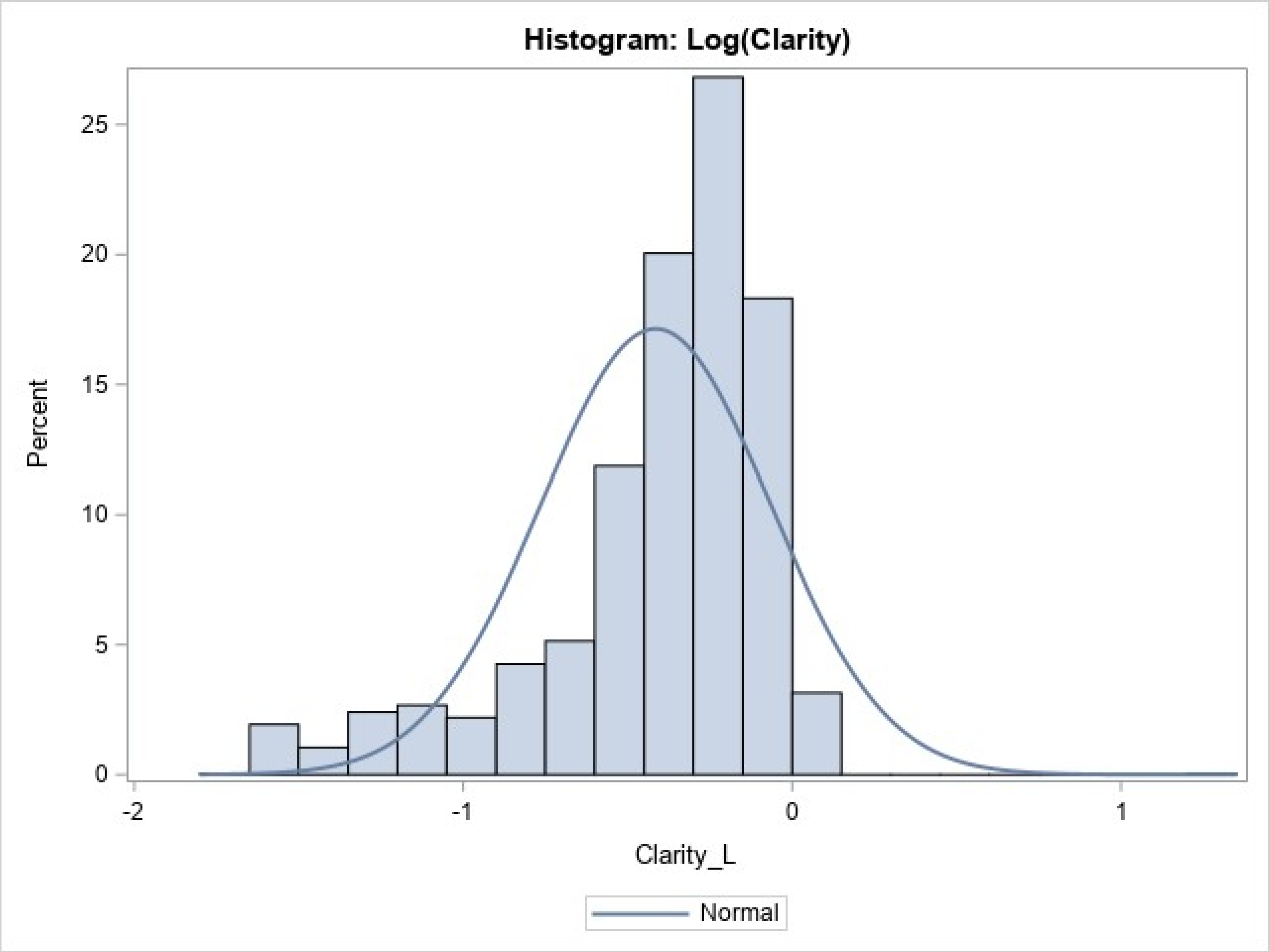
Histogram: Log(Clarity)
Percent
25
20
15
10
5
0
-2
-1
0
1
Clarity_L
Normal

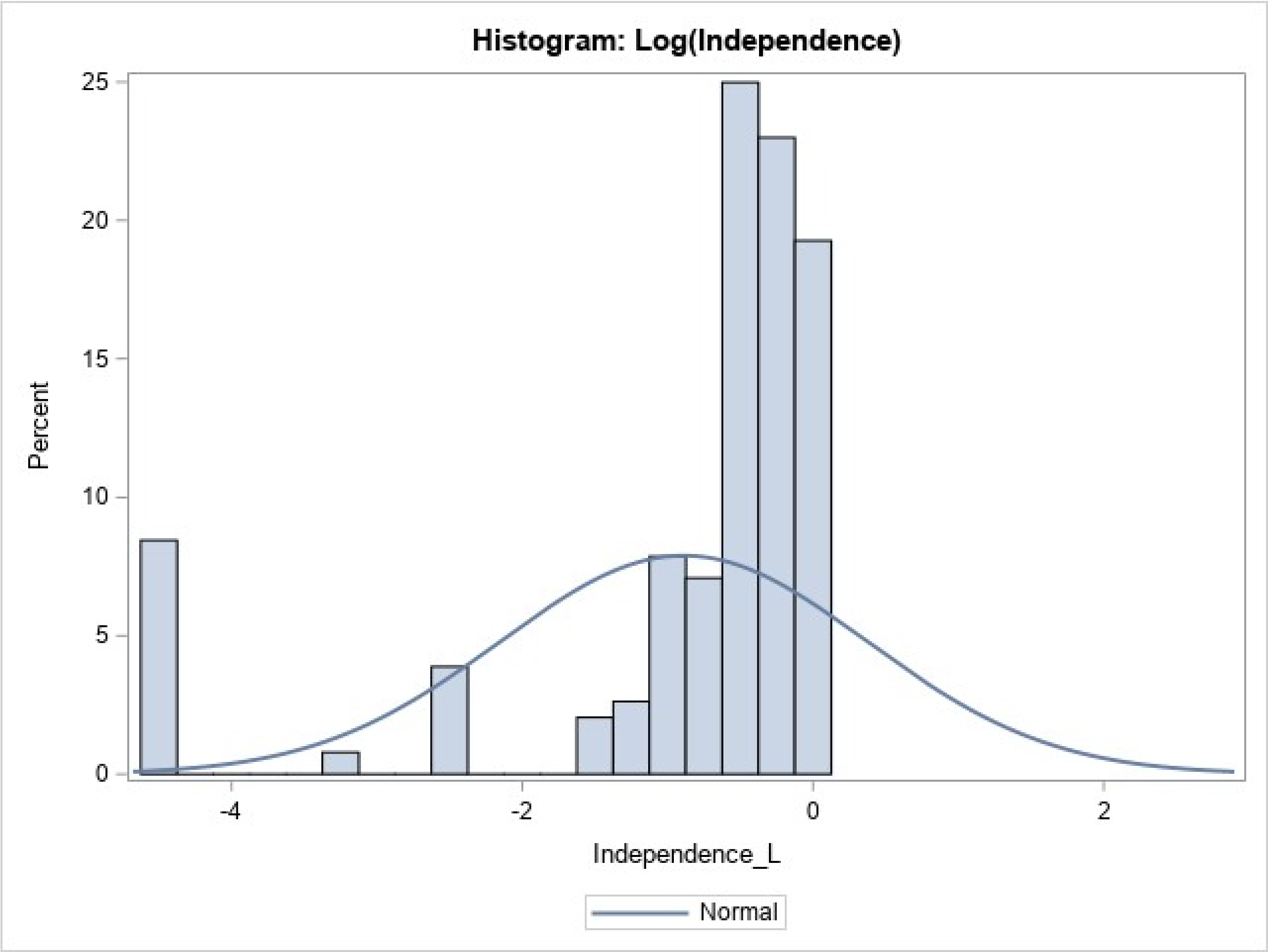
Histogram: Log(Independence)
Percent
25
20
15
10
5
0
-4
-2
0
2
Independence_L
Normal

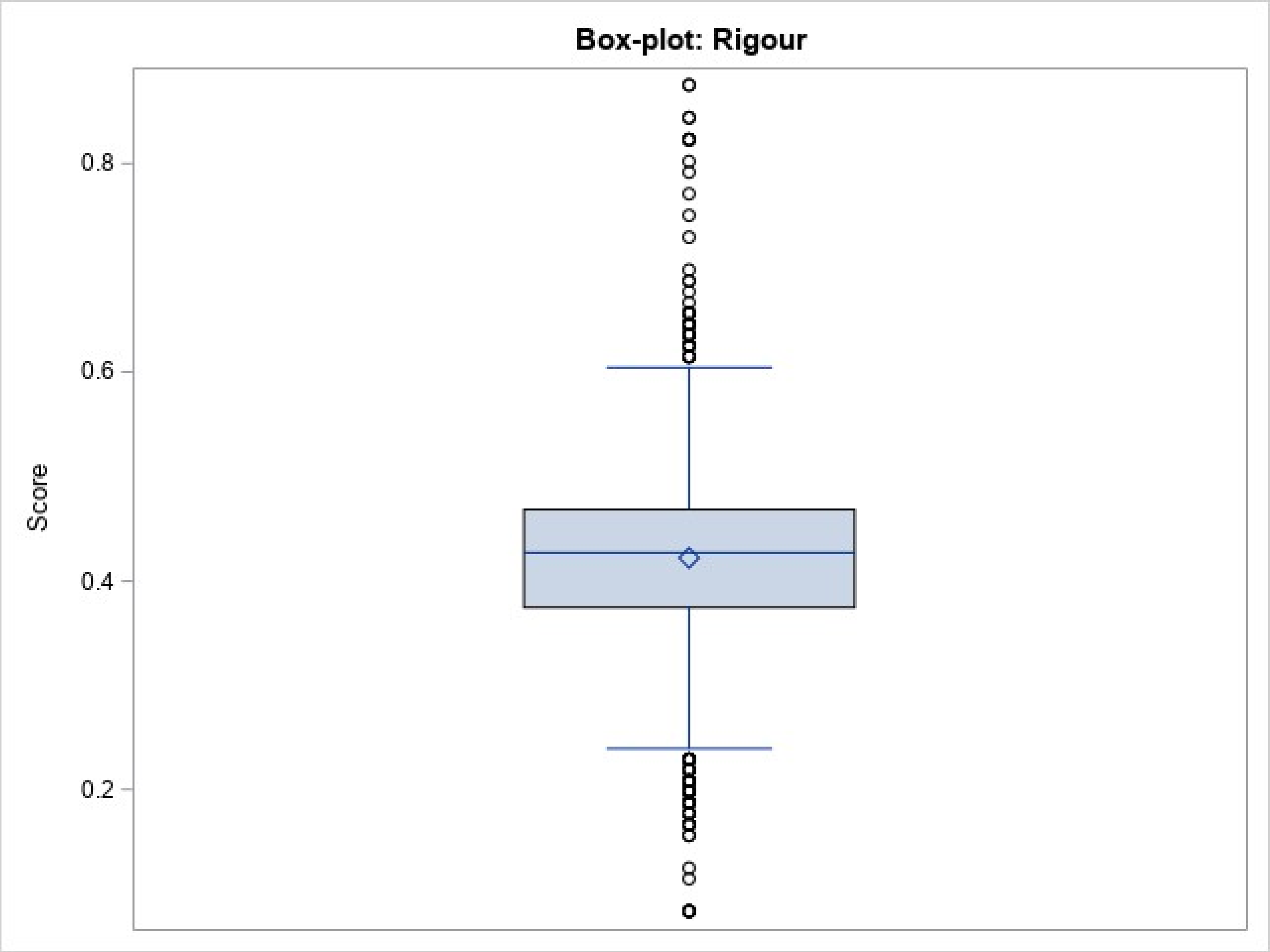
Box-plot: Rigour
Score
0.8
0.6
0.4
0.2

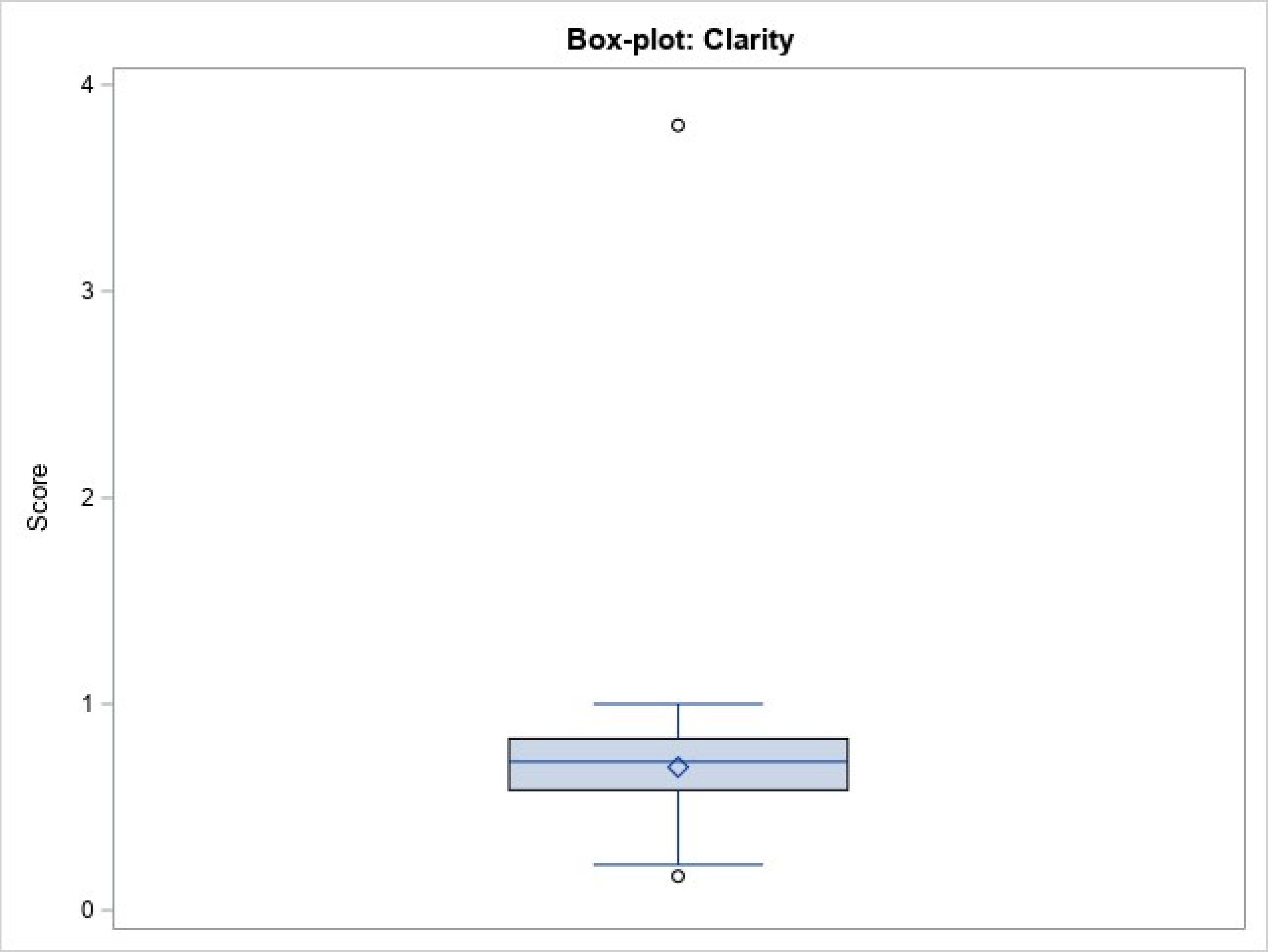
Box-plot: Clarity
Score
4
3
2
1
0

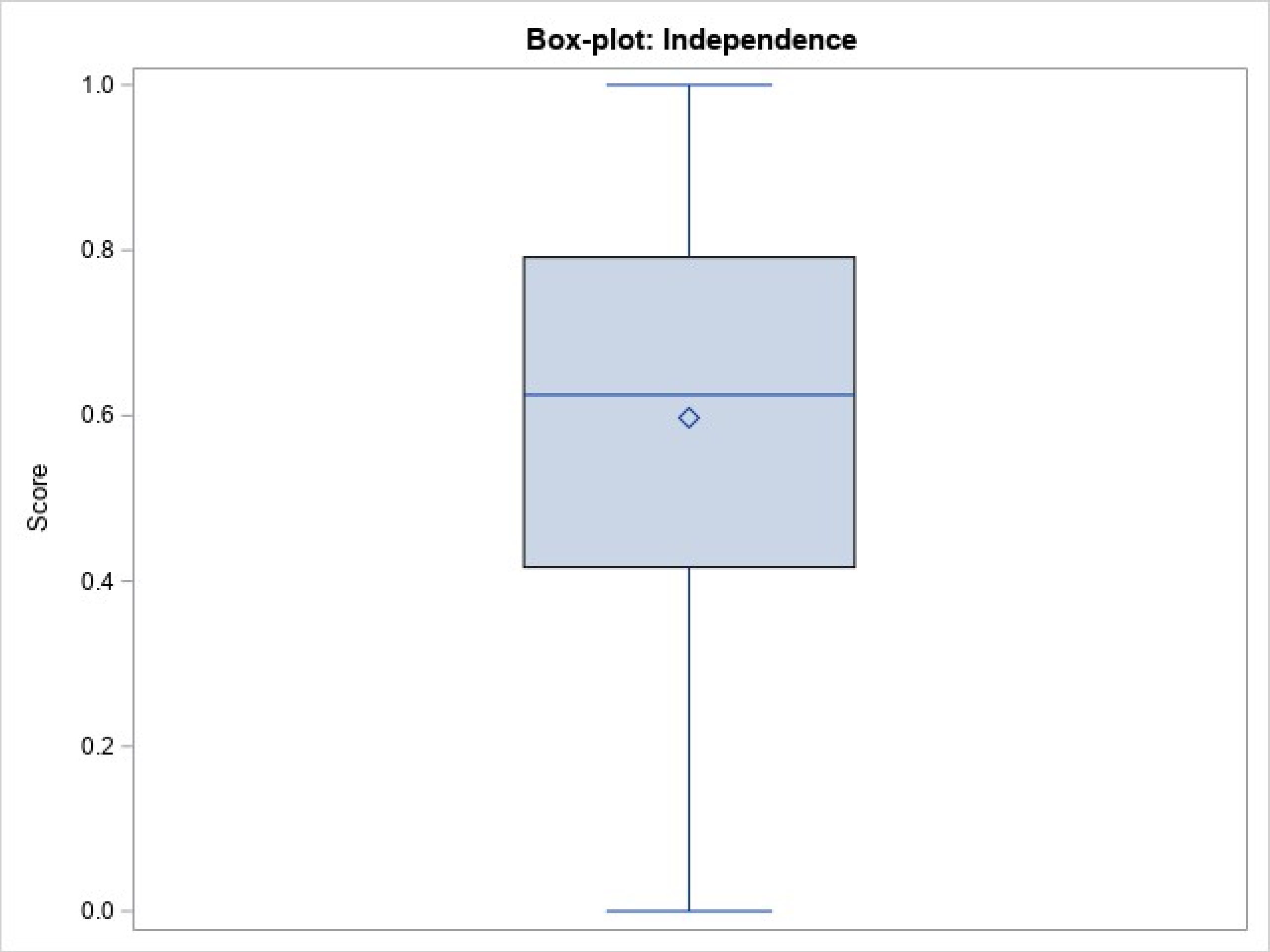
Box-plot: Independence
Score
1.0
0.8
0.6
0.4
0.2
0.0

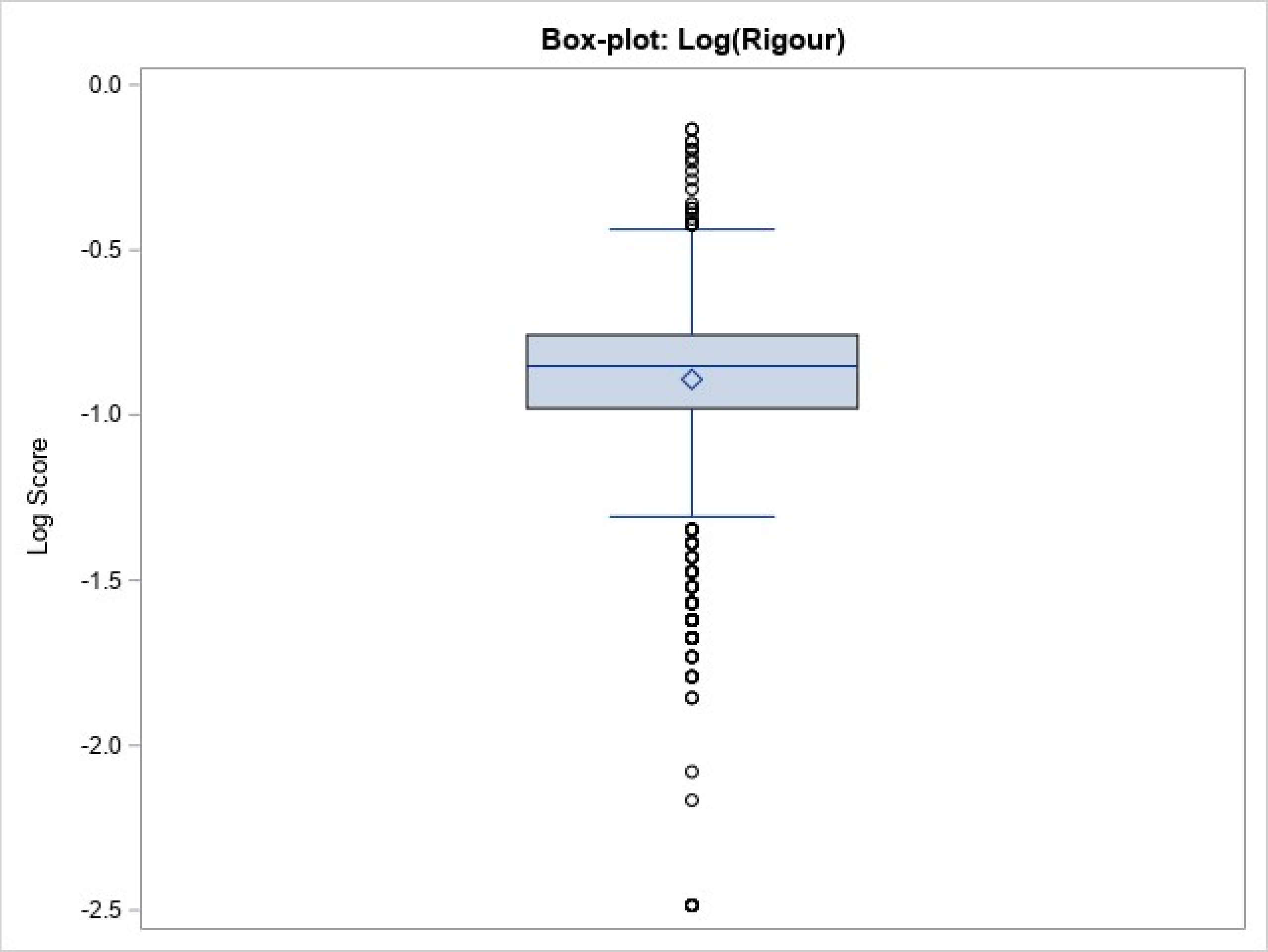
Box-plot: Log(Rigour)
Log Score
0.0
-0.5
-1.0
-1.5
-2.0
-2.5

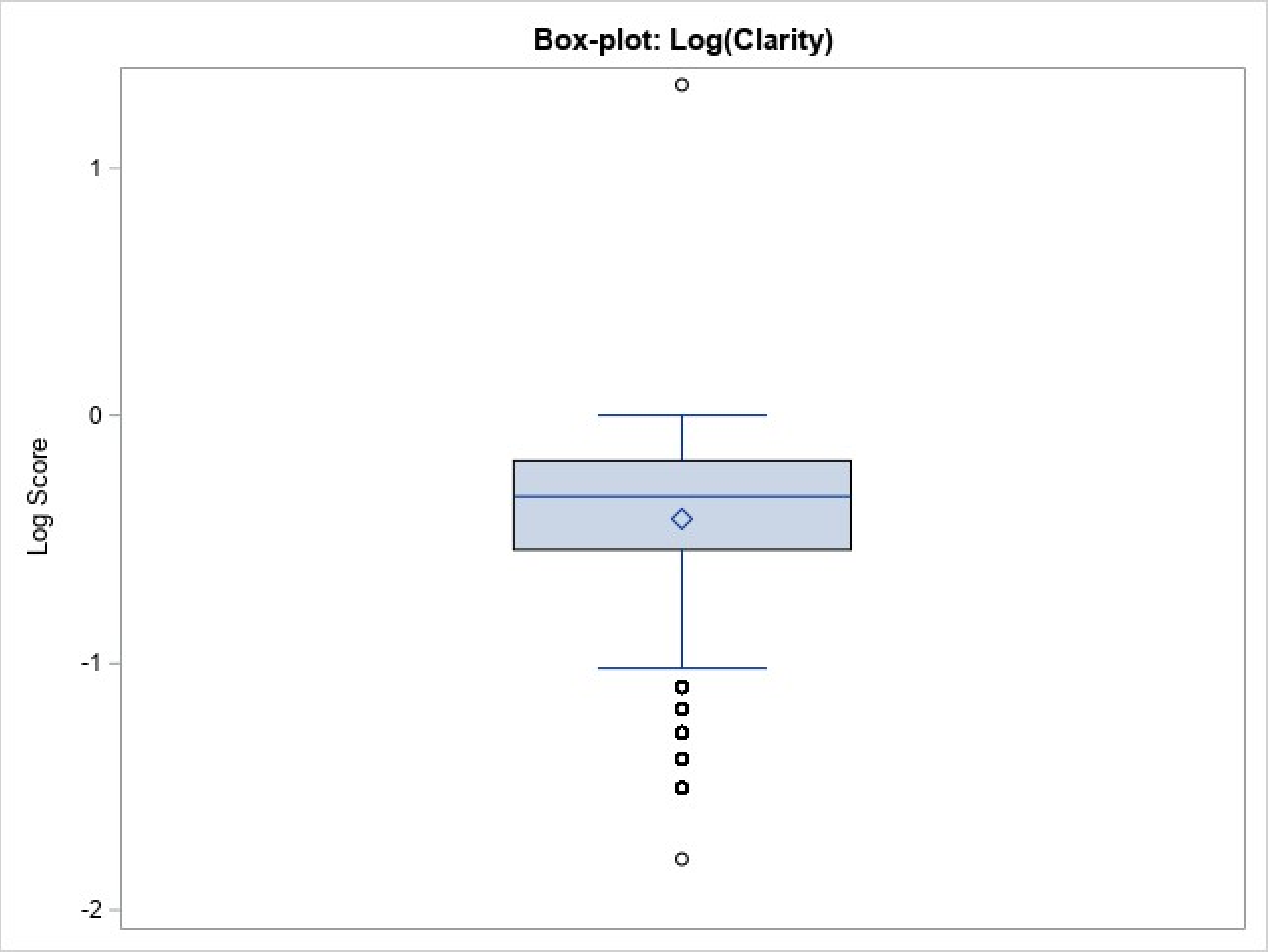
Box-plot: Log(Clarity)
Log Score
1
0
-1
-2

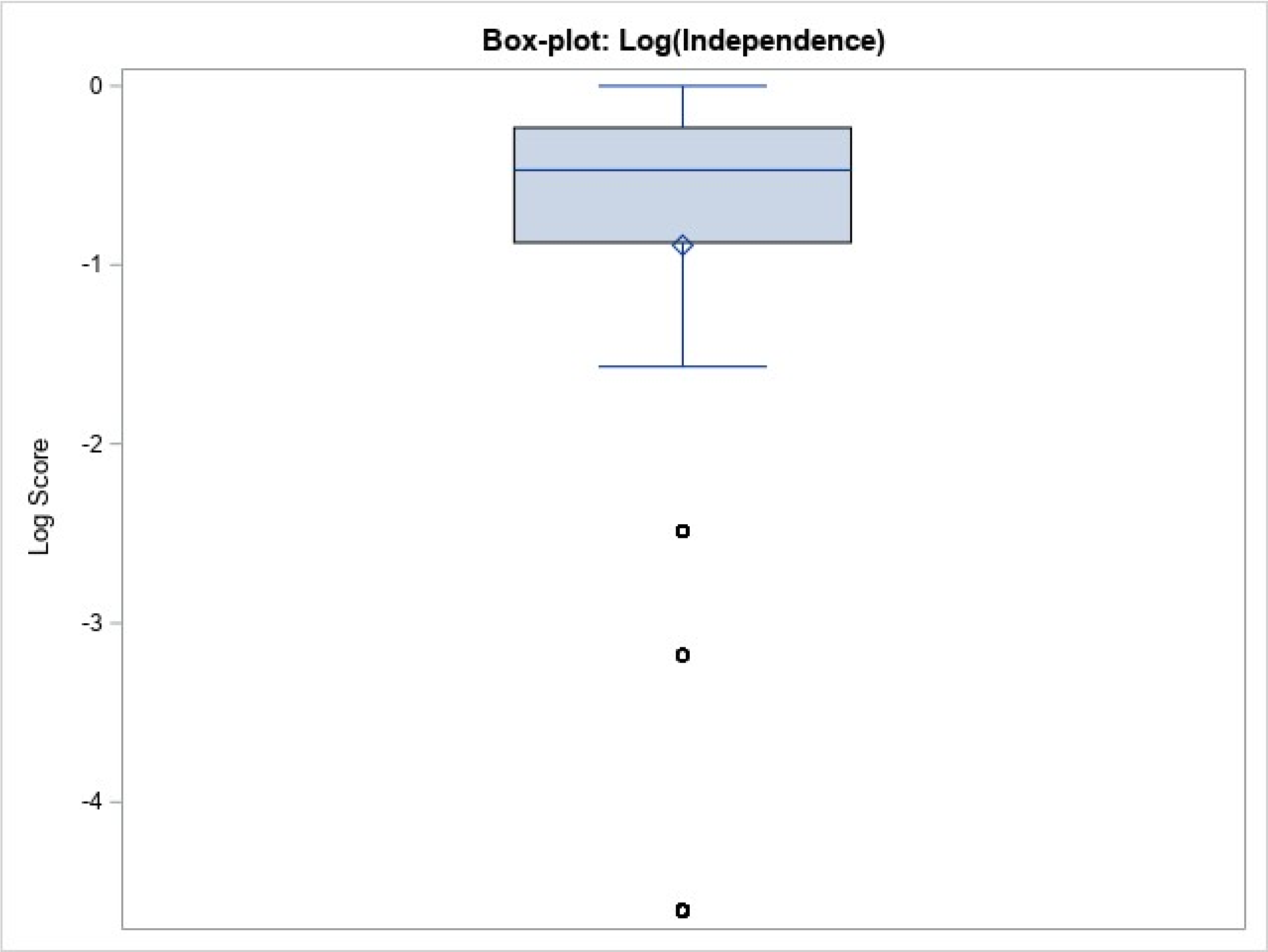
Box-plot: Log(Independence)
Log Score
0
-1
-2
-3
-4

Correlation

The CORR Procedure

3 Variables:	rigour clarity independence

Simple Statistics						
Variable	N	Mean	Std Dev	Median	Minimum	Maximum
rigour	1905	0.42207	0.09465	0.42708	0.08333	0.87500
clarity	1905	0.69577	0.20706	0.72222	0.16667	3.80556
independence	1905	0.59740	0.29565	0.62500	0	1.00000

Pearson Correlation Coefficients, N = 1905<br>Prob > |r| under H0: Rho=0			
	rigour	clarity	independence
rigour	1.00000	0.17089<br><.0001	0.25101<br><.0001
clarity	0.17089<br><.0001	1.00000	0.16299<br><.0001
independence	0.25101<br><.0001	0.16299<br><.0001	1.00000

Spearman Correlation Coefficients, N = 1905<br>Prob > |r| under H0: Rho=0			
	rigour	clarity	independence
rigour	1.00000	0.19654<br><.0001	0.16762<br><.0001
clarity	0.19654<br><.0001	1.00000	0.14919<br><.0001
independence	0.16762<br><.0001	0.14919<br><.0001	1.00000

MODEL A: ALL VARIABLES - FIXED-EFFECTS ONLY

The GLIMMIX Procedure

Model Information	
Data Set	WORK.DATAFORANALYSIS
Response Variable	differenceinrating
Response Distribution	Binary
Link Function	Logit
Variance Function	Default
Variance Matrix	Diagonal
Estimation Technique	Maximum Likelihood
Degrees of Freedom Method	Residual

Number of Observations Read	1905
Number of Observations Used	1905

Response Profile		
Ordered Value	differenceinrating	Total Frequency
1	No	1189
2	Yes	716
The GLIMMIX procedure is modeling the probability that differenceinrating='Yes'.		

Dimensions	
Columns in X	8
Columns in Z	0
Subjects (Blocks in V)	1
Max Obs per Subject	1905

Optimization Information	
Optimization Technique	Newton-Raphson
Parameters in Optimization	6
Lower Boundaries	0
Upper Boundaries	0
Fixed Effects	Not Profiled

Iteration History					
Iteration	Restarts	Evaluations	Objective Function	Change	Max Gradient
0	0	4	1104.892576	.	15.93222
1	0	3	1101.8207509	3.07182513	1.781944
2	0	3	1101.7777043	0.04304662	0.031753
3	0	3	1101.7776905	0.00001379	0.000011
4	0	2	1101.7776905	0.00000000	1.15E-12

Convergence criterion (GCONV=1E-8) satisfied.

Fit Statistics	

-2 Log Likelihood	2203.56
AIC (smaller is better)	2215.56
AICC (smaller is better)	2215.60
BIC (smaller is better)	2248.87
CAIC (smaller is better)	2254.87
HQIC (smaller is better)	2227.82
Pearson Chi-Square	2037.63
Pearson Chi-Square / DF	1.07

Parameter Estimates

Effect	FinalRecommendation	GradeSystem	Estimate	Standard Error	DF	t Value	Pr > |t|	Alpha	Lower	Upper
Intercept			2.0446	0.2968	1899	6.89	<.0001	0.05	1.4624	2.6268
rigour			-4.9378	0.6157	1899	-8.02	<.0001	0.05	-6.1453	-3.7304
clarity			-0.02185	0.2962	1899	-0.07	0.9412	0.05	-0.6028	0.5591
independence			0.04625	0.1800	1899	0.26	0.7973	0.05	-0.3068	0.3993
FinalRecommendation	Strong		-2.0266	0.1749	1899	-11.59	<.0001	0.05	-2.3695	-1.6836
FinalRecommendation	Weak		0	.	.	.	.	.	.	.
GradeSystem		GRADE	-0.4292	0.1202	1899	-3.57	0.0004	0.05	-0.6648	-0.1935
GradeSystem		Other	0	.	.	.	.	.	.	.

Type III Tests of Fixed Effects

Effect	Num DF	Den DF	F Value	Pr > F
rigour	1	1899	64.33	<.0001
clarity	1	1899	0.01	0.9412
independence	1	1899	0.07	0.7973
FinalRecommendation	1	1899	134.30	<.0001
GradeSystem	1	1899	12.76	0.0004

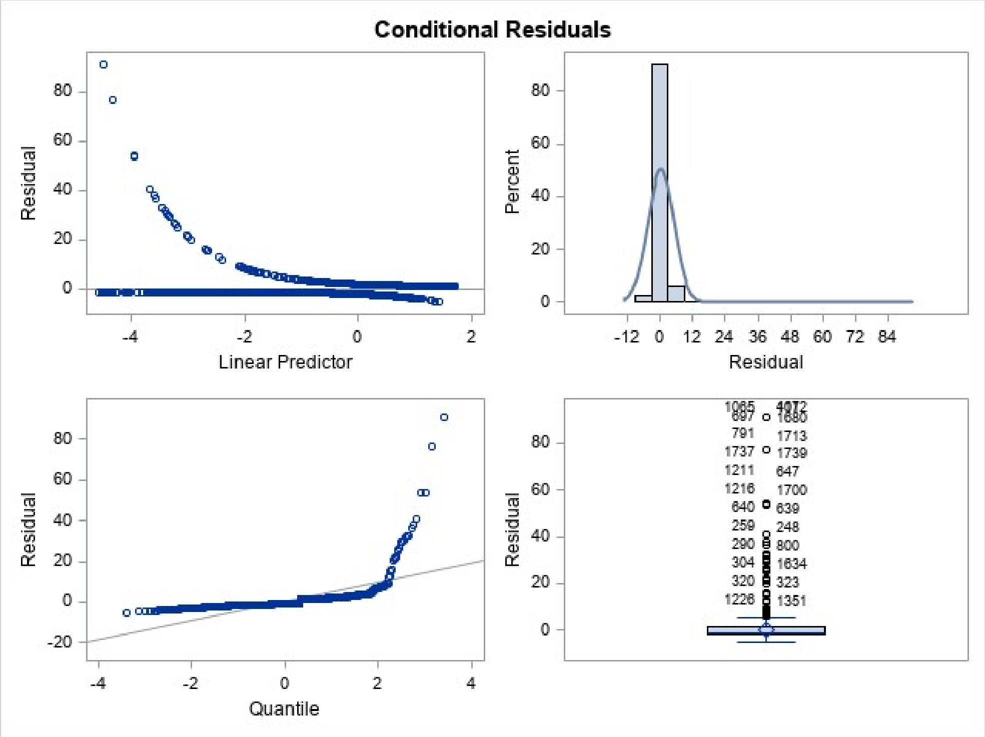
Conditional Residuals
Residual
Linear Predictor
Percent
Residual
Residual
Quantile
Residual

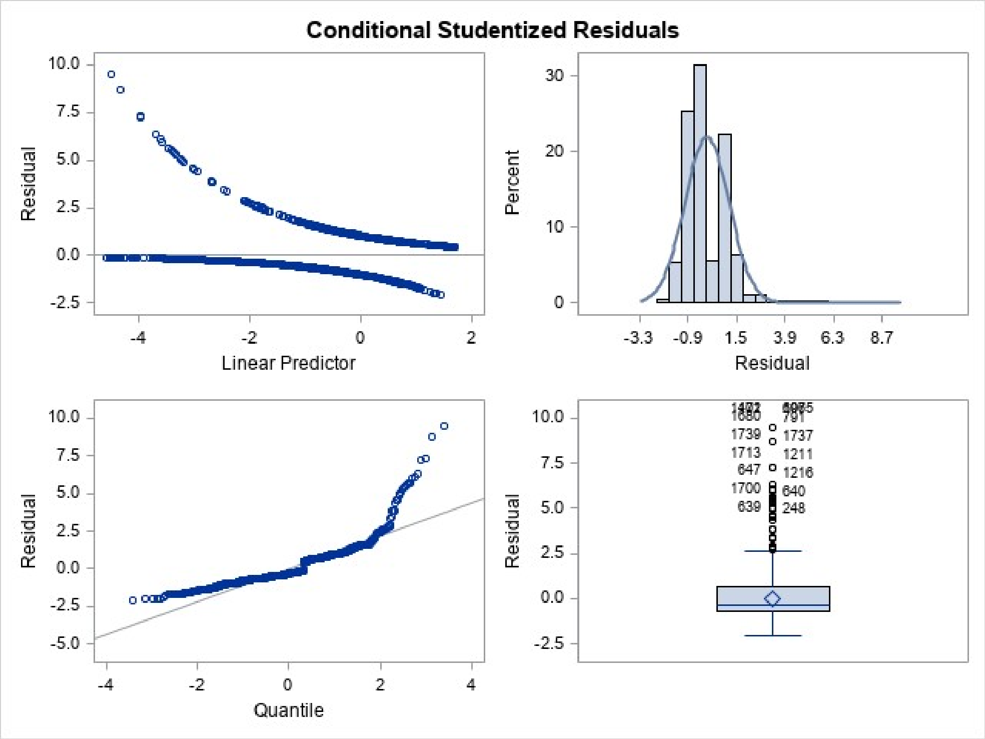
Conditional Studentized Residuals
Residual
Linear Predictor
Percent
Residual
Residual
Quantile
Residual

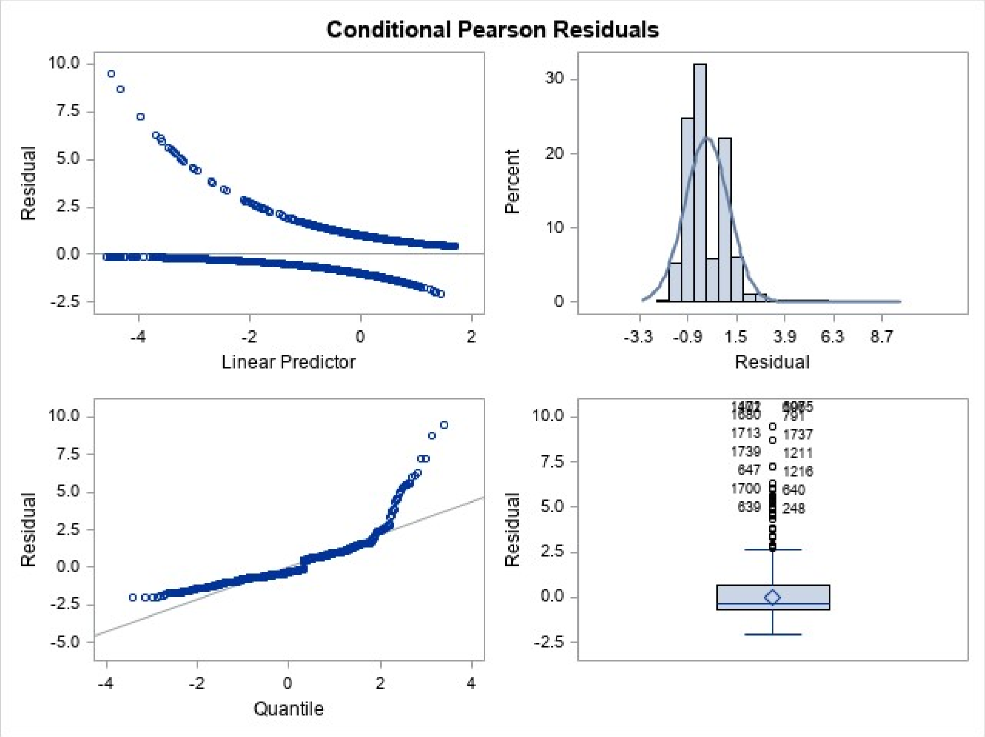
Conditional Pearson Residuals
Residual
Linear Predictor
Percent
Residual
Residual
Quantile
Residual
1713
1737
1739
1211
647
1216
1700
640
639
248

MODEL A: ALL VARIABLES - FIXED-EFFECTS ONLY

The LOGISTIC Procedure

Model Information	
Data Set	WORK.GLMMOUT
Response Variable	differenceinrating
Number of Response Levels	2
Model	binary logit
Optimization Technique	Fisher's scoring

Number of Observations Read	1905
Number of Observations Used	1905

Response Profile		
Ordered Value	differenceinrating	Total Frequency
1	No	1189
2	Yes	716

Probability modeled is differenceinrating='Yes'.

Score Test for Global Null Hypothesis		
Chi-Square	DF	Pr > ChiSq
0.0000	0	<.0001

ROC Model: GLIMMIX model

ROC Model Information		
ROC Contrast Coefficients	predprob	Mu

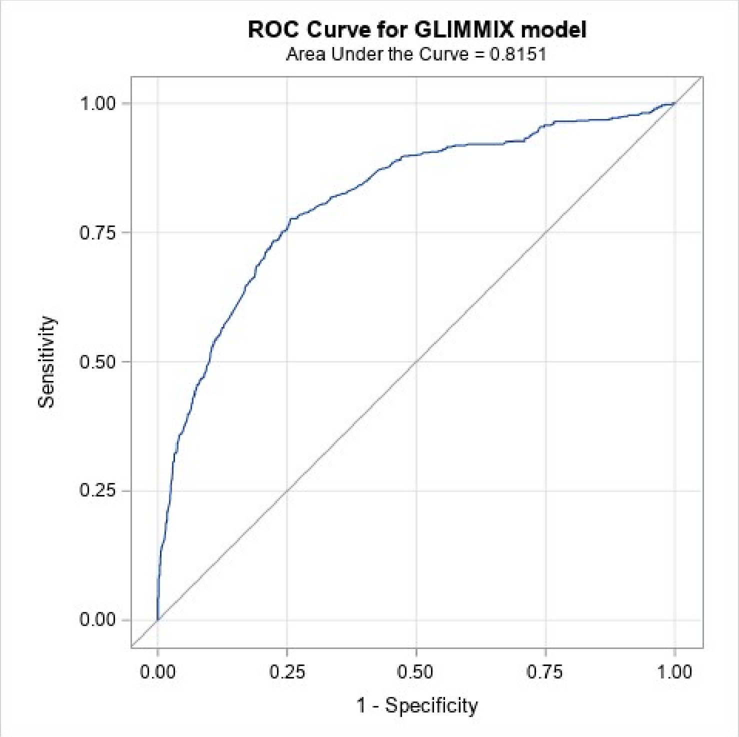

ROC Association Statistics							
	Mann-Whitney						
ROC Model	Area	Standard Error	95% Wald Confidence Limits		Somers' D	Gamma	Tau-a
GLIMMIX model	0.7315	0.0116	0.7087	0.7543	0.4630	0.4633	0.2173

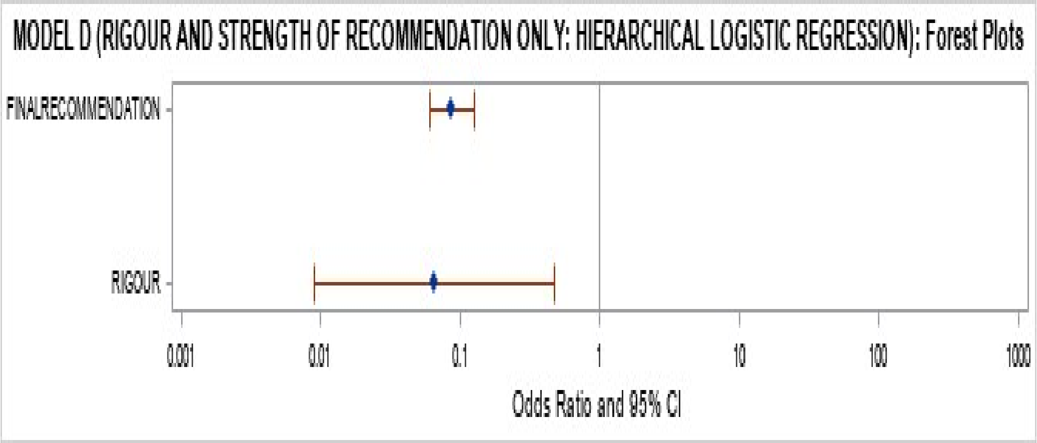
MODEL D (RIGOUR AND STRENGTH OF RECOMMENDATION ONLY: HIERARCHICAL LOGISTIC REGRESSION): Forest Plots
FINALRECOMMENDATION
RIGOUR
0.001
0.01
0.1
1
10
100
1000
Odds Ratio and 95% CI

MODEL B: ALL VARIABLES - HIERARCHICAL LOGISTIC REGRESSION

The GLIMMIX Procedure

Model Information	
Data Set	WORK.DATAFORANALYSIS
Response Variable	differenceinrating
Response Distribution	Binary
Link Function	Logit
Variance Function	Default
Variance Matrix Blocked By	guidelineid
Estimation Technique	Maximum Likelihood
Likelihood Approximation	Laplace
Degrees of Freedom Method	Containment

Number of Observations Read	1905
Number of Observations Used	1905

Response Profile		
Ordered Value	differenceinrating	Total Frequency
1	No	1189
2	Yes	716

The GLIMMIX procedure is modeling the probability that differenceinrating='Yes'.

Dimensions	
G-side Cov. Parameters	1
Columns in X	8
Columns in Z per Subject	1
Subjects (Blocks in V)	63
Max Obs per Subject	152

Optimization Information	
Optimization Technique	Dual Quasi-Newton
Parameters in Optimization	7
Lower Boundaries	1
Upper Boundaries	0
Fixed Effects	Not Profiled
Starting From	GLM estimates

Iteration History

Iteration	Restarts	Evaluations	Objective Function	Change	Max Gradient
0	0	4	2093.6422598	.	25.78955
1	0	3	2088.0179762	5.62428358	14.94287
2	0	3	2086.2142035	1.80377267	4.82614
3	0	3	2085.1605917	1.05361188	8.809345

4	0	4	2082.1077374	3.05285429	4.164786
5	0	3	2080.3820282	1.72570916	3.919568
6	0	2	2079.7746778	0.60735046	3.359583
7	0	2	2079.0180844	0.75659335	0.230738
8	0	3	2079.0150412	0.00304317	0.03542
9	0	3	2079.0150022	0.00003906	0.005818
10	0	3	2079.0150017	0.00000050	0.000342

Convergence criterion (GCONV=1E-8) satisfied.

Fit Statistics	
-2 Log Likelihood	2079.02
AIC (smaller is better)	2093.02
AICC (smaller is better)	2093.07
BIC (smaller is better)	2108.02
CAIC (smaller is better)	2115.02
HQIC (smaller is better)	2098.92

Fit Statistics for Conditional Distribution	
-2 log L(differenceinrating | r. effects)	1958.21
Pearson Chi-Square	2250.08
Pearson Chi-Square / DF	1.18

Covariance Parameter Estimates

Cov Parm	Subject	Estimate	Standard Error	Z Value	Pr > Z
Intercept	guidelineid	0.6392	0.1919	3.33	0.0004

Solutions for Fixed Effects

Effect	FinalRecommendation	GradeSystem	Estimate	Standard Error	DF	t Value	Pr > |t|	Alpha	Lower	Upper
Intercept			1.1977	0.5304	62	2.26	0.0275	0.05	0.1375	2.2579
rigour			-2.2346	1.0863	1837	-2.06	0.0398	0.05	-4.3651	-0.1041
clarity			0.1247	0.5736	1837	0.22	0.8279	0.05	-1.0003	1.2498
independence			-0.3977	0.4239	1837	-0.94	0.3483	0.05	-1.2292	0.4338
FinalRecommendation	Strong		-2.4668	0.1904	1837	-12.96	<.0001	0.05	-2.8402	-2.0933
FinalRecommendation	Weak		0	.	.	.	.	.	.	.
GradeSystem		GRADE	-0.4153	0.2689	1837	-1.54	0.1227	0.05	-0.9426	0.1121
GradeSystem		Other	0	.	.	.	.	.	.	.

Type III Tests of Fixed Effects

Effect	Num DF	Den DF	F Value	Pr > F
rigour	1	1837	4.23	0.0398
clarity	1	1837	0.05	0.8279
independence	1	1837	0.88	0.3483
FinalRecommendation	1	1837	167.84	<.0001
GradeSystem	1	1837	2.39	0.1227

Solution for Random Effects

Effect	Subject	Estimate	Std Err Pred	DF	t Value	Pr > |t|	Alpha	Lower	Upper
Intercept	guidelineid 1	-0.5182	0.6163	1837	-0.84	0.4005	0.05	-1.7269	0.6905
Intercept	guidelineid 2	-0.8973	0.2855	1837	-3.14	0.0017	0.05	-1.4571	-0.3374
Intercept	guidelineid 3	1.4610	0.5051	1837	2.89	0.0039	0.05	0.4703	2.4518
Intercept	guidelineid 4	0.4728	0.3541	1837	1.34	0.1819	0.05	-0.2216	1.1672
Intercept	guidelineid 5	-0.2398	0.5237	1837	-0.46	0.6471	0.05	-1.2668	0.7873
Intercept	guidelineid 6	-0.5266	0.6921	1837	-0.76	0.4468	0.05	-1.8840	0.8307
Intercept	guidelineid 7	-0.2427	0.3220	1837	-0.75	0.4512	0.05	-0.8742	0.3889
Intercept	guidelineid 8	0.1341	0.6214	1837	0.22	0.8291	0.05	-1.0846	1.3529
Intercept	guidelineid 9	0.5978	0.4319	1837	1.38	0.1665	0.05	-0.2494	1.4449
Intercept	guidelineid 10	0.6279	0.3921	1837	1.60	0.1094	0.05	-0.1411	1.3969
Intercept	guidelineid 11	-0.5360	0.6000	1837	-0.89	0.3719	0.05	-1.7128	0.6408
Intercept	guidelineid 12	-1.0347	0.4563	1837	-2.27	0.0235	0.05	-1.9297	-0.1397
Intercept	guidelineid 13	-0.1763	0.7527	1837	-0.23	0.8148	0.05	-1.6525	1.2998
Intercept	guidelineid 14	-0.4450	0.4532	1837	-0.98	0.3262	0.05	-1.3337	0.4438
Intercept	guidelineid 15	-0.3385	0.6236	1837	-0.54	0.5873	0.05	-1.5616	0.8846
Intercept	guidelineid 16	0.8980	0.4218	1837	2.13	0.0334	0.05	0.07066	1.7253
Intercept	guidelineid 17	0.5613	0.3826	1837	1.47	0.1425	0.05	-0.1890	1.3117
Intercept	guidelineid 18	0.3897	0.5048	1837	0.77	0.4403	0.05	-0.6005	1.3798
Intercept	guidelineid 19	0.6947	0.3982	1837	1.74	0.0812	0.05	-0.08631	1.4758
Intercept	guidelineid 20	1.0527	0.3774	1837	2.79	0.0053	0.05	0.3125	1.7929
Intercept	guidelineid 21	0.1898	0.4242	1837	0.45	0.6545	0.05	-0.6421	1.0218
Intercept	guidelineid 22	-0.4660	0.3971	1837	-1.17	0.2407	0.05	-1.2449	0.3128
Intercept	guidelineid 23	-0.1529	0.5284	1837	-0.29	0.7724	0.05	-1.1892	0.8835
Intercept	guidelineid 24	-0.9686	0.4522	1837	-2.14	0.0323	0.05	-1.8554	-0.08180
Intercept	guidelineid 25	1.1568	0.3938	1837	2.94	0.0033	0.05	0.3844	1.9291
Intercept	guidelineid 26	-1.2453	0.4985	1837	-2.50	0.0126	0.05	-2.2229	-0.2676
Intercept	guidelineid 27	-0.3835	0.4158	1837	-0.92	0.3564	0.05	-1.1990	0.4319
Intercept	guidelineid 28	0.8511	0.5039	1837	1.69	0.0914	0.05	-0.1372	1.8395
Intercept	guidelineid 29	-0.5977	0.6077	1837	-0.98	0.3255	0.05	-1.7896	0.5942
Intercept	guidelineid 30	-0.4804	0.6937	1837	-0.69	0.4887	0.05	-1.8409	0.8802
Intercept	guidelineid 31	0.4561	0.2905	1837	1.57	0.1166	0.05	-0.1136	1.0257
Intercept	guidelineid 32	-0.5330	0.3224	1837	-1.65	0.0985	0.05	-1.1654	0.09936
Intercept	guidelineid 33	-0.06339	0.4894	1837	-0.13	0.8970	0.05	-1.0233	0.8965
Intercept	guidelineid 34	0.5762	0.6268	1837	0.92	0.3581	0.05	-0.6532	1.8056
Intercept	guidelineid 35	-1.4941	0.3135	1837	-4.77	<.0001	0.05	-2.1089	-0.8793
Intercept	guidelineid 36	-0.2863	0.4553	1837	-0.63	0.5295	0.05	-1.1794	0.6067
Intercept	guidelineid 37	0.2954	0.4784	1837	0.62	0.5370	0.05	-0.6429	1.2338
Intercept	guidelineid 38	-0.2103	0.4540	1837	-0.46	0.6433	0.05	-1.1007	0.6801
Intercept	guidelineid 39	0.2396	0.4054	1837	0.59	0.5545	0.05	-0.5554	1.0347
Intercept	guidelineid 40	0.5201	0.4553	1837	1.14	0.2534	0.05	-0.3728	1.4130
Intercept	guidelineid 41	-0.1413	0.3398	1837	-0.42	0.6777	0.05	-0.8077	0.5252
Intercept	guidelineid 42	1.1279	0.4109	1837	2.74	0.0061	0.05	0.3220	1.9339
Intercept	guidelineid 43	-0.6850	0.5012	1837	-1.37	0.1719	0.05	-1.6680	0.2980
Intercept	guidelineid 44	-0.8514	0.4936	1837	-1.73	0.0847	0.05	-1.8194	0.1166
Intercept									

	guidelineid 45	0.06402	0.4001	1837	0.16	0.8729	0.05	-0.7207	0.8488
**Intercept**	guidelineid 46	0.3345	0.5268	1837	0.63	0.5255	0.05	-0.6987	1.3677
**Intercept**	guidelineid 47	0.8285	0.5182	1837	1.60	0.1100	0.05	-0.1878	1.8449
**Intercept**	guidelineid 48	-0.07836	0.3536	1837	-0.22	0.8246	0.05	-0.7718	0.6151
**Intercept**	guidelineid 49	-0.1332	0.4150	1837	-0.32	0.7483	0.05	-0.9472	0.6808
**Intercept**	guidelineid 50	-0.6525	0.3241	1837	-2.01	0.0442	0.05	-1.2881	-0.01696
**Intercept**	guidelineid 51	0.6857	0.3812	1837	1.80	0.0722	0.05	-0.06193	1.4333
**Intercept**	guidelineid 52	-0.5570	0.5703	1837	-0.98	0.3289	0.05	-1.6755	0.5615
**Intercept**	guidelineid 53	-0.3607	0.5760	1837	-0.63	0.5312	0.05	-1.4904	0.7689
**Intercept**	guidelineid 54	-1.0842	0.5654	1837	-1.92	0.0553	0.05	-2.1930	0.02457
**Intercept**	guidelineid 55	-0.06783	0.6627	1837	-0.10	0.9185	0.05	-1.3675	1.2318
**Intercept**	guidelineid 56	0.7121	0.5875	1837	1.21	0.2256	0.05	-0.4401	1.8643
**Intercept**	guidelineid 57	-0.02418	0.6697	1837	-0.04	0.9712	0.05	-1.3376	1.2893
**Intercept**	guidelineid 58	0.4243	0.5866	1837	0.72	0.4696	0.05	-0.7261	1.5747
**Intercept**	guidelineid 59	0.2991	0.5246	1837	0.57	0.5686	0.05	-0.7298	1.3280
**Intercept**	guidelineid 60	0.6136	0.6106	1837	1.00	0.3151	0.05	-0.5840	1.8112
**Intercept**	guidelineid 61	0.5168	0.5203	1837	0.99	0.3207	0.05	-0.5037	1.5374
**Intercept**	guidelineid 62	-0.02704	0.5809	1837	-0.05	0.9629	0.05	-1.1664	1.1123
**Intercept**	guidelineid 63	0.2280	0.6256	1837	0.36	0.7155	0.05	-0.9988	1.4549

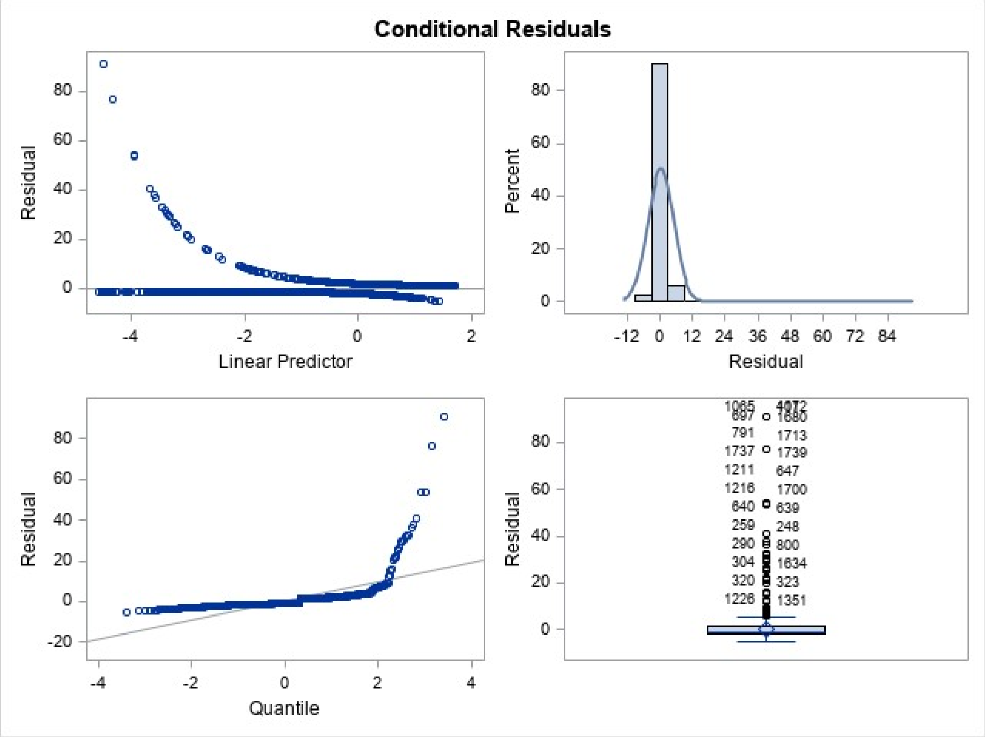

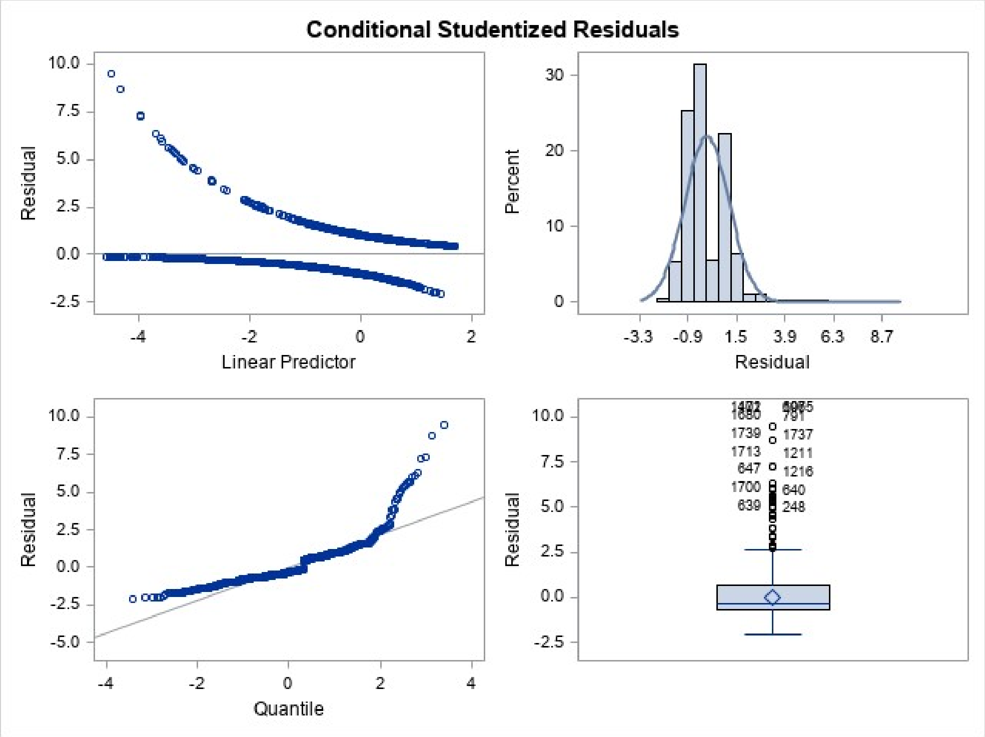
Conditional Studentized Residuals
Residual
Linear Predictor
Percent
Residual
Quantile

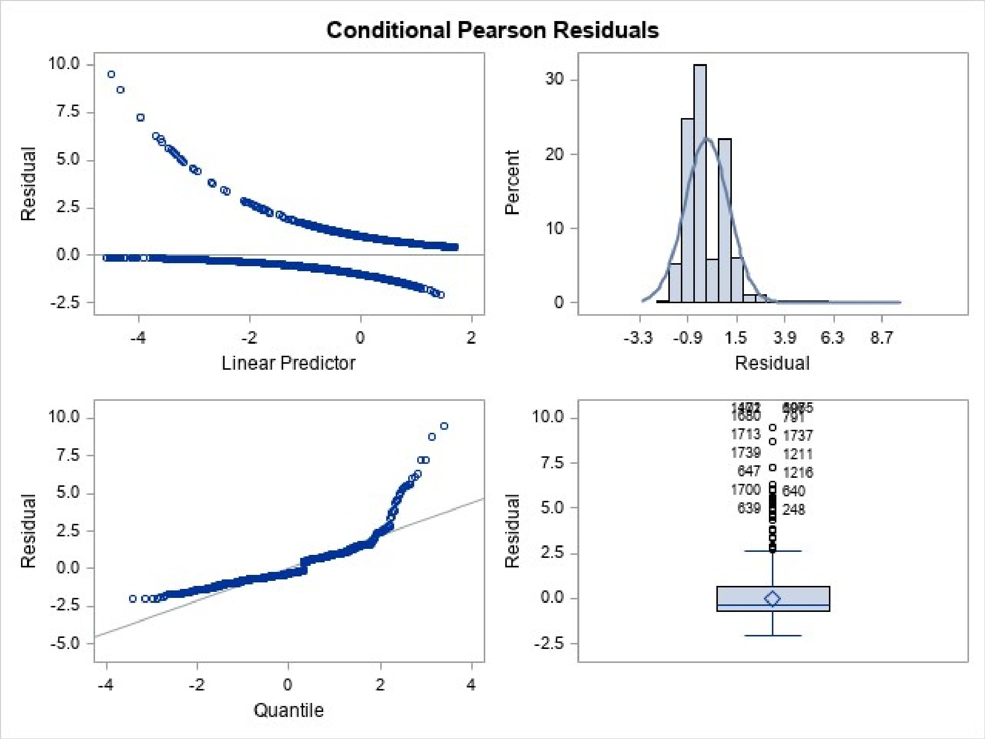
Conditional Pearson Residuals
Residual
Linear Predictor
Percent
Residual
Quantile

MODEL B: ALL VARIABLES - HIERARCHICAL LOGISTIC REGRESSION

The LOGISTIC Procedure

Model Information	
Data Set	WORK.GLMMOUT
Response Variable	differenceinrating
Number of Response Levels	2
Model	binary logit
Optimization Technique	Fisher's scoring

Number of Observations Read	1905
Number of Observations Used	1905

Response Profile		
Ordered Value	differenceinrating	Total Frequency
1	No	1189
2	Yes	716

Probability modeled is differenceinrating='Yes'.

Score Test for Global Null Hypothesis		
Chi-Square	DF	Pr > ChiSq
0.0000	0	<.0001

ROC Model: GLIMMIX model

ROC Model Information		
ROC Contrast Coefficients	predprob	Mu

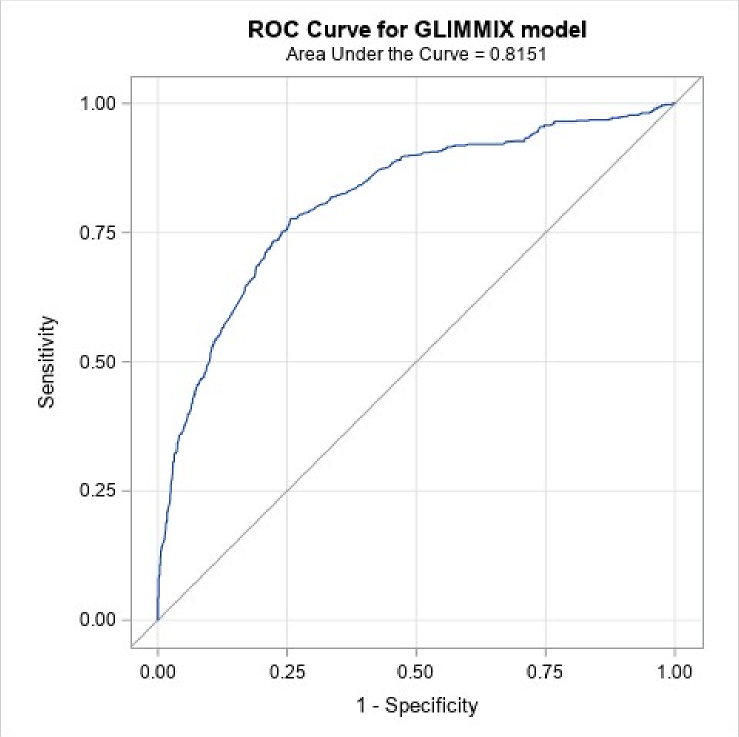

ROC Association Statistics							
	Mann-Whitney						
ROC Model	Area	Standard Error	95% Wald Confidence Limits		Somers' D	Gamma	Tau-a
GLIMMIX model	0.8167	0.0103	0.7965	0.8368	0.6334	0.6338	0.2973

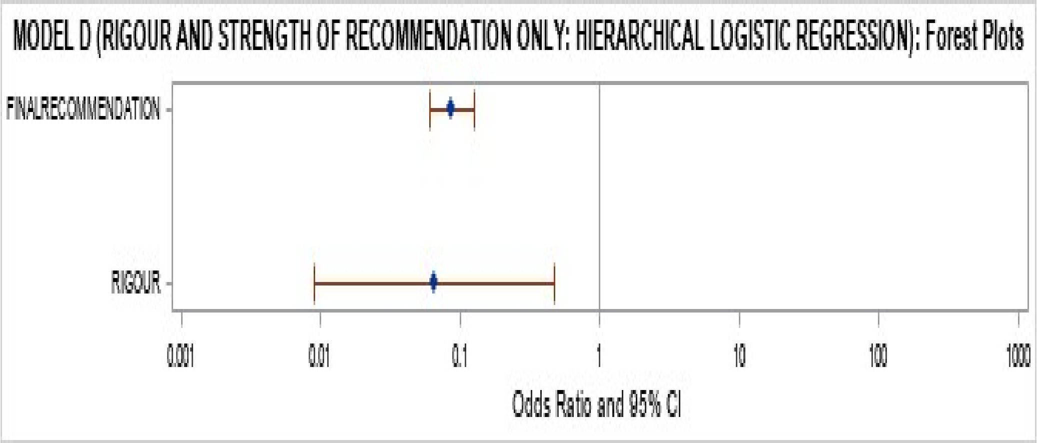
MODEL D (RIGOUR AND STRENGTH OF RECOMMENDATION ONLY: HIERARCHICAL LOGISTIC REGRESSION): Forest Plots
FINALRECOMMENDATION
RIGOUR
0.001
0.01
0.1
1
10
100
1000
Odds Ratio and 95% CI

MODEL C: RIGOUR AND STRENGTH OF RECOMMENDATION ONLY - FIXED-EFFECTS ONLY

The GLIMMIX Procedure

Model Information	
Data Set	WORK.DATAFORANALYSIS
Response Variable	differenceinrating
Response Distribution	Binary
Link Function	Logit
Variance Function	Default
Variance Matrix	Diagonal
Estimation Technique	Maximum Likelihood
Degrees of Freedom Method	Residual

Number of Observations Read	1905
Number of Observations Used	1905

Response Profile		
Ordered Value	differenceinrating	Total Frequency
1	No	1189
2	Yes	716

The GLIMMIX procedure is modeling the probability that differenceinrating='Yes'.

Dimensions	
Columns in X	4
Columns in Z	0
Subjects (Blocks in V)	1
Max Obs per Subject	1905

Optimization Information	
Optimization Technique	Newton-Raphson
Parameters in Optimization	3
Lower Boundaries	0
Upper Boundaries	0
Fixed Effects	Not Profiled

Iteration History

Iteration	Restarts	Evaluations	Objective Function	Change	Max Gradient
0	0	4	1112.9853788	.	14.94884
1	0	3	1110.328324	2.65705477	1.589516
2	0	3	1110.2945728	0.03375121	0.02537
3	0	3	1110.2945641	0.00000872	6.751E-6

Convergence criterion (ABSGCONV=0.00001) satisfied.

Fit Statistics

-2 Log Likelihood	2220.59
AIC (smaller is better)	2226.59
AICC (smaller is better)	2226.60
BIC (smaller is better)	2243.25
CAIC (smaller is better)	2246.25
HQIC (smaller is better)	2232.72
Pearson Chi-Square	1997.93
Pearson Chi-Square / DF	1.05

Parameter Estimates

Effect	FinalRecommendation	Estimate	Standard Error	DF	t Value	Pr > |t|	Alpha	Lower	Upper
Intercept		1.9620	0.2504	1902	7.83	<.0001	0.05	1.4708	2.4531
rigour		-5.1352	0.5921	1902	-8.67	<.0001	0.05	-6.2965	-3.9739
FinalRecommendation	Strong	-2.0199	0.1729	1902	-11.69	<.0001	0.05	-2.3589	-1.6809
FinalRecommendation	Weak	0	.	.	.	.	.	.	.

Type III Tests of Fixed Effects

Effect	Num DF	Den DF	F Value	Pr > F
rigour	1	1902	75.21	<.0001
FinalRecommendation	1	1902	136.56	<.0001

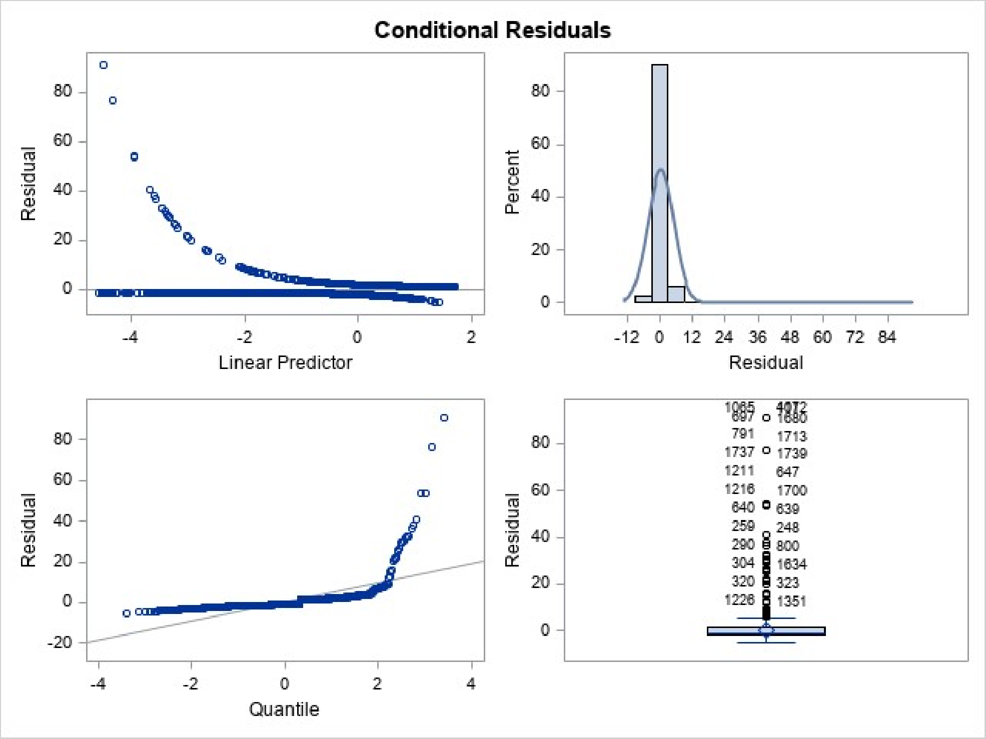

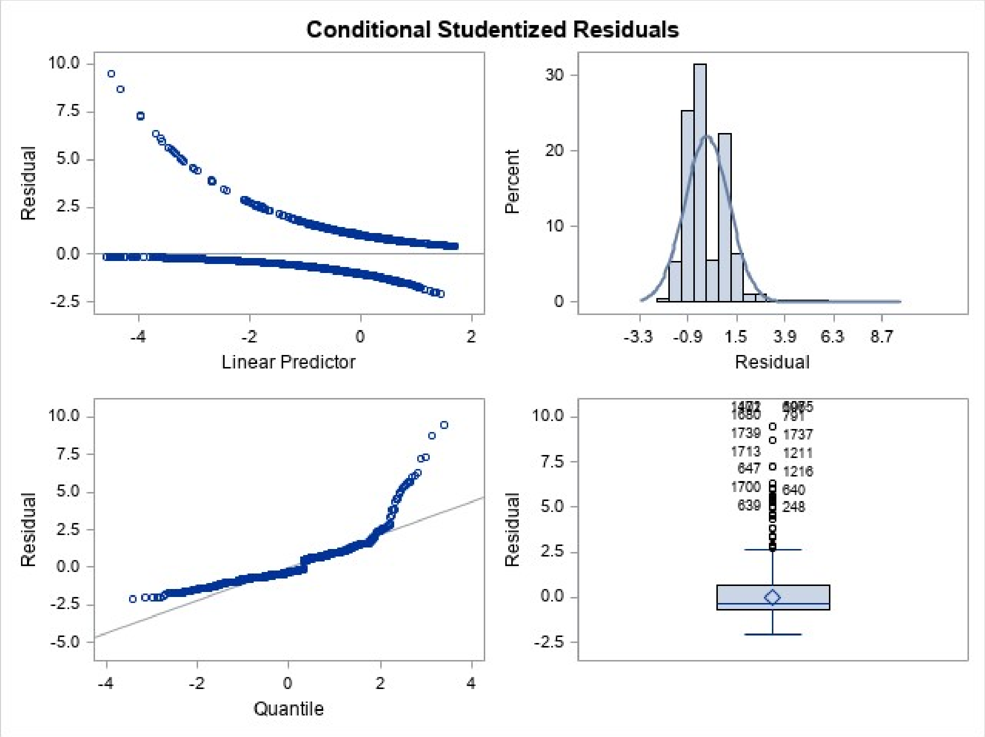
Conditional Studentized Residuals
Residual
Linear Predictor
Percent
Residual
Quantile
1739
1713
647
1700
639
1737
1211
1216
640
248

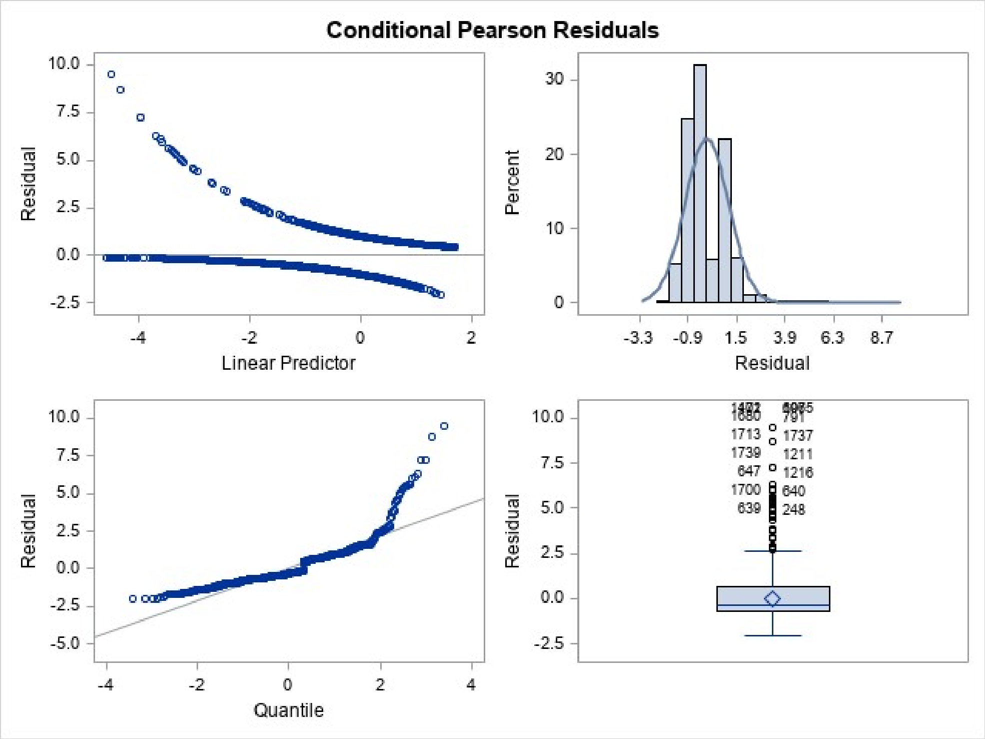
Conditional Pearson Residuals
Residual
Linear Predictor
Percent
Residual
Quantile
1713
1739
647
1700
639
1737
1211
1216
640
248

MODEL C: RIGOUR AND STRENGTH OF RECOMMENDATION ONLY - FIXED-EFFECTS ONLY

The LOGISTIC Procedure

Model Information	
Data Set	WORK.GLMMOUT
Response Variable	differenceinrating
Number of Response Levels	2
Model	binary logit
Optimization Technique	Fisher's scoring

Number of Observations Read	1905
Number of Observations Used	1905

Response Profile		
Ordered Value	differenceinrating	Total Frequency
1	No	1189
2	Yes	716

Probability modeled is differenceinrating='Yes'.

Score Test for Global Null Hypothesis		
Chi-Square	DF	Pr > ChiSq
0.0000	0	<.0001

ROC Model: GLIMMIX model

ROC Model Information		
ROC Contrast Coefficients	predprob	Mu

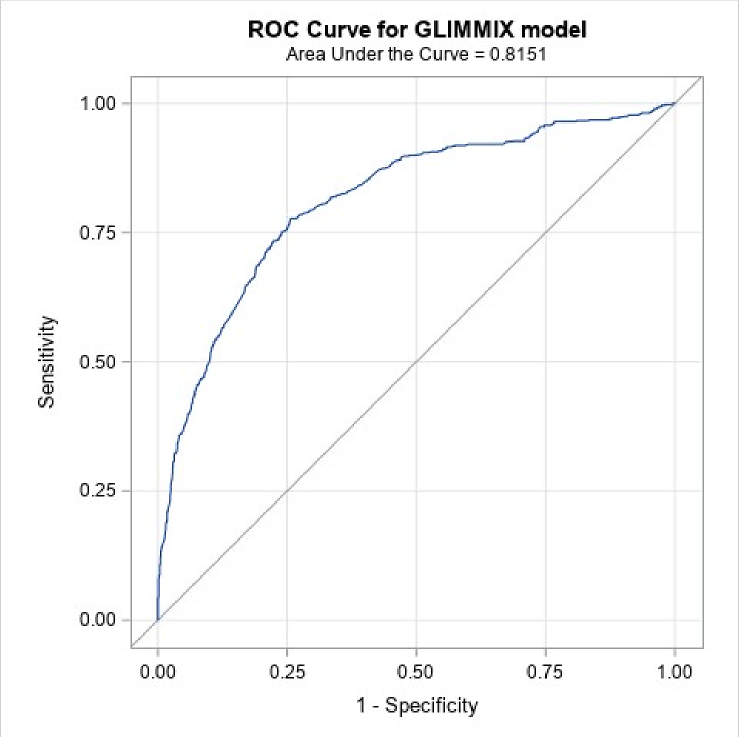

ROC Association Statistics							
	Mann-Whitney						
ROC Model	Area	Standard Error	95% Wald Confidence Limits		Somers' D	Gamma	Tau-a
GLIMMIX model	0.7190	0.0117	0.6960	0.7420	0.4380	0.4503	0.2056

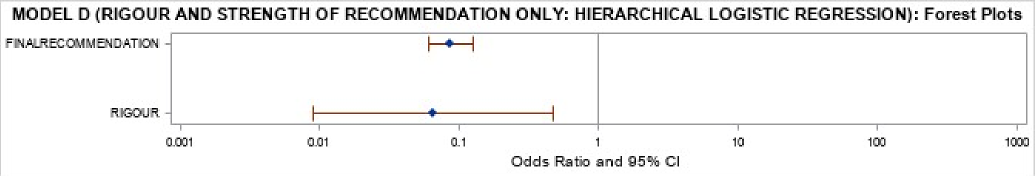
MODEL D (RIGOUR AND STRENGTH OF RECOMMENDATION ONLY: HIERARCHICAL LOGISTIC REGRESSION): Forest Plots
FINALRECOMMENDATION
RIGOUR
0.001
0.01
0.1
1
10
100
1000
Odds Ratio and 95% CI

MODEL D: RIGOUR AND STRENGTH OF RECOMMENDATION ONLY: HIERARCHICAL LOGISTIC REGRESSION

The GLIMMIX Procedure

Model Information	
Data Set	WORK.DATAFORANALYSIS
Response Variable	differenceinrating
Response Distribution	Binary
Link Function	Logit
Variance Function	Default
Variance Matrix Blocked By	guidelineid
Estimation Technique	Maximum Likelihood
Likelihood Approximation	Laplace
Degrees of Freedom Method	Containment

Number of Observations Read	1905
Number of Observations Used	1905

Response Profile		
Ordered Value	differenceinrating	Total Frequency
1	No	1189
2	Yes	716

The GLIMMIX procedure is modeling the probability that differenceinrating='Yes'.

Dimensions	
G-side Cov. Parameters	1
Columns in X	4
Columns in Z per Subject	1
Subjects (Blocks in V)	63
Max Obs per Subject	152

Optimization Information	
Optimization Technique	Dual Quasi-Newton
Parameters in Optimization	4
Lower Boundaries	1
Upper Boundaries	0
Fixed Effects	Not Profiled
Starting From	GLM estimates

Iteration History

Iteration	Restarts	Evaluations	Objective Function	Change	Max Gradient
0	0	4	2097.8485117	.	27.09856
1	0	2	2089.5271973	8.32131434	11.84744
2	0	3	2089.2931027	0.23409464	11.14466
3	0	4	2088.244677	1.04842568	8.178495

4	0	4	2084.21162	4.03305702	8.223983
5	0	2	2083.1329301	1.07868988	1.892244
6	0	3	2083.0284308	0.10449934	0.565085
7	0	3	2083.0258381	0.00259269	0.082982
8	0	3	2083.0257246	0.00011352	0.007428
9	0	3	2083.0257239	0.00000072	0.000963

Convergence criterion (GCONV=1E-8) satisfied.

Fit Statistics	
-2 Log Likelihood	2083.03
AIC (smaller is better)	2091.03
AICC (smaller is better)	2091.05
BIC (smaller is better)	2099.60
CAIC (smaller is better)	2103.60
HQIC (smaller is better)	2094.40

Fit Statistics for Conditional Distribution	
-2 log L(differenceinrating | r. effects)	1962.75
Pearson Chi-Square	2238.72
Pearson Chi-Square / DF	1.18

Covariance Parameter Estimates

Cov Parm	Subject	Estimate	Standard Error	Z Value	Pr > Z
Intercept	guidelineid	0.6298	0.1893	3.33	0.0004

Solutions for Fixed Effects

Effect	FinalRecommendation	Estimate	Standard Error	DF	t Value	Pr > |t|	Alpha	Lower	Upper
Intercept		1.0842	0.4406	62	2.46	0.0167	0.05	0.2034	1.9650
rigour		-2.7226	1.0106	1840	-2.69	0.0071	0.05	-4.7047	-0.7406
FinalRecommendation	Strong	-2.4483	0.1893	1840	-12.93	<.0001	0.05	-2.8197	-2.0770
FinalRecommendation	Weak	0	.	.	.	.	.	.	.

Type III Tests of Fixed Effects

Effect	Num DF	Den DF	F Value	Pr > F
rigour	1	1840	7.26	0.0071
FinalRecommendation	1	1840	167.22	<.0001

Solution for Random Effects

Effect	Subject	Estimate	Std Err Pred	DF	t Value	Pr > |t|	Alpha	Lower	Upper
Intercept	guidelineid 1	-0.5769	0.6134	1840	-0.94	0.3470	0.05	-1.7799	0.6261
Intercept	guidelineid 2	-0.7512	0.2368	1840	-3.17	0.0015	0.05	-1.2155	-0.2868
Intercept	guidelineid 3	1.1279	0.4583	1840	2.46	0.0139	0.05	0.2291	2.0267
Intercept	guidelineid 4	0.7896	0.2933	1840	2.69	0.0072	0.05	0.2143	1.3649
Intercept	guidelineid 5	-0.2306	0.5037	1840	-0.46	0.6471	0.05	-1.2184	0.7572
Intercept	guidelineid 6	-0.5614	0.6843	1840	-0.82	0.4121	0.05	-1.9035	0.7808
Intercept	guidelineid 7	-0.3987	0.2824	1840	-1.41	0.1581	0.05	-0.9526	0.1551

**Intercept**	guidelineid 8	0.06753	0.6124	1840	0.11	0.9122	0.05	-1.1335	1.2686
**Intercept**	guidelineid 9	0.3553	0.4004	1840	0.89	0.3749	0.05	-0.4299	1.1406
**Intercept**	guidelineid 10	0.2901	0.3349	1840	0.87	0.3865	0.05	-0.3667	0.9468
**Intercept**	guidelineid 11	-0.6190	0.5905	1840	-1.05	0.2947	0.05	-1.7772	0.5392
**Intercept**	guidelineid 12	-0.9521	0.4381	1840	-2.17	0.0299	0.05	-1.8113	-0.09295
**Intercept**	guidelineid 13	-0.1948	0.7446	1840	-0.26	0.7936	0.05	-1.6552	1.2655
**Intercept**	guidelineid 14	-0.5669	0.4361	1840	-1.30	0.1937	0.05	-1.4222	0.2883
**Intercept**	guidelineid 15	-0.4364	0.6152	1840	-0.71	0.4782	0.05	-1.6429	0.7701
**Intercept**	guidelineid 16	0.9124	0.3898	1840	2.34	0.0194	0.05	0.1479	1.6769
**Intercept**	guidelineid 17	0.3169	0.3585	1840	0.88	0.3767	0.05	-0.3861	1.0200
**Intercept**	guidelineid 18	0.1513	0.4800	1840	0.32	0.7526	0.05	-0.7901	1.0927
**Intercept**	guidelineid 19	0.5840	0.3693	1840	1.58	0.1140	0.05	-0.1403	1.3084
**Intercept**	guidelineid 20	0.7130	0.3244	1840	2.20	0.0281	0.05	0.07672	1.3493
**Intercept**	guidelineid 21	-0.1503	0.3776	1840	-0.40	0.6906	0.05	-0.8909	0.5902
**Intercept**	guidelineid 22	-0.6327	0.3756	1840	-1.68	0.0923	0.05	-1.3695	0.1040
**Intercept**	guidelineid 23	-0.3515	0.5142	1840	-0.68	0.4944	0.05	-1.3600	0.6571
**Intercept**	guidelineid 24	-0.8096	0.4276	1840	-1.89	0.0585	0.05	-1.6482	0.02899
**Intercept**	guidelineid 25	1.1919	0.3470	1840	3.43	0.0006	0.05	0.5113	1.8724
**Intercept**	guidelineid 26	-0.9411	0.4139	1840	-2.27	0.0231	0.05	-1.7528	-0.1294
**Intercept**	guidelineid 27	-0.3649	0.3761	1840	-0.97	0.3320	0.05	-1.1025	0.3727
**Intercept**	guidelineid 28	0.9558	0.5049	1840	1.89	0.0585	0.05	-0.03442	1.9461
**Intercept**	guidelineid 29	-0.5106	0.6031	1840	-0.85	0.3973	0.05	-1.6934	0.6722
**Intercept**	guidelineid 30	-0.5549	0.6838	1840	-0.81	0.4172	0.05	-1.8961	0.7863
**Intercept**	guidelineid 31	0.5349	0.2628	1840	2.04	0.0419	0.05	0.01957	1.0503
**Intercept**	guidelineid 32	-0.4998	0.2732	1840	-1.83	0.0675	0.05	-1.0356	0.03595
**Intercept**	guidelineid 33	0.2040	0.4561	1840	0.45	0.6548	0.05	-0.6906	1.0986
**Intercept**	guidelineid 34	0.6402	0.6237	1840	1.03	0.3048	0.05	-0.5829	1.8634
**Intercept**	guidelineid 35	-1.7181	0.2632	1840	-6.53	<.0001	0.05	-2.2343	-1.2019
**Intercept**	guidelineid 36	-0.5228	0.4367	1840	-1.20	0.2313	0.05	-1.3792	0.3336
**Intercept**	guidelineid 37	0.1780	0.4593	1840	0.39	0.6984	0.05	-0.7228	1.0788
**Intercept**	guidelineid 38	-0.1935	0.4279	1840	-0.45	0.6512	0.05	-1.0326	0.6457
**Intercept**	guidelineid 39	0.4878	0.3757	1840	1.30	0.1943	0.05	-0.2490	1.2245
**Intercept**	guidelineid 40	0.4387	0.4227	1840	1.04	0.2995	0.05	-0.3903	1.2677
**Intercept**	guidelineid 41	0.03842	0.2435	1840	0.16	0.8747	0.05	-0.4391	0.5160
**Intercept**	guidelineid 42	1.1459	0.3661	1840	3.13	0.0018	0.05	0.4279	1.8640
**Intercept**	guidelineid 43	-0.5947	0.4932	1840	-1.21	0.2280	0.05	-1.5620	0.3726
**Intercept**	guidelineid 44	-0.7244	0.4846	1840	-1.49	0.1351	0.05	-1.6748	0.2260
**Intercept**	guidelineid 45	0.04883	0.2844	1840	0.17	0.8637	0.05	-0.5090	0.6067
**Intercept**	guidelineid 46	0.3909	0.5191	1840	0.75	0.4515	0.05	-0.6271	1.4090
**Intercept**	guidelineid 47	0.8848	0.5083	1840	1.74	0.0819	0.05	-0.1122	1.8817
**Intercept**	guidelineid 48	0.07660	0.3211	1840	0.24	0.8114	0.05	-0.5531	0.7063
**Intercept**	guidelineid 49	-0.00681	0.3971	1840	-0.02	0.9863	0.05	-0.7855	0.7719
**Intercept**	guidelineid 50	-0.9009	0.2881	1840	-3.13	0.0018	0.05	-1.4659	-0.3359
**Intercept**	guidelineid 51	0.7181	0.3510	1840	2.05	0.0409	0.05	0.02980	1.4064
**Intercept**	guidelineid 52	-0.4041	0.5622	1840	-0.72	0.4724	0.05	-1.5066	0.6985
**Intercept**									

	guidelineid 53	-0.1834	0.5696	1840	-0.32	0.7475	0.05	-1.3005	0.9337
**Intercept**	guidelineid 54	-1.0085	0.5598	1840	-1.80	0.0718	0.05	-2.1064	0.08944
**Intercept**	guidelineid 55	-0.2033	0.6446	1840	-0.32	0.7525	0.05	-1.4676	1.0609
**Intercept**	guidelineid 56	0.8628	0.5767	1840	1.50	0.1348	0.05	-0.2683	1.9938
**Intercept**	guidelineid 57	0.1035	0.6759	1840	0.15	0.8783	0.05	-1.2222	1.4292
**Intercept**	guidelineid 58	0.5344	0.5808	1840	0.92	0.3577	0.05	-0.6047	1.6734
**Intercept**	guidelineid 59	0.4725	0.5201	1840	0.91	0.3638	0.05	-0.5476	1.4925
**Intercept**	guidelineid 60	0.7448	0.6056	1840	1.23	0.2189	0.05	-0.4430	1.9326
**Intercept**	guidelineid 61	0.7136	0.5085	1840	1.40	0.1607	0.05	-0.2838	1.7109
**Intercept**	guidelineid 62	0.09066	0.5739	1840	0.16	0.8745	0.05	-1.0348	1.2162
**Intercept**	guidelineid 63	0.3107	0.6215	1840	0.50	0.6172	0.05	-0.9083	1.5296

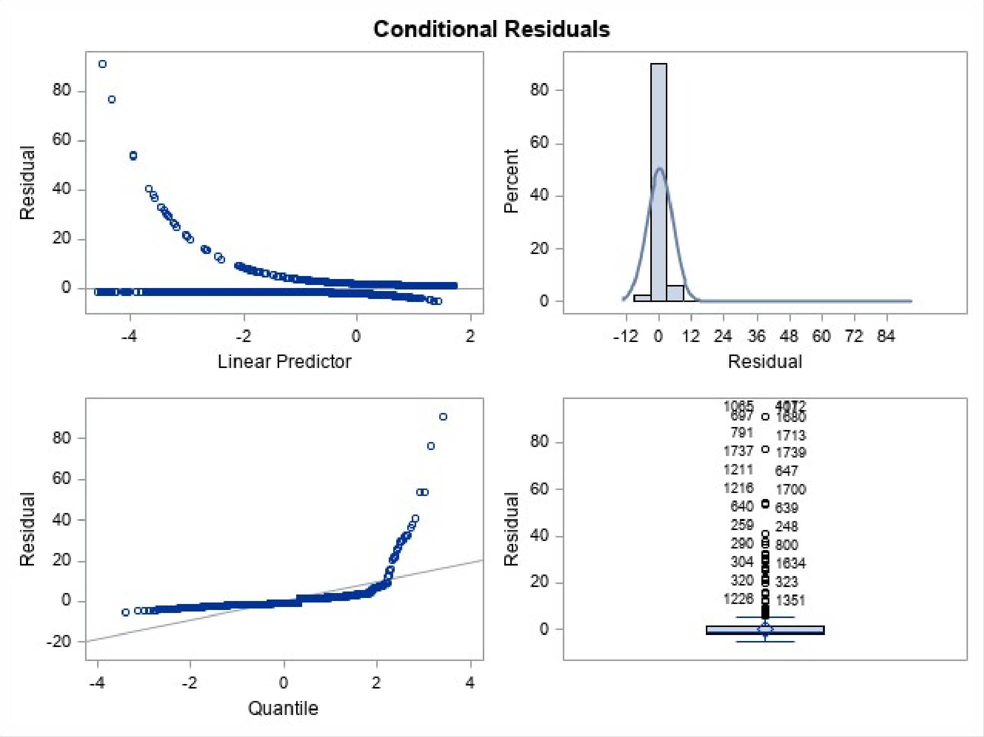

Conditional Studentized Residuals

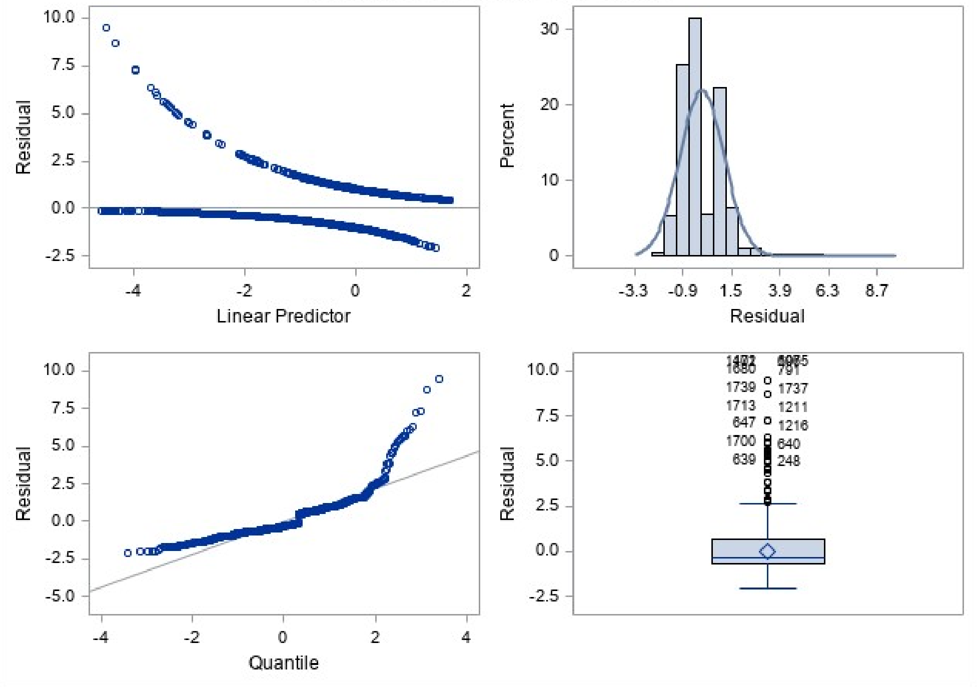

Conditional Pearson Residuals

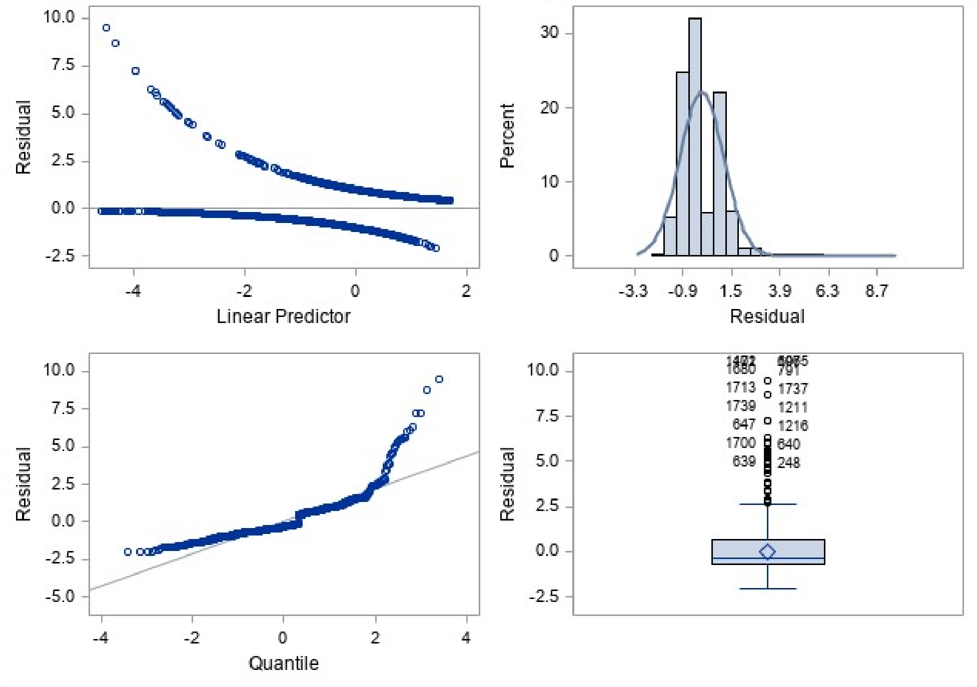

MODEL D: RIGOUR AND STRENGTH OF RECOMMENDATION ONLY: HIERARCHICAL LOGISTIC REGRESSION

The LOGISTIC Procedure

Model Information	
Data Set	WORK.GLMMOUT
Response Variable	differenceinrating
Number of Response Levels	2
Model	binary logit
Optimization Technique	Fisher's scoring

Number of Observations Read	1905
Number of Observations Used	1905

Response Profile		
Ordered Value	differenceinrating	Total Frequency
1	No	1189
2	Yes	716

Probability modeled is differenceinrating='Yes'.

Score Test for Global Null Hypothesis		
Chi-Square	DF	Pr > ChiSq
0.0000	0	<.0001

ROC Model: GLIMMIX model

ROC Model Information		
ROC Contrast Coefficients	predprob	Mu

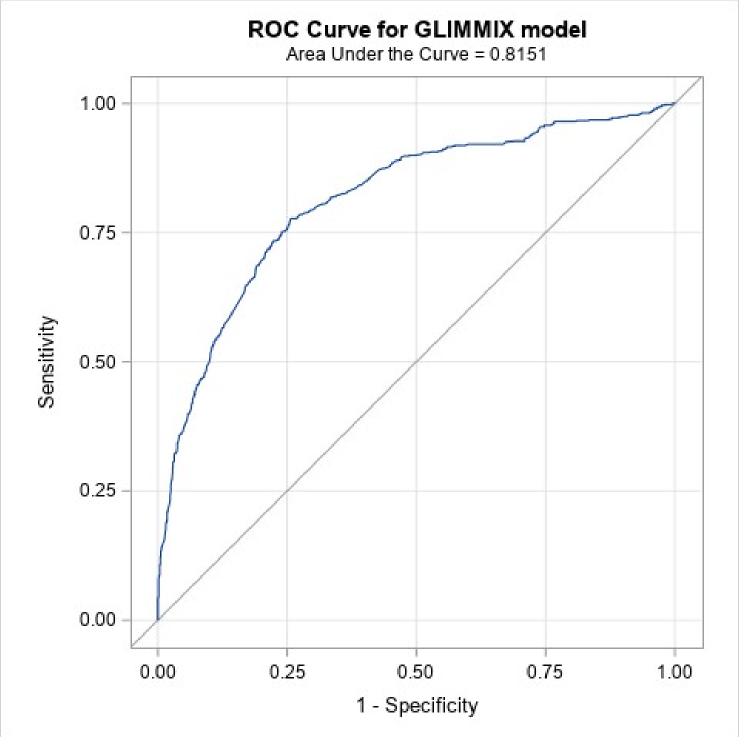

ROC Association Statistics							
	Mann-Whitney						
ROC Model	Area	Standard Error	95% Wald Confidence Limits		Somers' D	Gamma	Tau-a
GLIMMIX model	0.8151	0.0103	0.7948	0.8353	0.6301	0.6321	0.2958

MODEL D (RIGOUR AND STRENGTH OF RECOMMENDATION ONLY: HIERARCHICAL LOGISTIC REGRESSION): Forest Plots

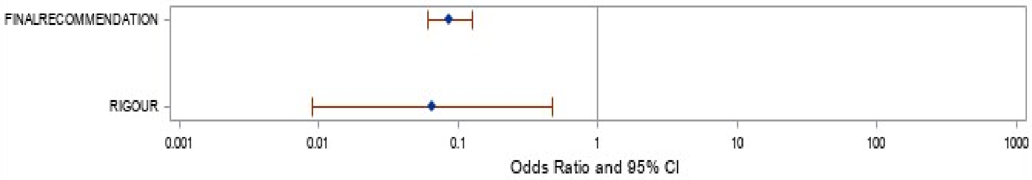

MODEL D: RIGOUR AND STRENGTH OF RECOMMENDATION ONLY: HIERARCHICAL LOGISTIC REGRESSION

The GLIMMIX Procedure

Model Information	
Data Set	WORK.DATAFORANALYSIS
Response Variable	differenceinrating
Response Distribution	Binary
Link Function	Logit
Variance Function	Default
Variance Matrix Blocked By	guidelineid
Estimation Technique	Maximum Likelihood
Likelihood Approximation	Laplace
Degrees of Freedom Method	Containment

Number of Observations Read	1905
Number of Observations Used	1905

Response Profile		
Ordered Value	differenceinrating	Total Frequency
1	No	1189
2	Yes	716

The GLIMMIX procedure is modeling the probability that differenceinrating='Yes'.

Dimensions	
G-side Cov. Parameters	1
Columns in X	4
Columns in Z per Subject	1
Subjects (Blocks in V)	63
Max Obs per Subject	152

Optimization Information	
Optimization Technique	Dual Quasi-Newton
Parameters in Optimization	4
Lower Boundaries	1
Upper Boundaries	0
Fixed Effects	Not Profiled
Starting From	GLM estimates

Iteration History

Iteration	Restarts	Evaluations	Objective Function	Change	Max Gradient
0	0	4	2097.8485117	.	27.09856
1	0	2	2089.5271973	8.32131434	11.84744
2	0	3	2089.2931027	0.23409464	11.14466
3	0	4	2088.244677	1.04842568	8.178495

4	0	4	2084.21162	4.03305702	8.223983
5	0	2	2083.1329301	1.07868988	1.892244
6	0	3	2083.0284308	0.10449934	0.565085
7	0	3	2083.0258381	0.00259269	0.082982
8	0	3	2083.0257246	0.00011352	0.007428
9	0	3	2083.0257239	0.00000072	0.000963

Convergence criterion (GCONV=1E-8) satisfied.

Fit Statistics	
-2 Log Likelihood	2083.03
AIC (smaller is better)	2091.03
AICC (smaller is better)	2091.05
BIC (smaller is better)	2099.60
CAIC (smaller is better)	2103.60
HQIC (smaller is better)	2094.40

Fit Statistics for Conditional Distribution	
-2 log L(differenceinrating | r. effects)	1962.75
Pearson Chi-Square	2238.72
Pearson Chi-Square / DF	1.18

Covariance Parameter Estimates

Cov Parm	Subject	Estimate	Standard Error	Z Value	Pr > Z
Intercept	guidelineid	0.6298	0.1893	3.33	0.0004

Solutions for Fixed Effects

Effect	FinalRecommendation	Estimate	Standard Error	DF	t Value	Pr > |t|	Alpha	Lower	Upper
Intercept		1.0842	0.4406	62	2.46	0.0167	0.05	0.2034	1.9650
rigour		-2.7226	1.0106	1840	-2.69	0.0071	0.05	-4.7047	-0.7406
FinalRecommendation	Strong	-2.4483	0.1893	1840	-12.93	<.0001	0.05	-2.8197	-2.0770
FinalRecommendation	Weak	0	.	.	.	.	.	.	.

Type III Tests of Fixed Effects

Effect	Num DF	Den DF	F Value	Pr > F
rigour	1	1840	7.26	0.0071
FinalRecommendation	1	1840	167.22	<.0001

Solution for Random Effects

Effect	Subject	Estimate	Std Err Pred	DF	t Value	Pr > |t|	Alpha	Lower	Upper
Intercept	guidelineid 1	-0.5769	0.6134	1840	-0.94	0.3470	0.05	-1.7799	0.6261
Intercept	guidelineid 2	-0.7512	0.2368	1840	-3.17	0.0015	0.05	-1.2155	-0.2868
Intercept	guidelineid 3	1.1279	0.4583	1840	2.46	0.0139	0.05	0.2291	2.0267
Intercept	guidelineid 4	0.7896	0.2933	1840	2.69	0.0072	0.05	0.2143	1.3649
Intercept	guidelineid 5	-0.2306	0.5037	1840	-0.46	0.6471	0.05	-1.2184	0.7572
Intercept	guidelineid 6	-0.5614	0.6843	1840	-0.82	0.4121	0.05	-1.9035	0.7808
Intercept	guidelineid 7	-0.3987	0.2824	1840	-1.41	0.1581	0.05	-0.9526	0.1551

**Intercept**	guidelineid 8	0.06753	0.6124	1840	0.11	0.9122	0.05	-1.1335	1.2686
**Intercept**	guidelineid 9	0.3553	0.4004	1840	0.89	0.3749	0.05	-0.4299	1.1406
**Intercept**	guidelineid 10	0.2901	0.3349	1840	0.87	0.3865	0.05	-0.3667	0.9468
**Intercept**	guidelineid 11	-0.6190	0.5905	1840	-1.05	0.2947	0.05	-1.7772	0.5392
**Intercept**	guidelineid 12	-0.9521	0.4381	1840	-2.17	0.0299	0.05	-1.8113	-0.09295
**Intercept**	guidelineid 13	-0.1948	0.7446	1840	-0.26	0.7936	0.05	-1.6552	1.2655
**Intercept**	guidelineid 14	-0.5669	0.4361	1840	-1.30	0.1937	0.05	-1.4222	0.2883
**Intercept**	guidelineid 15	-0.4364	0.6152	1840	-0.71	0.4782	0.05	-1.6429	0.7701
**Intercept**	guidelineid 16	0.9124	0.3898	1840	2.34	0.0194	0.05	0.1479	1.6769
**Intercept**	guidelineid 17	0.3169	0.3585	1840	0.88	0.3767	0.05	-0.3861	1.0200
**Intercept**	guidelineid 18	0.1513	0.4800	1840	0.32	0.7526	0.05	-0.7901	1.0927
**Intercept**	guidelineid 19	0.5840	0.3693	1840	1.58	0.1140	0.05	-0.1403	1.3084
**Intercept**	guidelineid 20	0.7130	0.3244	1840	2.20	0.0281	0.05	0.07672	1.3493
**Intercept**	guidelineid 21	-0.1503	0.3776	1840	-0.40	0.6906	0.05	-0.8909	0.5902
**Intercept**	guidelineid 22	-0.6327	0.3756	1840	-1.68	0.0923	0.05	-1.3695	0.1040
**Intercept**	guidelineid 23	-0.3515	0.5142	1840	-0.68	0.4944	0.05	-1.3600	0.6571
**Intercept**	guidelineid 24	-0.8096	0.4276	1840	-1.89	0.0585	0.05	-1.6482	0.02899
**Intercept**	guidelineid 25	1.1919	0.3470	1840	3.43	0.0006	0.05	0.5113	1.8724
**Intercept**	guidelineid 26	-0.9411	0.4139	1840	-2.27	0.0231	0.05	-1.7528	-0.1294
**Intercept**	guidelineid 27	-0.3649	0.3761	1840	-0.97	0.3320	0.05	-1.1025	0.3727
**Intercept**	guidelineid 28	0.9558	0.5049	1840	1.89	0.0585	0.05	-0.03442	1.9461
**Intercept**	guidelineid 29	-0.5106	0.6031	1840	-0.85	0.3973	0.05	-1.6934	0.6722
**Intercept**	guidelineid 30	-0.5549	0.6838	1840	-0.81	0.4172	0.05	-1.8961	0.7863
**Intercept**	guidelineid 31	0.5349	0.2628	1840	2.04	0.0419	0.05	0.01957	1.0503
**Intercept**	guidelineid 32	-0.4998	0.2732	1840	-1.83	0.0675	0.05	-1.0356	0.03595
**Intercept**	guidelineid 33	0.2040	0.4561	1840	0.45	0.6548	0.05	-0.6906	1.0986
**Intercept**	guidelineid 34	0.6402	0.6237	1840	1.03	0.3048	0.05	-0.5829	1.8634
**Intercept**	guidelineid 35	-1.7181	0.2632	1840	-6.53	<.0001	0.05	-2.2343	-1.2019
**Intercept**	guidelineid 36	-0.5228	0.4367	1840	-1.20	0.2313	0.05	-1.3792	0.3336
**Intercept**	guidelineid 37	0.1780	0.4593	1840	0.39	0.6984	0.05	-0.7228	1.0788
**Intercept**	guidelineid 38	-0.1935	0.4279	1840	-0.45	0.6512	0.05	-1.0326	0.6457
**Intercept**	guidelineid 39	0.4878	0.3757	1840	1.30	0.1943	0.05	-0.2490	1.2245
**Intercept**	guidelineid 40	0.4387	0.4227	1840	1.04	0.2995	0.05	-0.3903	1.2677
**Intercept**	guidelineid 41	0.03842	0.2435	1840	0.16	0.8747	0.05	-0.4391	0.5160
**Intercept**	guidelineid 42	1.1459	0.3661	1840	3.13	0.0018	0.05	0.4279	1.8640
**Intercept**	guidelineid 43	-0.5947	0.4932	1840	-1.21	0.2280	0.05	-1.5620	0.3726
**Intercept**	guidelineid 44	-0.7244	0.4846	1840	-1.49	0.1351	0.05	-1.6748	0.2260
**Intercept**	guidelineid 45	0.04883	0.2844	1840	0.17	0.8637	0.05	-0.5090	0.6067
**Intercept**	guidelineid 46	0.3909	0.5191	1840	0.75	0.4515	0.05	-0.6271	1.4090
**Intercept**	guidelineid 47	0.8848	0.5083	1840	1.74	0.0819	0.05	-0.1122	1.8817
**Intercept**	guidelineid 48	0.07660	0.3211	1840	0.24	0.8114	0.05	-0.5531	0.7063
**Intercept**	guidelineid 49	-0.00681	0.3971	1840	-0.02	0.9863	0.05	-0.7855	0.7719
**Intercept**	guidelineid 50	-0.9009	0.2881	1840	-3.13	0.0018	0.05	-1.4659	-0.3359
**Intercept**	guidelineid 51	0.7181	0.3510	1840	2.05	0.0409	0.05	0.02980	1.4064
**Intercept**	guidelineid 52	-0.4041	0.5622	1840	-0.72	0.4724	0.05	-1.5066	0.6985
**Intercept**									

	guidelineid 53	-0.1834	0.5696	1840	-0.32	0.7475	0.05	-1.3005	0.9337
Intercept	guidelineid 54	-1.0085	0.5598	1840	-1.80	0.0718	0.05	-2.1064	0.08944
Intercept	guidelineid 55	-0.2033	0.6446	1840	-0.32	0.7525	0.05	-1.4676	1.0609
Intercept	guidelineid 56	0.8628	0.5767	1840	1.50	0.1348	0.05	-0.2683	1.9938
Intercept	guidelineid 57	0.1035	0.6759	1840	0.15	0.8783	0.05	-1.2222	1.4292
Intercept	guidelineid 58	0.5344	0.5808	1840	0.92	0.3577	0.05	-0.6047	1.6734
Intercept	guidelineid 59	0.4725	0.5201	1840	0.91	0.3638	0.05	-0.5476	1.4925
Intercept	guidelineid 60	0.7448	0.6056	1840	1.23	0.2189	0.05	-0.4430	1.9326
Intercept	guidelineid 61	0.7136	0.5085	1840	1.40	0.1607	0.05	-0.2838	1.7109
Intercept	guidelineid 62	0.09066	0.5739	1840	0.16	0.8745	0.05	-1.0348	1.2162
Intercept	guidelineid 63	0.3107	0.6215	1840	0.50	0.6172	0.05	-0.9083	1.5296

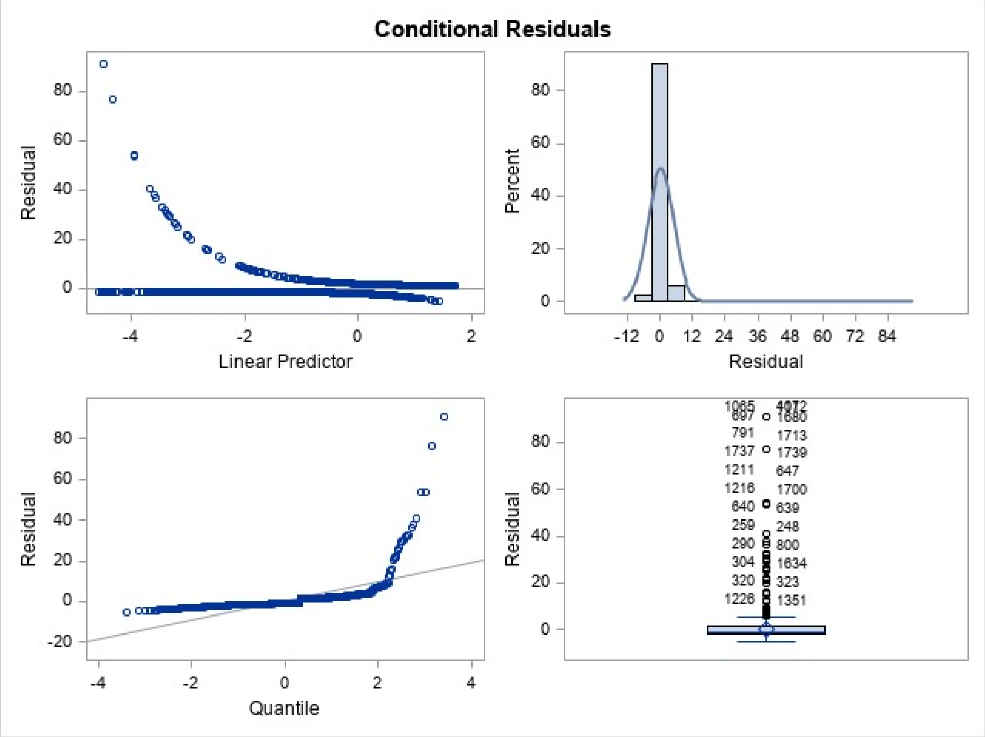

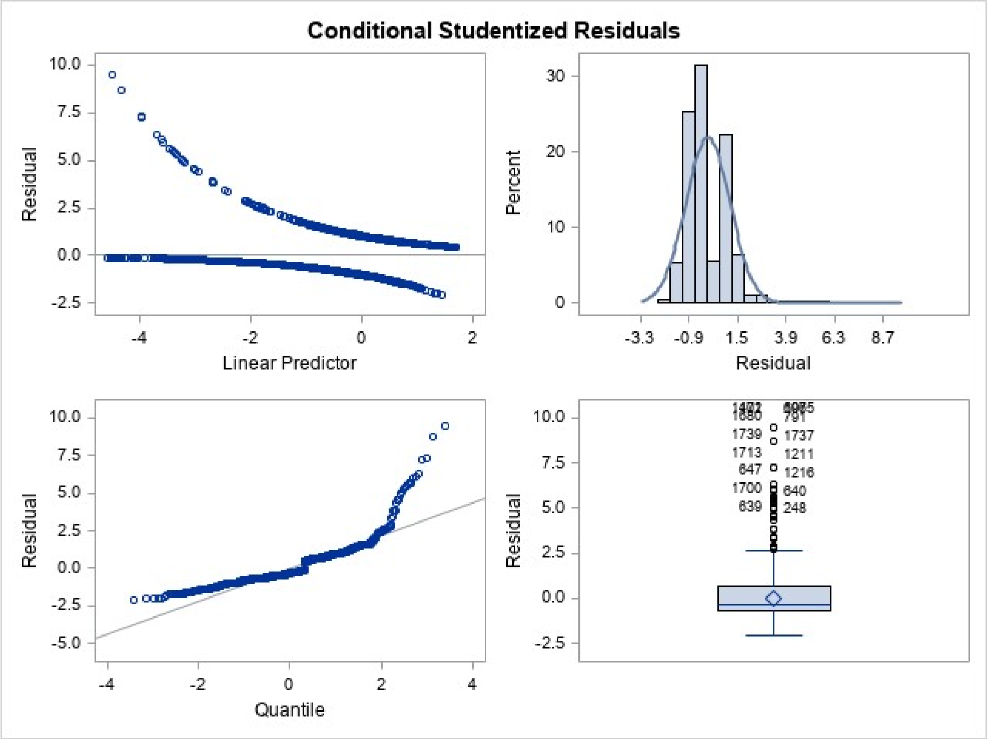
Conditional Studentized Residuals
Residual
Linear Predictor
Percent
Residual
Quantile

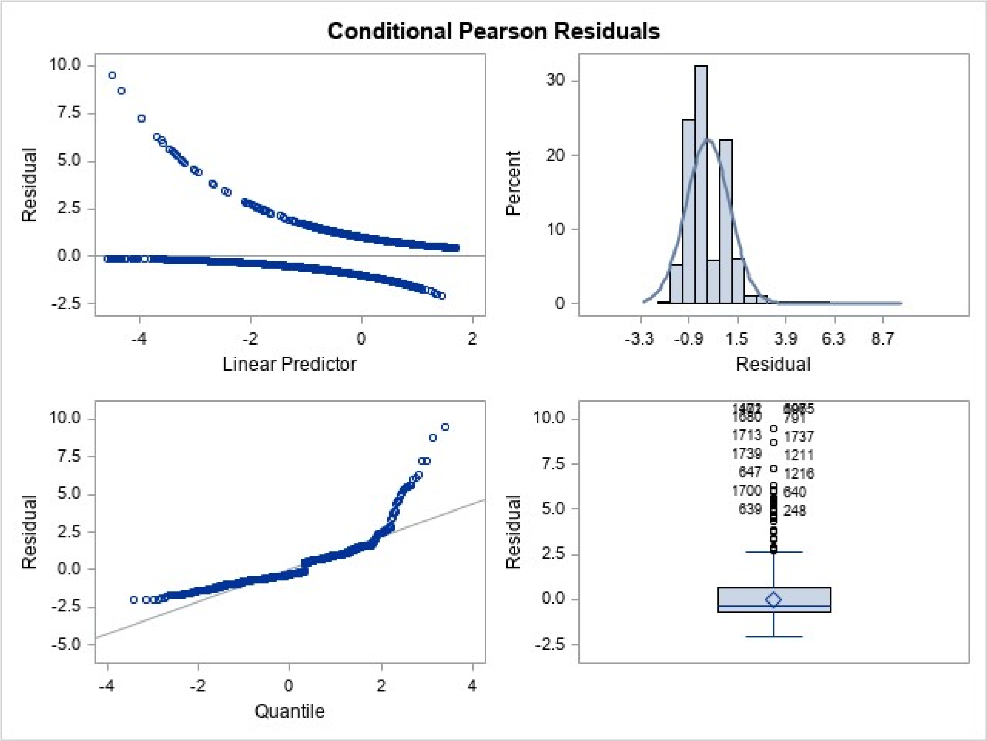
Conditional Pearson Residuals
Residual
Linear Predictor
Percent
Residual
Quantile

MODEL D: RIGOUR AND STRENGTH OF RECOMMENDATION ONLY: HIERARCHICAL LOGISTIC REGRESSION

The LOGISTIC Procedure

Model Information	
Data Set	WORK.GLMMOUT
Response Variable	differenceinrating
Number of Response Levels	2
Model	binary logit
Optimization Technique	Fisher's scoring

Number of Observations Read	1905
Number of Observations Used	1905

Response Profile		
Ordered Value	differenceinrating	Total Frequency
1	No	1189
2	Yes	716

Probability modeled is differenceinrating='Yes'.

Score Test for Global Null Hypothesis		
Chi-Square	DF	Pr > ChiSq
0.0000	0	<.0001

ROC Model: GLIMMIX model

ROC Model Information		
ROC Contrast Coefficients	predprob	Mu

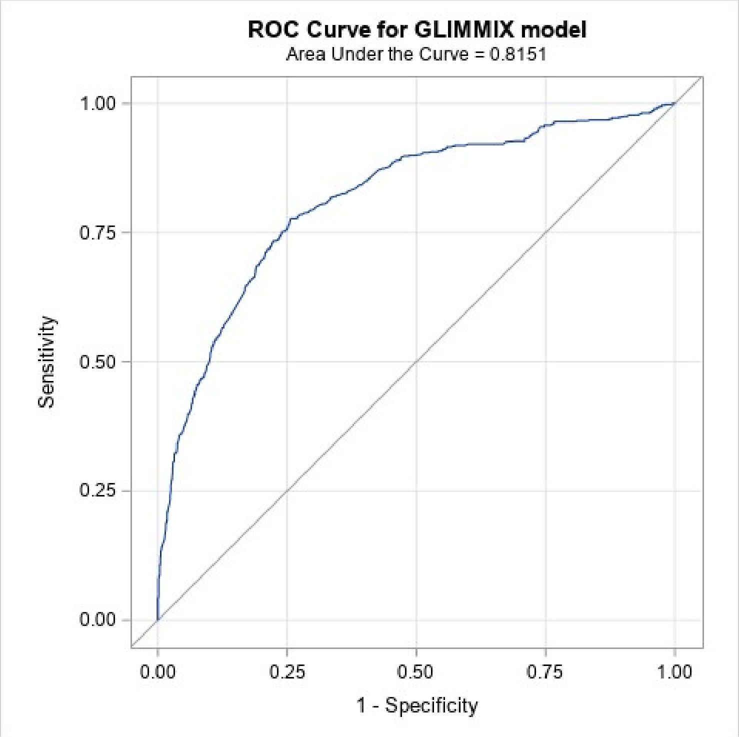

ROC Association Statistics							
	Mann-Whitney						
ROC Model	Area	Standard Error	95% Wald Confidence Limits		Somers' D	Gamma	Tau-a
GLIMMIX model	0.8151	0.0103	0.7948	0.8353	0.6301	0.6321	0.2958

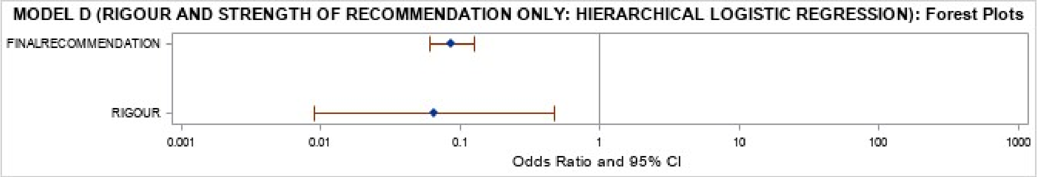
MODEL D (RIGOUR AND STRENGTH OF RECOMMENDATION ONLY: HIERARCHICAL LOGISTIC REGRESSION): Forest Plots
FINALRECOMMENDATION
RIGOUR
0.001
0.01
0.1
1
10
100
1000
Odds Ratio and 95% CI
